# Supplementary material for: Occurrence of sensitive topics during ward round: an ancillary analysis of the BEDSIDE-OUTSIDE trial
Source: BMJ Open. 2023 Sep 21;13(9):e073584. doi: 10.1136/bmjopen-2023-073584 (PMC10514661; doi:10.1136/bmjopen-2023-073584)
Supplement: Supplementary data [file bmjopen-2023-073584supp001.pdf]

BMJ Publishing Group Limited (BMJ) disclaims all liability and responsibility arising from any reliance placed on this supplemental material which has been supplied by the author(s)

Supplementary Appendix

Supplement 1. Associations of patient characteristics with sensitive topics

	n	All	No Sensitive topics	Sensitive topics	*p-value*	univariable OR (95%CI)	*p-value*	OR (95%CI) *adjusted for centrum, intervention*	*p-value*
**n**			445	474					
**Sociodemographic factors**									
Age, years; mean (SD)	919	65.0 (15.9)	65.7 (15.9)	64.3 (15.9)	*0.16*	0.99 (0.99, 1)	*0.164*	1 (0.99, 1)	*0.304*
Female sex, n (%)	919	361 (39.3%)	173 (38.9%)	188 (39.7%)	*0.81*	1.03 (0.79, 1.35)	*0.807*	1.05 (0.8, 1.37)	*0.741*
Number of children, mean (SD)	919	2.5 (9.2)	2.1 (6.7)	2.8 (11.0)	*0.26*	1.01 (0.99, 1.02)	*0.275*	1.01 (0.99, 1.02)	*0.365*
**Family status**	919								
Single, relationship, married, registered partnership, n (%)		648 (70.5%)	317 (71.2%)	331 (69.8%)	*0.84*	1 (ref.)			
Separated, divorced, n (%)		147 (16.0%)	68 (15.3%)	79 (16.7%)		1.11 (0.78, 1.59)	*0.56*	1.05 (0.73, 1.52)	*0.787*
Widowed, n (%)		124 (13.5%)	60 (13.5%)	64 (13.5%)		1.02 (0.7, 1.5)	*0.913*	1.07 (0.72, 1.59)	*0.738*
**Citizenship**	919								
Switzerland, n (%)		789 (85.9%)	386 (86.7%)	403 (85.0%)	*0.71*	1 (ref.)			
Germany, n (%)		55 (6.0%)	26 (5.8%)	29 (6.1%)		1.07 (0.62, 1.85)	*0.813*	0.97 (0.55, 1.68)	*0.904*
Other, n (%)		75 (8.2%)	33 (7.4%)	42 (8.9%)		1.22 (0.76, 1.96)	*0.416*	1.22 (0.75, 1.98)	*0.421*
**Highest level of education**									
No School graduation, n (%)		23 (2.5%)	10 (2.2%)	13 (2.7%)	*0.86*	1 (ref.)			
School graduation, n (%)		141 (15.3%)	67 (15.1%)	74 (15.6%)		0.85 (0.35, 2.07)	*0.719*	0.96 (0.39, 2.35)	*0.929*
Apprenticeship/Diploma, n (%)		631 (68.7%)	309 (69.4%)	322 (67.9%)		0.8 (0.35, 1.86)	*0.605*	0.92 (0.39, 2.15)	*0.846*
University/Higher Education, n (%)		118 (12.8%)	55 (12.4%)	63 (13.3%)		0.88 (0.36, 2.17)	*0.783*	0.89 (0.36, 2.21)	*0.799*
Missings, n (%)		6 (0.7%)	4 (0.9%)	2 (0.4%)		0.38 (0.06, 2.54)	*0.321*	0.39 (0.06, 2.62)	*0.336*
**Occupation**	919								
Employed / working + IV, n (%)		259 (28.2%)	129 (29.0%)	130 (27.4%)	***0.041***	1 (ref.)			
Unemployed / homemaker, n (%)		39 (4.2%)	13 (2.9%)	26 (5.5%)		1.98 (0.98, 4.03)	*0.058*	2 (0.97, 4.09)	*0.059*
Retired / IV support, n (%)		605 (65.8%)	296 (66.5%)	309 (65.2%)		1.04 (0.77, 1.39)	*0.812*	1.05 (0.78, 1.42)	*0.735*
In education, n (%)		6 (0.7%)	5 (1.1%)	1 (0.2%)		0.2 (0.02, 1.72)	*0.142*	0.19 (0.02, 1.63)	*0.128*
Other, n (%)		10 (1.1%)	2 (0.4%)	8 (1.7%)		3.97 (0.83, 19.05)	*0.085*	3.41 (0.7, 16.56)	*0.128*
**Health-related factors**	919								
Main admission diagnosis									
Coronary heart disease, n (%)		93 (10.1%)	53 (11.9%)	40 (8.4%)	***<0.001***	1 (ref.)			
Congestive heart failure, n (%)		80 (8.7%)	38 (8.5%)	42 (8.9%)		1.46 (0.8, 2.67)	*0.213*	1.7 (0.92, 3.15)	*0.09*
Cardiovascular diseases, n (%)		98 (10.7%)	56 (12.6%)	42 (8.9%)		0.99 (0.56, 1.76)	*0.983*	1.09 (0.61, 1.95)	*0.78*
Infections, n (%)		207 (22.5%)	116 (26.1%)	91 (19.2%)		1.04 (0.63, 1.7)	*0.878*	1.2 (0.73, 1.99)	*0.468*
Gastrointestinal diseases, n (%)		58 (6.3%)	31 (7.0%)	27 (5.7%)		1.15 (0.6, 2.23)	*0.67*	1.15 (0.59, 2.25)	*0.687*
Metabolisms, n (%)		51 (5.5%)	30 (6.7%)	21 (4.4%)		0.93 (0.46, 1.85)	*0.831*	1 (0.49, 2.02)	*0.999*
Tumour, n (%)		81 (8.8%)	36 (8.1%)	45 (9.5%)		1.66 (0.91, 3.02)	*0.1*	1.64 (0.89, 3.04)	*0.115*
Other, n (%)		251 (27.3%)	85 (19.1%)	166 (35.0%)		2.59 (1.59, 4.21)	***<0.001***	3.08 (1.87, 5.07)	***<0.001***
Comorbidities	919								
Charlson Comorbidity Index, mean (SD)		4.45 (2.88)	4.4 (2.8)	4.5 (3.0)	*0.54*	1.01 (0.97, 1.06)	*0.543*	0.76 (0.59, 1)	***0.048***
Cardiology, n (%)		485 (52.8%)	248 (55.7%)	237 (50.0%)	*0.082*	1.02 (0.98, 1.07)	*0.373*	0.86 (0.65, 1.13)	*0.273*
Neurology, n (%)		193 (21.0%)	80 (18.0%)	113 (23.8%)	***0.029***	0.79 (0.61, 1.03)	*0.082*	1.01 (0.77, 1.33)	*0.941*
Rheumatology / Immunology, n (%)		143 (15.6%)	68 (15.3%)	75 (15.8%)	*0.82*	0.82 (0.63, 1.07)	*0.15*	1.03 (0.78, 1.37)	*0.819*
Gastrointestinal, n (%)		254 (27.6%)	118 (26.5%)	136 (28.7%)	*0.46*	1.43 (1.04, 1.97)	***0.03***	1.18 (0.85, 1.65)	*0.325*
Endocrinology, n (%)		352 (38.3%)	185 (41.6%)	167 (35.2%)	***0.048***	1.46 (1.06, 2.03)	*0.022*	1.21 (0.86, 1.71)	*0.264*
Respiratory, n (%)		315 (34.3%)	152 (34.2%)	163 (34.4%)	*0.94*	1.04 (0.73, 1.49)	*0.821*	1.07 (0.81, 1.4)	*0.627*
Infectious diseases, n (%)		171 (18.6%)	77 (17.3%)	94 (19.8%)	*0.33*	1.11 (0.77, 1.62)	*0.57*	1.12 (0.85, 1.48)	*0.416*

Renal, n (%)		317 (34.5%)	150 (33.7%)	167 (35.2%)	0.63	1.12 (0.83, 1.49)	0.461	0.25 (0.07, 0.91)	**0.035**
Gynaecology, n (%)		14 (1.5%)	11 (2.5%)	3 (0.6%)	**0.023**	1.18 (0.87, 1.58)	0.284	0.26 (0.07, 0.94)	**0.04**
Urology, n (%)		93 (10.1%)	43 (9.7%)	50 (10.5%)	0.66	1.1 (0.72, 1.69)	0.657	1.24 (0.8, 1.93)	0.338
Oncology, n (%)		287 (31.2%)	145 (32.6%)	142 (30.0%)	0.39	0.88 (0.67, 1.17)	0.391	1.81 (1.13, 2.9)	**0.014**
Psychiatry, n (%)		104 (11.3%)	31 (7.0%)	73 (15.4%)	**<0.001**	2.43 (1.56, 3.78)	**<0.001**	1.97 (1.21, 3.2)	**0.006**
Depression, n (%)		82 (8.9%)	29 (6.5%)	53 (11.2%)	**0.013**	2.43 (1.56, 3.78)	**<0.001**	0.89 (0.68, 1.15)	0.364
Other, n (%)		498 (54.2%)	248 (55.7%)	250 (52.7%)	0.36	2.54 (1.62, 3.99)	**<0.001**	0.95 (0.73, 1.25)	0.734
**Health self rating VAS [0-100], mean (SD)**	919	56.9 (22.32)	58.64 (21.97)	55.25 (22.55)	**0.021**	0.99 (0.99, 1)	**0.022**	0.99 (0.99, 1)	**0.018**
**Quality of life (EQ-5D) Index, mean (SD)**	919	0.71 (0.3)	0.75 (0.27)	0.67 (0.3)	**<0.001**	0.38 (0.24, 0.6)	**<0.001**	0.4 (0.25, 0.65)	**<0.001**
**Patient's Decisional Control Preferences**	919								
SDM, n (%)		473 (62.2%)	234 (61.1%)	239 (63.2%)	0.54	1 (ref.)			
active, n (%)		117 (15.4%)	57 (14.9%)	60 (15.9%)	0.70	1.19 (0.84, 1.69)	0.332	1.22 (0.86, 1.75)	0.268
passive, n (%)		171 (22.5%)	92 (24.0%)	79 (20.9%)	0.30	1.23 (0.77, 1.96)	0.397	1.24 (0.77, 2)	0.382
**Intervention of the original study, n %**	919								
Bedside ward round		476 (51.8%)	257 (57.8%)	219 (46.2%)	**<0.001**	1 (ref.)			
Outside the rooum ward round		443 (48.2%)	188 (42.2%)	255 (53.8%)		0.63 (0.48, 0.82)	**<0.001**	0.64 (0.49, 0.83)*	**0.001**

**adjusted for study centre, intervention. Odds ratios were calculated with logistic regression models. Abbreviations: OR, odds ratio; SD, standard deviation; CI, confidence interval; IV, Swiss disability insurance; n, number*

Supplement 2. Associations of communication factors with sensitive topics

	n	No Sensitive topics	Sensitive topics	*p-value*	OR (95%CI)	*p-value*	Adjusted OR (95%CI) *adjusted for centrum, intervention*	*p-value*
		445	474					
**WEMS-Technique communication**	919							
Waiting, n (%)		71 (16.0%)	55 (11.6%)	0.055	0.69 (0.47, 1.01)	0.056	0.83 (0.56, 1.24)	0.37
Echoing, n (%)		108 (24.3%)	107 (22.6%)	0.54	0.91 (0.67, 1.23)	0.544	1.02 (0.74, 1.41)	0.902
Mirroring, n (%)		52 (11.7%)	40 (8.4%)	0.10	0.7 (0.45, 1.08)	0.103	0.82 (0.52, 1.28)	0.377
Summarising, n (%)		44 (9.9%)	39 (8.2%)	0.38	0.82 (0.52, 1.28)	0.381	0.84 (0.53, 1.33)	0.464
Open-ended questions, n (%)		352 (79.1%)	348 (73.4%)	**0.043**	0.73 (0.54, 0.99)	**0.044**	0.77 (0.56, 1.05)	0.102
Confirming, n (%)		211 (47.4%)	195 (41.1%)	0.056	0.78 (0.6, 1.01)	0.056	0.79 (0.61, 1.03)	0.084
Nodding, n (%)		344 (77.3%)	362 (76.4%)	0.74	0.95 (0.7, 1.29)	0.738	1.09 (0.79, 1.49)	0.611
**NURSE-Technique communication in patients with emotions (n = 115)**	115	34	81					
Visible evidence of emotion, n (%)		7 (21%)	16 (20%)	0.92	0.95 (0.35, 2.57)	0.919	0.95 (0.33, 2.78)	0.929
Understanding (verbal/nonverbal), n (%)		8 (24%)	23 (28%)	0.59	1.29 (0.51, 3.26)	0.592	1.24 (0.46, 3.35)	0.667
Respecting, n (%)		4 (12%)	26 (32%)	**0.023**	3.55 (1.13, 11.12)	**0.03**	2.7 (0.79, 9.25)	0.114
Supporting, n (%)		9 (26%)	25 (31%)	0.64	1.24 (0.51, 3.04)	0.638	1.24 (0.48, 3.23)	0.66
Exploring, n (%)		9 (26%)	25 (31%)	0.64	1.24 (0.51, 3.04)	0.638	1.24 (0.48, 3.23)	0.66
Hasty solace, n (%)		0 (0%)	3 (4%)	0.26	n.a.	n.a.	n.a.	n.a.
Distracting/Ignoring, n (%)		0 (0%)	3 (4%)	0.26	n.a.	n.a.	n.a.	n.a.
Dismissing ("that's not so bad"), n (%)		4 (12%)	4 (5%)	0.19	0.39 (0.09, 1.66)	0.202	0.58 (0.12, 2.84)	0.505
Replies with providing information instead of addressing emotion, n (%)		15 (44%)	37 (46%)	0.88	1.07 (0.48, 2.38)	0.878	1.13 (0.48, 2.66)	0.785
Physician talks about emotion, n (%)		4 (12%)	9 (11%)	0.92	0.94 (0.27, 3.28)	0.92	0.79 (0.21, 3.03)	0.734
Overall rating: Responding to emotions, n (%)		14 (44%)	28 (35%)	0.39	0.69 (0.3, 1.6)	0.389	0.64 (0.26, 1.61)	0.346
**Other communication factors**	919							
Occurrence of emotion, n (%)		48 (10.8%)	70 (14.8%)	0.071	1.43 (0.97, 2.12)	0.072	1.35 (0.9, 2.01)	0.144
Medical staff talks to patient about diagnosis, n (%)		215 (48.3%)	271 (57.2%)	**0.007**	1.43 (1.1, 0)	**0.007**	1.59 (1.21, 2.09)	**0.001**
Medical staff talks to patient about symptoms, n (%)		399 (89.7%)	443 (93.5%)	**0.038**	1.65 (1.02, 2.65)	**0.039**	1.66 (1.02, 2.69)	**0.041**
Medical staff talks to patient about treatment, n (%)		334 (75.1%)	383 (80.8%)	**0.036**	1.4 (1.02, 1.91)	**0.036**	1.38 (1, 1.9)	**0.049**
Medical staff talks to patient about next steps, n (%)		344 (77.3%)	431 (90.9%)	**<0.001**	2.94 (2, 4.32)	**<0.001**	2.8 (1.89, 4.13)	**<0.001**
Medical staff talks to patient about social issues, n (%)		272 (61.1%)	320 (67.5%)	**0.043**	1.32 (1.01, 1.73)	0.043	1.43 (1.08, 1.9)	**0.013**
Medical staff talks about patient instead of with patient after case discussion, n (%)		329 (73.9%)	351 (74.1%)	0.97	1.01 (0.75, 1.35)	0.967	1.19 (0.87, 1.62)	0.282
Physician and patient talk at cross purposes, n (%)		17 (3.8%)	35 (7.4%)	**0.019**	2.01 (1.11, 3.64)	**0.022**	2.29 (1.24, 4.25)	**0.008**
Patient seems not to understand something, but does not ask, n (%)		6 (1.3%)	17 (3.6%)	**0.030**	2.72 (1.06, 6.97)	**0.037**	4.15 (1.55, 11.08)	**0.005**
Physician disagrees with / corrects patient, n (%)		30 (6.7%)	57 (12.0%)	**0.006**	1.89 (1.19, 3)	**0.007**	2.05 (1.28, 3.3)	**0.003**
Patient disagrees with / corrects physician, n (%)		27 (6.1%)	53 (11.2%)	**0.006**	1.95 (1.2, 3.16)	**0.007**	2.12 (1.29, 3.48)	**0.003**
Occurrence of *"cue"*, n (%)		32 (7.2%)	79 (16.7%)	**<0.001**	2.58 (1.67, 3.98)	**<0.001**	2.36 (1.52, 3.66)	**<0.001**
Addressing *"cue"* concern, n (%)		19 (4.3%)	40 (8.4%)	**0.010**	2.07 (1.18, 3.63)	**0.011**	1.83 (1.03, 3.24)	**0.039**
Reacts by providing information instead of exploration, n (%)		19 (4.3%)	33 (7.0%)	0.077	1.68 (0.94, 3)	0.08	1.59 (0.88, 2.87)	0.122

**adjusted for study centre, intervention. Odds ratios were calculated with logistic regression models. Abbreviations: OR, odds ratio; CI, confidence interval; cue, current unvoiced element; n, number*

Supplement 3. Association of sensitive topics with various outcomes

	n	No Sensitive topics	Sensitive topics	*p-value*	Difference or OR (95%CI)	*p-value*	Adjusted difference or OR (95%CI) *adjusted for centrum, intervention*	*p-value*
**Primary endpoint**		445	474					
Overall satisfaction (VAS 0-100), mean (SD)	906	90.2 (12.1)	87.7 (14.6)	***0.006***	-2.49 (-4.24, -0.73)	***0.006***	-2.5 (-4.28, -0.72)	***0.006***
**Secondary endpoints**								
**Satisfaction with care (VAS 0-100)**								
Overall satisfaction with ward round, mean (SD)	898	89.9 (16.1)	86.6 (19.9)	***0.006***	-3.33 (-5.71, -0.95)	***0.006***	-3.51 (-5.93, -1.09)	***0.005***
Overall satisfaction with hospital stay, mean (SD)	901	88.3 (17.0)	86.8 (16.7)	*0.18*	-1.52 (-3.72, 0.68)	*0.176*	-1.1 (-3.32, 1.11)	*0.328*
Overall satisfaction with medical care, mean (SD)	891	91.9 (14.1)	90.0 (16.0)	*0.069*	-1.84 (-3.83, 0.14)	*0.069*	-1.97 (-3.99, 0.06)	*0.057*
Overall satisfaction with communication with physicians during ward round, mean (SD)	886	90.7 (15.9)	86.2 (21.7)	***<0.001***	-4.48 (-7.01, -1.95)	***0.001***	-4.39 (-6.97, -1.82)	***0.001***
Overall satisfaction with communication with nursing team during ward round , mean (SD)	622	92.3 (16.6)	90.0 (17.7)	*0.099*	-2.28 (-4.99, 0.43)	*0.099*	-2.45 (-5.21, 0.31)	*0.082*
**Patient knowledge about medical care**								
Average subjective knowledge about their medical care, mean (SD)	919	81.2 (19.0)	77.8 (22.0)	***0.013***	-3.38 (-6.05, -0.71)	***0.013***	-3.86 (-6.57, -1.15)	***0.005***
Subjective understanding of disease, mean (SD)	902	82.7 (19.8)	80.4 (21.8)	*0.092*	-2.34 (-5.07, 0.39)	*0.092*	-2.7 (-5.48, 0.08)	*0.057*
Subjective understanding of therapeutic approach, mean (SD)	861	80.8 (23.3)	76.0 (27.8)	***0.006***	-4.83 (-8.28, -1.38)	***0.006***	-5.77 (-9.25, -2.29)	***0.001***
Subjective understanding of further plans for care, mean (SD)	851	79.8 (25.0)	76.1 (27.8)	***0.042***	-3.71 (-7.27, -0.14)	***0.042***	-3.99 (-7.61, -0.37)	***0.031***
Average objective knowledge about their medical care, mean (SD)	919	72.6 (24.9)	68.7 (25.8)	***0.021***	-3.88 (-7.17, -0.6)	***0.021***	-3.83 (-7.18, -0.48)	*0.025*
Objective understanding of disease, mean (SD)	916	71.5 (32.2)	66.3 (33.4)	***0.016***	-5.22 (-9.48, -0.96)	***0.016***	-5.24 (-9.59, -0.9)	***0.018***
Objective understanding of therapeutic approach, mean (SD)	908	71.3 (34.2)	68.4 (35.0)	*0.21*	-2.9 (-7.42, 1.61)	*0.208*	-2.39 (-6.99, 2.22)	*0.309*
Objective understanding of further plans for care, mean (SD)	916	75.3 (31.5)	71.2 (34.0)	*0.061*	-4.08 (-8.34, 0.19)	*0.061*	-4.4 (-8.73, -0.06)	*0.047*
**Measured timeliness of ward round**								
Duration of outside the room discussions (min), mean (SD)	919	2.8 (3.6)	4.9 (5.0)	***<0.001***	2.17 (1.6, 2.73)	***<0.001***	1.41 (1.02, 1.81)	***<0.001***
Duration of bedside discussions (min), mean (SD)	919	8.2 (4.2)	9.0 (5.0)	***0.005***	0.88 (0.27, 1.48)	***0.005***	1.56 (1.03, 2.09)	***<0.001***
Duration of debriefing outside the room (min), mean (SD)	919	0.4 (1.1)	0.5 (1.1)	*0.063*	0.14 (-0.01, 0.29)	*0.063*	0.16 (0.01, 0.31)	***0.038***
Total duration of ward round per patient (min), mean (SD)	919	11.3 (4.6)	14.5 (5.6)	***<0.001***	3.18 (2.52, 3.85)	***<0.001***	3.13 (2.47, 3.79)	***<0.001***
**Patient perception regarding time spent on ward round**								
Overall duration of ward round was sufficient (VAS 0-100), mean (SD)	859	91.2 (18.3)	89.9 (21.9)	*0.35*	-1.3 (-4, 1.41)	*0.346*	-1.21 (-3.98, 1.55)	*0.389*
Time spent with physicians was sufficient (VAS 0-100), mean (SD)	771	90.2 (18.0)	89.3 (20.0)	*0.52*	-0.88 (-3.58, 1.81)	*0.52*	-0.89 (-3.64, 1.86)	*0.525*
Patients estimation on the time spent with patient on ward round (min), mean (SD)	856	11.2 (6.0)	11.7 (7.1)	*0.27*	0.5 (-0.39, 1.38)	*0.271*	0.94 (0.06, 1.81)	***0.036***
Patients estimation on the time spent per day with patient case overall (min), mean (SD)	719	69.6 (78.0)	73.2 (85.1)	*0.55*	3.63 (-8.34, 15.6)	*0.552*	5.26 (-6.9, 17.42)	*0.396*
The ward round was helpful for better								
- understanding the main illness (VAS 0-100), mean (SD)	773	69.5 (34.3)	62.7 (37.3)	***0.009***	-6.75 (-11.82, -1.69)	***0.009***	-6.21 (-11.4, -1.01)	***0.019***
- further therapeutic measures (VAS 0-100), mean (SD)	714	68.7 (34.6)	60.5 (37.2)	***0.002***	-8.22 (-13.51, -2.92)	***0.002***	-7.77 (-13.19, -2.36)	***0.005***
- further plans of care (VAS 0-100), mean (SD)	755	71.1 (33.9)	69.9 (67.5)	*0.76*	-1.21 (-8.88, 6.47)	*0.758*	-1.49 (-9.33, 6.35)	*0.71*
All my questions were answered (VAS 0-100), mean (SD)	752	93.0 (15.6)	89.7 (21.0)	***0.017***	-3.25 (-5.91, -0.59)	***0.017***	-3.29 (-6.01, -0.57)	***0.018***
I was able to understand all answers to my questions (VAS 0-100), mean (SD)	747	93.3 (15.3)	91.6 (18.3)	*0.19*	-1.64 (-4.07, 0.8)	*0.187*	-1.94 (-4.43, 0.54)	*0.125*
Information during visit has been adequate (yes, %)	907	358 (81.2%)	370 (79.4%)	*0.50*	0.89 (0.64, 1.24)	*0.501*	0.89 (0.64, 1.25)	*0.501*
Patient did not understand something during the round (yes, %)	908	54 (12.2%)	49 (10.5%)	*0.41*	0.84 (0.56, 1.27)	*0.406*	0.91 (0.6, 1.39)	*0.679*
Estimation of my participation in the discussion (VAS 0-100), mean (SD)	837	63.8 (38.0)	59.3 (32.2)	*0.064*	-4.51 (-9.28, 0.27)	*0.064*	-4.67 (-9.5, 0.16)	*0.058*
Disease was explained in an understandable way (VAS 0-100), mean (SD)	720	84.3 (27.0)	84.7 (26.3)	*0.84*	0.4 (-3.5, 4.3)	*0.841*	0.16 (-3.81, 4.12)	*0.939*
Treatment was explained in an understandable way (VAS 0-100), mean (SD)	707	82.1 (28.5)	80.5 (30.2)	*0.48*	-1.56 (-5.9, 2.78)	*0.48*	-1.61 (-6.04, 2.81)	*0.474*
Upcoming examinations were explained in an understandable way (VAS 0-100), mean (SD)	635	82.6 (27.9)	83.5 (28.2)	*0.71*	0.84 (-3.53, 5.22)	*0.705*	0.96 (-3.51, 5.43)	*0.673*
The information given during the visit was clear and understandable (VAS 0-100), mean (SD)	859	92.1 (15.9)	90.3 (19.6)	*0.15*	-1.76 (-4.16, 0.63)	*0.149*	-1.78 (-4.23, 0.67)	*0.154*
**Patient perception regarding discomfort during the ward round**								
Medical terms used during ward round were confusing (VAS 0-100), mean (SD)	853	15.9 (28.2)	19.2 (30.4)	*0.099*	3.32 (-0.63, 7.27)	*0.099*	3.89 (-0.07, 7.85)	*0.054*
Ward round discussions made me worry (VAS 0-100), mean (SD)	871	6.6 (25.7)	11.7 (24.8)	***0.003***	5.13 (1.78, 8.49)	***0.003***	4.82 (1.4, 8.24)	***0.006***
I felt uncomfortable during ward round (VAS 0-100), mean (SD)	870	4.5 (15.6)	8.2 (21.6)	***0.005***	3.61 (1.1, 6.13)	***0.005***	3.2 (0.64, 5.76)	***0.014***
Ward round discussions unsettled me (VAS 0-100), mean (SD)	752	4.4 (15.9)	7.2 (19.9)	***0.033***	2.81 (0.23, 5.4)	***0.033***	2.84 (0.22, 5.45)	***0.034***
**Patient perception regarding physician`s behaviour during the ward round**								

Physicians treated me with respect (VAS 0-100), mean (SD)	867	97.4 (8.8)	95.5 (13.1)	***0.011***	-1.94 (-3.43, -0.45)	***0.011***	-2.05 (-3.57, -0.53)	***0.008***
I was taken seriously (VAS 0-100), mean (SD)	861	96.1 (10.9)	94.0 (15.6)	***0.021***	-2.13 (-3.94, -0.32)	***0.021***	-2.17 (-4.01, -0.32)	***0.021***
Physicians respected my privacy (VAS 0-100), mean (SD)	794	89.7 (24.8)	91.1 (20.9)	*0.38*	1.42 (-1.78, 4.61)	*0.384*	1.41 (-1.85, 4.66)	*0.396*
Physicians showed compassion (VAS 0-100), mean (SD)	693	81.0 (28.4)	75.2 (33.5)	***0.014***	-5.86 (-10.53, -1.2)	***0.014***	-5.56 (-10.33, -0.8)	***0.022***
My issues were dealt with discreetly (VAS 0-100), mean (SD)	764	88.3 (23.7)	89.2 (21.1)	*0.55*	0.97 (-2.21, 4.16)	*0.549*	1.33 (-1.91, 4.57)	*0.42*
Some topics during ward round communication caused inconvenience (VAS 0-100), mean (SD)	853	4.0 (15.4)	7.6 (21.8)	***0.005***	3.61 (1.07, 6.15)	***0.005***	3.58 (0.99, 6.17)	***0.007***
I was encouraged to address personal topics (VAS 0-100), mean (SD)	624	85.5 (30.5)	84.4 (30.2)	*0.65*	-1.11 (-5.88, 3.66)	*0.648*	-1.41 (-6.28, 3.46)	*0.57*
My privacy was violated (VAS 0-100), mean (SD)	831	4.2 (16.2)	3.3 (13.3)	*0.38*	-0.9 (-2.91, 1.12)	*0.383*	-1.17 (-3.23, 0.88)	*0.264*
Teaching took place during ward round (yes, %)	909	136 (30.8%)	126 (26.9%)	*0.19*	0.83 (0.62, 1.1)	*0.193*	0.96 (0.71, 1.3)	*0.812*
- if yes , teaching was perceived as disruptive, (VAS 0-100) mean (SD)	257	1.7 (8.4)	5.7 (21.9)	*0.051*	4.01 (-0.02, 8.05)	***0.05***	4.14 (0.08, 8.2)	***0.046***
**Patients' perception regarding quality of care (VAS 0-100)**								
I felt "in good hands" in this hospital, mean (SD)	883	90.6 (15.1)	89.4 (15.6)	*0.27*	-1.15 (-3.18, 0.88)	*0.266*	-0.68 (-2.75, 1.38)	*0.516*
I felt there were contradicting statements from physicians and nursing team , mean (SD)	843	11.6 (24.8)	13.5 (26.6)	*0.27*	1.95 (-1.53, 5.43)	*0.272*	1.55 (-2, 5.1)	*0.392*
I feel confident with the physician team, mean (SD)	873	91.9 (14.4)	89.5 (16.8)	***0.025***	-2.39 (-4.47, -0.31)	***0.025***	-2.52 (-4.65, -0.4)	***0.02***
I feel confident with the nursing team, mean (SD)	876	93.2 (12.6)	91.7 (13.7)	*0.093*	-1.49 (-3.24, 0.25)	*0.093*	-1.7 (-3.48, 0.08)	*0.061*
There is good collaboration of physicians and nurses, mean (SD)	809	92.3 (12.4)	89.6 (14.9)	***0.007***	-2.63 (-4.54, -0.73)	***0.007***	-2.95 (-4.89, -1.01)	***0.003***
I feel physicians have high competence to treat the current illness , mean (SD)	834	93.3 (43.0)	89.4 (16.9)	*0.081*	-3.93 (-8.34, 0.48)	*0.081*	-3.66 (-8.16, 0.84)	*0.111*
I feel nurses have high competence to treat the current illness, mean (SD)	845	92.7 (11.5)	91.2 (25.9)	*0.28*	-1.5 (-4.21, 1.21)	*0.279*	-1.62 (-4.38, 1.15)	*0.251*

**adjusted for study centre, intervention. All differences calculated with linear regression models for continuous data. Odds ratios were calculated with logistic regression models. Abbreviations: OR, odds ratio; SD, standard deviation; CI, confidence interval; VAS, visual analogue scale; n, number*

Supplement 4. Risk factors for low satisfaction among patients with sensitive topics

	n	high Satisfaction	low Satisfaction	*p-value*	Difference or OR (95%CI)	*p-value*	Adjusted difference or OR (95%CI) *adjusted for centrum, intervention*	*p-value*
		212	254					
**WEMS-Technique communication**	466							
Waiting, n (%)		24 (11.3%)	30 (11.8%)	*0.87*	1.05 (0.59, 1.86)	*0.869*	*1.09 (0.61, 1.96)*	*0.766*
Echoing, n (%)		43 (20.3%)	64 (25.2%)	*0.21*	1.32 (0.85, 2.05)	*0.21*	*1.47 (0.92, 2.33)*	*0.107*
Mirroring, n (%)		14 (6.6%)	26 (10.2%)	*0.16*	1.61 (0.82, 3.17)	*0.166*	*1.75 (0.87, 3.51)*	*0.115*
Summarising, n (%)		18 (8.5%)	21 (8.3%)	*0.93*	0.97 (0.5, 1.88)	*0.931*	*1.01 (0.52, 1.96)*	*0.982*
Open-ended questions, n (%)		158 (74.5%)	188 (74.0%)	*0.90*	0.97 (0.64, 1.48)	*0.9*	*1 (0.65, 1.52)*	*0.982*
Confirming, n (%)		90 (42.5%)	102 (40.2%)	*0.62*	0.91 (0.63, 1.32)	*0.616*	*0.92 (0.63, 1.33)*	*0.64*
Nodding, n (%)		160 (75.5%)	197 (77.6%)	*0.60*	1.12 (0.73, 1.73)	*0.596*	*1.16 (0.75, 1.79)*	*0.505*
**NURSE-Technique communication (in patients with emotions)**		34	46					
Naming (of emotion), n (%)	78	8 (24%)	7 (15%)	*0.35*	0.58 (0.19, 1.8)	*0.35*	*0.46 (0.14, 1.47)*	*0.189*
Understanding (verbal/nonverbal), n (%)	78	8 (24%)	15 (33%)	*0.38*	1.57 (0.58, 4.29)	*0.377*	*1.59 (0.55, 4.61)*	*0.395*
Respecting, n (%)	78	13 (38%)	12 (26%)	*0.25*	0.57 (0.22, 1.48)	*0.249*	*0.37 (0.13, 1.09)*	*0.07*
Supporting, n (%)	78	13 (38%)	12 (26%)	*0.25*	0.57 (0.22, 1.48)	*0.249*	*0.54 (0.2, 1.48)*	*0.232*
Exploring, n (%)	78	13 (38%)	12 (26%)	*0.25*	0.57 (0.22, 1.48)	*0.249*	*0.54 (0.2, 1.48)*	*0.232*
Hasty solcae, n (%)	78	1 (3%)	2 (4%)	*0.74*	1.5 (0.13, 17.25)	*0.745*	*1.54 (0.13, 19.05)*	*0.735*
Distracting/Ignoring, n (%)	78	1 (3%)	2 (4%)	*0.74*	1.5 (0.13, 17.25)	*0.745*	*1.54 (0.13, 19.05)*	*0.735*
Dismissing (“that’s not so bad”), n (%)	78	2 (6%)	2 (4%)	*0.76*	0.73 (0.1, 5.44)	*0.756*	*0.61 (0.08, 4.69)*	*0.636*
Replies with providing information instead of addressing emotion, n (%)	78	16 (47%)	20 (43%)	*0.75*	0.87 (0.36, 2.11)	*0.75*	*0.88 (0.35, 2.23)*	*0.793*
Physician talks about emotion, n (%)	78	6 (18%)	3 (7%)	*0.12*	0.33 (0.08, 1.41)	*0.133*	*0.35 (0.07, 1.67)*	*0.188*
Overall rating: Responding to emotions, n (%)	77	13 (38%)	14 (31%)	*0.51*	0.73 (0.29, 1.86)	*0.509*	*0.98 (0.36, 2.67)*	*0.969*
**Other communication factors**	466							
Medical staff talks to patient about diagnosis, n (%)		117 (55.2%)	149 (58.7%)	*0.45*	1.15 (0.8, 1.67)	*0.451*	*1.19 (0.82, 1.73)*	*0.368*
Medical staff talks to patient about symptoms, n (%)		198 (93.4%)	237 (93.3%)	*0.97*	0.99 (0.47, 2.05)	*0.969*	*1.02 (0.49, 2.12)*	*0.963*
Medical staff talks to patient about treatment, n (%)		167 (78.8%)	209 (82.3%)	*0.34*	1.25 (0.79, 1.98)	*0.34*	*1.23 (0.78, 1.96)*	*0.375*
Medical staff talks to patient about next steps, n (%)		190 (89.6%)	234 (92.1%)	*0.35*	1.35 (0.72, 2.56)	*0.349*	*1.33 (0.71, 2.52)*	*0.376*

Medical staff talks to patient about social issues, n (%)		151 (71.2%)	164 (64.6%)	0.13	0.74 (0.5, 1.09)	0.127	0.75 (0.5, 1.12)	0.163
Medical staff talks about instead of with the patient after case discussion, n (%)		157 (74.1%)	189 (74.4%)	0.93	1.02 (0.67, 1.55)	0.931	1.07 (0.7, 1.65)	0.748
Physician and patient talk talk at cross purposes, n (%)		17 (8.0%)	16 (6.3%)	0.47	0.77 (0.38, 1.57)	0.472	0.78 (0.38, 1.58)	0.486
Patient does not seem to understand something, but does not ask, n (%)		9 (4.2%)	8 (3.1%)	0.53	0.73 (0.28, 1.94)	0.531	0.78 (0.29, 2.11)	0.624
Physician disagrees with/corrects patient, n (%)		14 (6.6%)	41 (16.1%)	**0.001**	2.72 (1.44, 5.15)	**0.002**	2.78 (1.47, 5.27)	**0.002**
Patient disagrees with/corrects physician, n (%)		14 (6.6%)	37 (14.6%)	**0.006**	2.41 (1.27, 4.59)	**0.007**	2.42 (1.27, 4.61)	**0.007**
Occurrence of cue, n (%)		28 (13.2%)	49 (19.3%)	0.078	1.57 (0.95, 2.6)	0.08	1.55 (0.93, 2.57)	0.093
Addressing cue concern, n (%)		17 (8.0%)	22 (8.7%)	0.80	1.09 (0.56, 2.11)	0.803	1.05 (0.54, 2.04)	0.888
Replies with providing information instead of exploration, n (%)		10 (4.7%)	22 (8.7%)	0.094	1.92 (0.89, 4.14)	0.098	1.91 (0.88, 4.14)	0.1
**Patient knowledge about medical care**	466							
Average subjective knowledge about their medical care, mean (SD)		87.0 (16.2)	70.7 (22.9)	**<0.001**	0.96 (0.94, 0.97)	**<0.001**	0.95 (0.94, 0.97)	**<0.001**
Subjective understanding of disease, mean (SD)		88.48 (16.27)	74.14 (23.03)	**<0.001**	0.96 (0.95, 0.97)	**<0.001**	0.96 (0.95, 0.97)	**<0.001**
Subjective understanding of therapeutic approach, mean (SD)		85.18 (23.36)	69.55 (27.99)	**<0.001**	0.98 (0.97, 0.98)	**<0.001**	0.97 (0.96, 0.98)	**<0.001**
Subjective understanding of further plans for care, mean (SD)		87.14 (22.39)	68.21 (27.81)	**<0.001**	0.97 (0.96, 0.98)	**<0.001**	0.97 (0.96, 0.98)	**<0.001**
Average objective knowledge about their medical care, mean (SD)		71.7 (25.3)	66.2 (26.0)	**0.023**	0.99 (0.98, 1)	**0.024**	0.99 (0.98, 1)	**0.027**
Objective understanding of disease, mean (SD)		68.42 (33.64)	64.5 (33.26)	0.21	1 (0.99, 1)	0.207	1 (0.99, 1)	0.229
Objective understanding of therapeutic approach, mean (SD)		72.52 (33.61)	65.35 (35.44)	**0.026**	0.99 (0.99, 1)	**0.027**	0.99 (0.99, 1)	**0.026**
Objective understanding of further plans for care, mean (SD)		74.01 (33.45)	68.8 (34.46)	0.10	1 (0.99, 1)	0.101	1 (0.99, 1)	0.112
**Measured timeliness of ward round**	466							
Duration of outside the room discussions (min), mean (SD)		4.6 (4.8)	5.2 (5.0)	0.22	1.02 (0.99, 1.06)	0.215	1.05 (0.99, 1.1)	0.095
Duration of bedside discussions (min), mean (SD)		8.7 (5.0)	9.4 (5.0)	0.18	1.03 (0.99, 1.06)	0.179	1.04 (1, 1.09)	0.078
Duration of debriefing outside the room (min), mean (SD)		.53 (1.1)	.57 (1.2)	0.81	1.02 (0.87, 1.2)	0.807	1.03 (0.87, 1.21)	0.739
Total duration of ward round per patient (min), mean (SD)		13.9 (5.6)	15.1 (5.6)	**0.019**	1.04 (1.01, 1.08)	**0.02**	1.05 (1.01, 1.08)	**0.01**
**Patient perception regarding time spent on ward round**	466							
Overall duration of ward round was sufficient (VAS 0-100), mean (SD)		95.78 (15.38)	83.81 (24.55)	**<0.001**	0.97 (0.95, 0.98)	**<0.001**	0.96 (0.95, 0.98)	**<0.001**
Time spent with physicians was sufficient (VAS 0-100), mean (SD)		96.71 (10.02)	80.66 (22.53)	**<0.001**	0.92 (0.9, 0.94)	**<0.001**	0.92 (0.9, 0.94)	**<0.001**
Patient estimation of time spent with patient on ward round (min), mean (SD)		12.18 (7.18)	11.26 (6.58)	0.15	0.98 (0.96, 1.01)	0.154	0.98 (0.96, 1.01)	0.176
Patient estimation of time spent per day with patient case overall (min), mean (SD)		82.09 (86.72)	66.73 (66.44)	**0.031**	1 (0.99, 1)	**0.036**	1 (0.99, 1)	**0.035**
The ward round was helpful for better								
- understanding the main illness (VAS 0-100), mean (SD)		75.03 (32.91)	54.64 (33.95)	**<0.001**	0.98 (0.98, 0.99)	**<0.001**	0.98 (0.98, 0.99)	**<0.001**
- further therapeutic measures (VAS 0-100), mean (SD)		74.14 (32.45)	53.03 (33.52)	**<0.001**	0.98 (0.97, 0.99)	**<0.001**	0.98 (0.97, 0.99)	**<0.001**
- further plans of care (VAS 0-100), mean (SD)		83.33 (69.99)	62.12 (52.73)	**<0.001**	0.99 (0.98, 0.99)	**<0.001**	0.99 (0.98, 0.99)	**<0.001**
All my questions were answered (VAS 0-100), mean (SD)		97.05 (8.55)	82.10 (24.16)	**<0.001**	0.92 (0.9, 0.94)	**<0.001**	0.92 (0.9, 0.94)	**<0.001**

I was able to understand all answers to my questions (VAS 0-100), mean (SD)		96.84 (10.74)	85.85 (20.14)	***<0.001***	0.93 (0.91, 0.95)	***<0.001***	0.93 (0.91, 0.95)	***<0.001***
Information during visit has been adequate (yes, %)		197 (92.9%)	176 (69.3%)	***<0.001***	0.17 (0.1, 0.31)	***<0.001***	0.17 (0.1, 0.31)	***<0.001***
Patient did not understand something during the round (yes, %)		13 (6.1%)	36 (14.2%)	***0.005***	2.53 (1.3, 4.9)	***0.006***	2.58 (1.32, 5.02)	***0.005***
Estimation of my participation in the discussion (VAS 0-100), mean (SD)		67.80 (30.61)	51.78 (29.76)	***<0.001***	0.98 (0.98, 0.99)	***<0.001***	0.98 (0.98, 0.99)	***<0.001***
Disease was explained in an understandable way (VAS 0-100), mean (SD)		91.95 (19.46)	76.61 (27.1)	***<0.001***	0.97 (0.96, 0.98)	***<0.001***	0.97 (0.96, 0.98)	***<0.001***
Treatment was explained in an understandable way (VAS 0-100), mean (SD)		91.32 (20.51)	69.49 (31.19)	***<0.001***	0.96 (0.95, 0.97)	***<0.001***	0.96 (0.95, 0.97)	***<0.001***
Upcoming examinations were explained in an understandable way (VAS 0-100), mean (SD)		91.64 (19.12)	74.34 (28.26)	***<0.001***	0.96 (0.95, 0.98)	***<0.001***	0.96 (0.95, 0.97)	***<0.001***
The information given during the visit was clear and understandable (VAS 0-100), mean (SD)		97.86 (9.04)	82.92 (22.92)	***<0.001***	0.9 (0.88, 0.93)	***<0.001***	0.9 (0.88, 0.93)	***<0.001***
**Patient perception regarding discomfort during the ward round**	466							
Medical terms used during ward round were confusing (VAS 0-100), mean (SD)		14.1 (27.87)	23.13 (30.42)	***0.003***	1.01 (1, 1.02)	***0.003***	1.01 (1, 1.02)	***0.003***
Ward round discussions made me worry (VAS 0-100), mean (SD)		7.93 (22.02)	15.86 (25.73)	***<0.001***	1.01 (1.01, 1.02)	***0.001***	1.02 (1.01, 1.02)	***0.001***
I felt uncomfortable during ward round (VAS 0-100), mean (SD)		3.8 (14.88)	11.98 (24.44)	***<0.001***	1.02 (1.01, 1.04)	***<0.001***	1.02 (1.01, 1.04)	***<0.001***
Ward round discussions unsettled me (VAS 0-100), mean (SD)		2.23 (9.31)	12.01 (22.3)	***<0.001***	1.06 (1.03, 1.08)	***<0.001***	1.06 (1.03, 1.08)	***<0.001***
**Patient perception regarding physician`s behaviour during the ward round**								
Physicians treated me with respect (VAS 0-100), mean (SD)	466	99.31 (3.02)	91.87 (16.37)	***<0.001***	0.85 (0.8, 0.9)	***<0.001***	0.85 (0.8, 0.9)	***<0.001***
I was taken seriously (VAS 0-100), mean (SD)	466	98.82 (4.97)	88.87 (19.54)	***<0.001***	0.89 (0.85, 0.92)	***<0.001***	0.89 (0.85, 0.92)	***<0.001***
Physicians respected my privacy (VAS 0-100), mean (SD)	466	95.83 (14.64)	86.64 (21.55)	***<0.001***	0.96 (0.94, 0.98)	***<0.001***	0.96 (0.94, 0.98)	***<0.001***
Physicians showed compassion (VAS 0-100), mean (SD)	466	83.98 (27.57)	66.79 (30.85)	***<0.001***	0.98 (0.97, 0.99)	***<0.001***	0.98 (0.97, 0.99)	***<0.001***
My issues were dealt with discreetly (VAS 0-100), mean (SD)	466	95.01 (14.75)	83.95 (21.16)	***<0.001***	0.95 (0.94, 0.97)	***<0.001***	0.95 (0.94, 0.97)	***<0.001***
Some topics during ward round communication caused inconvenience (VAS 0-100), mean (SD)	466	3.66 (15.99)	10.88 (23.80)	***<0.001***	1.02 (1.01, 1.03)	***0.001***	1.02 (1.01, 1.03)	***0.001***
I was encouraged to address personal topics (VAS 0-100), mean (SD)	466	90.57 (21.35)	76.54 (27.65)	***<0.001***	0.97 (0.96, 0.98)	***<0.001***	0.97 (0.96, 0.98)	***<0.001***
My privacy was violated (VAS 0-100), mean (SD)	466	0.88 (5.30)	5.19 (15.55)	***<0.001***	1.07 (1.02, 1.12)	***0.003***	1.07 (1.02, 1.12)	***0.003***
Teaching took place during ward round (yes, %)	466	61 (28.8%)	65 (25.6%)	*0.44*	0.85 (0.57, 1.28)	*0.441*	0.87 (0.57, 1.33)	*0.51*
- if yes , teaching was perceived as disruptive, (VAS 0-100) mean (SD)	125	0.8 (6.4)	10.3 (29.3)	***0.015***	1.04 (0.99, 1.08)	*0.09*	1.04 (1, 1.08)	*0.07*
**Patients' perception regarding quality of care (VAS 0-100)**	466							
I felt "in good hands" in this hospital, mean (SD)		96.19 (9.28)	83.57 (17.04)	***<0.001***	0.91 (0.88, 0.93)	***<0.001***	0.9 (0.88, 0.93)	***<0.001***
I felt there were contradicting statements from physicians and nursing team, mean (SD)		7.58 (20.90)	18.87 (28.23)	***<0.001***	1.02 (1.01, 1.03)	***<0.001***	1.02 (1.01, 1.03)	***<0.001***
I feel Confident with the physician team, mean (SD)		95.88 (11.54)	83.79 (18.07)	***<0.001***	0.92 (0.9, 0.94)	***<0.001***	0.92 (0.9, 0.94)	***<0.001***
I feel Confident with the nursing team, mean (SD)		97.45 (6.46)	86.91 (15.65)	***<0.001***	0.89 (0.87, 0.92)	***<0.001***	0.89 (0.87, 0.92)	***<0.001***
There is good collaboration of physicians and nurses, mean (SD)		95.68 (9.14)	84.25 (15.8)	***<0.001***	0.91 (0.89, 0.93)	***<0.001***	0.91 (0.89, 0.93)	***<0.001***
I feel physicians have high competence to treat the current illness, mean (SD)		96.35 (9.71)	82.84 (18.49)	***<0.001***	0.9 (0.88, 0.93)	***<0.001***	0.9 (0.88, 0.93)	***<0.001***
I feel nurses have high competence to treat the current illness, mean (SD)		96.51 (9.16)	86.39 (31.79)	***<0.001***	0.92 (0.9, 0.94)	***<0.001***	0.93 (0.91, 0.95)	***<0.001***

**adjusted for study centre, intervention. All differences calculated with linear regression models for continuous data. Odds ratios were calculated with logistic regression models. Abbreviations: OR, odds ratio; SD, standard deviation; CI, confidence interval; VAS, visual analogue scale; n, number*

Supplement 5. Examples of discussions addressing delicate topics

Number of Example	Sensitive Topic	Predictor	Transcript	Current Location	Intervention group
1	Medical ambiguity	Physician disagrees with/corrects patient	Chief physician (CP): We know that you normally take Diazepam, which has a long half-life… Patient (P): Yes, 24 hours. Chief physician: Even longer, especially if the liver is not working properly. I've seen two people die from Diazepam. We prefer to prescribe Lorazepam Patient: I do not tolerate Lorazepam. Chief physician: What do you mean by “don't tolerate”? Patient: Once when I withdrew from alcohol and doctors gave me Lorazepam I hallucinated. I saw a mouse in my room. Then I had a delirium and woke up in another hospital. Chief physician: We cannot assume that this was because of the Lorazepam. It was more likely due to the alcohol withdrawal. Patient: Yes, but I've never experienced anything like that with Diazepam. Chief physician: It is important to me that we informed you about this…	bedside	outside
2	Medical ambiguity	Occurrence of cue	CP: So far, we have tried many different medications, but we cannot solve the issue with drugs alone. In my opinion an intervention is necessary, maybe even two. The goal is to improve the function of your heart and thereby improve your breathing. P: And this intervention is nothing I need to worry about? CP: Well, an intervention is always an intervention. It's never no risk, right? But it is a low risk I have to say.	bedside	outside
3	Psychiatric comorbidities	Physician disagrees with/corrects patient	CP: The functioning of your liver is very reduced. After all, it was poisoned by the alcohol, and now you have a cirrhosis. P: A cirrhosis already? CP: Certainly, an early stage of a cirrhosis, yes. If someone does drink such a high amount over such a long time… P: I did drink for 4 weeks only. CP: Yes, but you have a history of 26 years of recurrent relapses. P: Yes, but you did an ultrasound last time and I was told everything was fine? CP: Well, everything fine would be an exaggeration. A photo of someone only gives limited information about her or his character. It's the same with the liver. P: Okay…	bedside	outside
4	Tumour diagnosis	Distracting/Ignoring emotion	CP: Did anyone close to you have had stomach problems? P: Yes, my siblings have. They had cancer as well. And my husband, of course. CP: Mhm, I am sorry about this. P: Yeah, well no one can do anything about it. I'll have to deal with it by myself, right? Because it still hurts. It was not long ago. Nurse (N): How long? P: 4 years. CA: What about diarrhoea? Anyone close to you suffered from diarrhoea? P: I don't know.	bedside	bedside
5	Social issues (e.g. need for nursing home)	NURSE-Technique, Understanding	Patient: I planned to move in with my partner after my hospital stay. She's a former nurse, but now a relative of hers has died. Chief physician: Oh dear. Patient (tearful): When she heard what could happen to me, she said no. I mean, over the last years I've burdened her with my illness. But now I am really down. Chief physician: Yes, that is… I can understand that. Patient: Now I had to agree with her and promised to look for assisted accommodation. Chief physician: Yes, we can discuss this with our case management team. Or have you already found something? Patient: No, because I expected to move in with her. But now her relative has passed away and it is all so complicated… Chief physician: Yeah, I understand! But unfortunately, we don't have much time during the ward round so	bedside	outside

			we'll have to discuss your further care on another occasion.<br>Patient: Okay, yes, thank you.		
6	Non-adherence	Medical staff talks to patient about treatment	CP: Your gastrointestinal tract is attacked by your immune system due to the transplant (GvHD). You will receive some medication against it. However, treatment is kind of tricky as you must take that medication very carefully. To treat this, you need to be more compliant with the medication.<br>P: I did so.<br>CP: Sure…<br>P: We already talked about that last time. I took my medication but at times that were more comfortable for me.<br>CP: This is okay, as long as the time interval between the intake of the Cyclosporine remains the same.<br>P: Yeah, I tried…<br>CP: So, you are aware, that this is important?<br>P: Yes, I am.	bedside	bedside
