# Supplementary material for: Occurrence of sensitive topics during ward round: an ancillary analysis of the BEDSIDE-OUTSIDE trial
Source: BMJ Open. 2023 Sep 21;13(9):e073584. doi: 10.1136/bmjopen-2023-073584 (PMC10514661; doi:10.1136/bmjopen-2023-073584)
Supplement: Author's manuscript [file bmjopen-2023-073584.draft_revisions.pdf]

BMJ Open

BMJ Open is committed to open peer review. As part of this commitment we make the peer review history of every article we publish publicly available.

When an article is published we post the peer reviewers' comments and the authors' responses online. We also post the versions of the paper that were used during peer review. These are the versions that the peer review comments apply to.

The versions of the paper that follow are the versions that were submitted during the peer review process. They are not the versions of record or the final published versions. They should not be cited or distributed as the published version of this manuscript.

BMJ Open is an open access journal and the full, final, typeset and author-corrected version of record of the manuscript is available on our site with no access controls, subscription charges or pay-per-view fees (http://bmjopen.bmj.com).

If you have any questions on BMJ Open's open peer review process please email info.bmjopen@bmj.com

BMJ Open

# The occurrence of sensitive topics during ward round: An ancillary analysis of the BEDSIDE-OUTSIDE Trial



| | |
|---|---|
| Journal: | *BMJ Open* |
| Manuscript ID | bmjopen-2023-073584 |
| Article Type: | Original research |
| Date Submitted by the Author: | 15-Mar-2023 |





| | |
|---|---|
| Complete List of Authors: | Gross, Sebastian; University Hospital Basel, Medical Communication / Psychosomatic Medicine<br>Becker, Christoph; University Hospital Basel, Medical Communication / Psychosomatic Medicine<br>Beck, Katharina; University Hospital Basel, Medical Communication / Psychosomatic Medicine<br>Memma, Valentina; University Hospital Basel, Medical Communication / Psychosomatic Medicine<br>Gaab, Jens; University of Basel<br>Schütz, Philipp; Kantonsspital Aarau, Endocrinology, Diabetes and Metabolism<br>Leuppi, Jörg; Kantonsspital Baselland Standort Liestal, University Clinic of Medicine<br>Schaefert, Rainer; Universitatsspital Basel, Psychosomatics<br>Langewitz, Wolf Axel ; University Hospital Basle, Psychosomatic Medicine<br>Trendelenburg, Marten; University of Basel<br>Breidthardt, Tobias; Innere Medizin Universitatsspital Basel, Internal Medicine<br>Eckstein, J; Basel University Hospital<br>Osthoff, Michael; Univ Basel Hosp, Internal Medicine<br>Bassetti, Stefano; University Hospital Basel, Division of Internal Medicine<br>Hunziker, Sabina; University Hospital Basel, Medical Communication / Psychosomatic Medicine |





| | |
|---|---|
| Keywords: | Hospitalization, MEDICAL ETHICS, MEDICAL EDUCATION & TRAINING, INTERNAL MEDICINE |
| | |



SCHOLARONE™
Manuscripts

*I, the Submitting Author has the right to grant and does grant on behalf of all authors of the Work (as defined in the below author licence), an exclusive licence and/or a non-exclusive licence for contributions from authors who are: i) UK Crown employees; ii) where BMJ has agreed a CC-BY licence shall apply, and/or iii) in accordance with the terms applicable for US Federal Government officers or employees acting as part of their official duties; on a worldwide, perpetual, irrevocable, royalty-free basis to BMJ Publishing Group Ltd ("BMJ") its licensees and where the relevant Journal is co-owned by BMJ to the co-owners of the Journal, to publish the Work in this journal and any other BMJ products and to exploit all rights, as set out in our licence.*

*The Submitting Author accepts and understands that any supply made under these terms is made by BMJ to the Submitting Author unless you are acting as an employee on behalf of your employer or a postgraduate student of an affiliated institution which is paying any applicable article publishing charge ("APC") for Open Access articles. Where the Submitting Author wishes to make the Work available on an Open Access basis (and intends to pay the relevant APC), the terms of reuse of such Open Access shall be governed by a Creative Commons licence – details of these licences and which Creative Commons licence will apply to this Work are set out in our licence referred to above.*

*Other than as permitted in any relevant BMJ Author's Self Archiving Policies, I confirm this Work has not been accepted for publication elsewhere, is not being considered for publication elsewhere and does not duplicate material already published. I confirm all authors consent to publication of this Work and authorise the granting of this licence.*

For peer review only

# The occurrence of sensitive topics during ward round: An ancillary analysis of the BEDSIDE-OUTSIDE Trial

Sebastian Gross, MSc[1], Christoph Becker, MD[1,2], Katharina Beck, Ph.D.[1], Valentina Memma, MSc[1], Jens Gaab, Ph.D.[4], Philipp Schuetz, MD[3,5], Joerg D. Leuppi, MD[3,6], Rainer Schaefert, MD[1,3,8], Wolf Langewitz, MD[1,3], Marten Trendelenburg, MD[3,7], Tobias Breidthardt, MD[3,7], Jens Eckstein, MD[3,7], Michael Osthoff, MD[3,7], Stefano Bassetti, MD[3,7] and Sabina Hunziker, MD[1,7]

[1]Medical Communication, Department of Psychosomatic Medicine, University Hospital Basel, Basel, Switzerland

[2]Emergency Department, University Hospital Basel, Basel, Switzerland

[3]Faculty of Medicine, University of Basel, Switzerland

[4] Division of Clinical Psychology and Psychotherapy, Faculty of Psychology, University of Basel, Basel, Switzerland

[5]Division of Internal Medicine, Kantonsspital Aarau, Aarau, Switzerland

[6]University Center of Internal Medicine, Kantonsspital Baselland, Liestal, Switzerland

[7]Division of Internal Medicine, University Hospital Basel, Basel, Switzerland

[8]Department of Psychosomatics and Psychiatry, Bethesda Hospital, Basel, Switzerland

**Corresponding author:**

Prof. Dr. med. Sabina Hunziker, MD, MPH



For peer review only - http://bmjopen.bmj.com/site/about/guidelines.xhtml

University Hospital Basel

Petersgraben 4

CH - 4031 Basel

Switzerland

Email: sabina.hunziker@usb.ch

**Word count**

**Abstract: 283**

**Main text: 3323**

For peer review only

## Abstract

**Objective:** Discussing sensitive topics (e.g., medical uncertainty, social issues, non-adherence) during ward rounds is challenging and may negatively impact patient satisfaction with the health care they are receiving. In the previous multicentre randomised BEDSIDE-OUTSIDE trial focusing on communication during ward rounds, we investigated the interplay between sensitive topics and low reported satisfaction with care.

**Design:** Secondary analysis of a randomised controlled trial

**Setting**: Three Swiss teaching hospitals

**Participants**: Adult patients hospitalized for medical care

**Interventions:** We analysed predefined sensitive health topics and specific elements of communication from audiotapes recorded during ward rounds, for both patients dealing with and without sensitive topics.

**Primary and secondary outcome measures:** The primary endpoint was overall patient satisfaction with care; measured on a visual analogue scale from 0 to 100. Secondary endpoints included duration of ward rounds and further satisfaction outcomes.

**Results:** Of the 919 included patients, 474 had at least one sensitive topic including medical uncertainty (n=251), mental comorbidities (n=137), tumour diagnosis (n=137), and social issues (n= 125). Compared to patients without sensitive topics, patients with sensitive topics reported lower satisfaction with care (mean (SD), 87.7 [±14.6] versus 90.2 [±12.1], adjusted difference -2.5 [95%CI -4.28 to -0.72], p=0.006. Among patients with sensitive topics, risk factors for low satisfaction included several parameters concerning patient-physician interaction such as disagreements during




For peer review only - http://bmjopen.bmj.com/site/about/guidelines.xhtml

For peer review only

ward rounds (mean (SD), 14/212 [6.6%] versus 41/254 [16.1%], adjusted OR 2.78 [95%CI 1.47 to 5.27], p=0.002).

**Conclusions**: A large proportion of medical inpatients must deal with sensitive health topics. This is associated with lower satisfaction with care, particularly if the patient perceives the interaction with doctors during ward rounds as unsatisfactory. Educating physicians on specific communication techniques may help improve care for these patients.

**Trial registration**: clinicaltrials.gov; NCT03210987

## Strengths and limitations

- To our knowledge, this is the first trial to analyse communication regarding sensitive topics during ward rounds.
- We investigated a large sample of a broad general medicine population from multiple centres.
- Only Swiss teaching hospitals were included, which might cause selection bias and limit the generalisability of the findings.
- Ward rounds of clinical practice procedures in the three participating hospitals were not standardised to ensure external validity – which may therefore reduce the internal validity.
- The questionnaire used to assess patients' perceptions has not yet been validated.

For peer review only



For peer review only - http://bmjopen.bmj.com/site/about/guidelines.xhtml

## Introduction

Life threatening disease, conflict with patients, patient non-adherence, psychiatric comorbidities, substance abuse, medical uncertainty, etc. are considered to be sensitive topics that so far have been mainly researched qualitatively.[1] The frequency of occurrence, and whether they are discussed in clinical practice remains unclear. Sensitive topics may be important to address as they could affect the patient's well-being and include relevant health care information. On the other hand, the sharing of personal and sensitive information during clinical ward rounds in a non-private setting could potentially offend patients who interpret this as a breach of doctor-patient confidentiality.[2] Physicians may avoid addressing sensitive topics e.g. by terminating problematic conversations, withholding frank responses, downplaying the patient's expressed emotions, or inadequately acknowledging the sentiment underlying the patient's statements.[3 4] A previous multicentre randomised trial including 919 internal medicine patients (BEDSIDE-OUTSIDE trial) aimed to compare ward round communication of patient cases discussed at the patient's bedside or outside the room.[4]. One major finding was that physicians avoided sensitive topics more often when conversations were held at the patients' bedside. Moreover, an ancillary analysis of the BEDSIDE-OUTSIDE trial focusing on staff satisfaction showed that physicians prefer to discuss sensitive topics outside the room than at the patient's bedside.[5] Despite concerns about addressing sensitive topics with patients, evidence in outpatient settings indicates that discussing these topics may be associated with high satisfaction, positive perceptions of health care, reduced worry, and increased patient participation in treatment decisions in the outpatient setting.[6 7]



For peer review only - http://bmjopen.bmj.com/site/about/guidelines.xhtml

The current study aimed to quantitatively and qualitatively analyse audio tapes from internal medicine ward rounds of patients included in the BEDSIDE-OUTSIDE trial and to determine if discussing sensitive topics in clinical practice is associated with patient satisfaction.[4]

## Material and methods

### Study setting

The current study is a pre-planned secondary analysis of the BEDSIDE-OUTSIDE trial[4] - a pragmatic, investigator-initiated, open-label, multicentre randomised trial conducted in the general medical divisions of three Swiss teaching hospitals (University Hospital Basel, Kantonsspital Aarau, and Kantonsspital Baselland) between July 2017 and October 2019. The trial was registered prior to initiation at clinicaltrials.gov on 7 July 2017 (https://clinicaltrials.gov/ct2/show/NCT03210987) and approved by the local Ethics Committee (Northwest and Central Switzerland, EKNZ, 2017-00991). This report adheres to the CONSORT guidelines[8].

This analysis studied potential risk factors associated with sensitive topics, the association of sensitive topics with different outcomes, and potential risk factors for low or high levels of patient satisfaction with care. We also provided a qualitative overview of sensitive topic discussions during ward rounds.

### Original study population

Newly admitted adult inpatients on medical wards expecting their first weekly ward round consultation were approached regarding inclusion. Only one patient per room



For peer review only - http://bmjopen.bmj.com/site/about/guidelines.xhtml

was eligible and we excluded individuals with cognitive or hearing impairment, those unable to understand the local language, and patients who had previously been included in the study. All provided written informed consent.

**Study design, randomisation, and intervention of the original study**

Patients were randomised to either the “bedside group” or the “outside the room group” in a 1:1 ratio. In line with current practice, ward rounds for both groups followed the standard practices of each participating hospital.

Details of the study intervention have been reported earlier.[4] In brief; for the *bedside* group, case presentations, discussions, and clinical examination took place at the bedside, in front of the patient. In the *outside the room* group, patient case presentations and discussions were primarily held in the hallway, resulting in a list of topics that the group of professionals then suggest to the patient to be discussed during the ward rond. Patients, study coordinators, and treating clinicians were not blinded to the allocation. However, study investigators involved in a patient’s outcome assessment were blinded to trial allocation.

**Data collection**

Data collection was conducted at different points in time. Baseline patient data was collected before the ward round. An observer from the research team was present to document timing (i.e., the duration of the ward round allocated per patient). All visits were recorded with an iPad with voice recording software and rated afterwards by the research team. This approach allowed for the coding of various predefined sensitive topics and elements of communication. After the ward rounds, a second blinded member of the research team interviewed participating patients using a standardised



For peer review only - http://bmjopen.bmj.com/site/about/guidelines.xhtml

questionnaire. All case report forms and items have been described in the original trial.[4]

**Patient Involvement**

A total of 25 patients hospitalised on the medical wards of the University Hospital of Basel, as well as 15 healthcare providers (physicians and nurses) with experience in daily medical practice and specially in medical ward rounds were involved in the design of the study and intervention of the main paper[4]. To design the trial, patients and healthcare providers helped us in prioritisation and selection of outcomes. Patients were asked for priority focus of this study. To design the intervention for the main trial, input was sought from patients and healthcare providers.

**Outcome measures of the ancillary analysis**

*Baseline factors and predictors*

We assessed patient baseline characteristics including age, sex, number of children, family status, citizenship, level of education, occupation, main diagnosis, and comorbidities. Predictors for the occurrence of sensitive topics included patients' quality of life assessed with the validated EuroQuol EQ-5D questionnaire[9], patient's Decisional Control Preference[10 11], application of specific patient-centred communication techniques (WEMS: waiting, echoing, mirroring, summarising; NURSE: naming, understanding, respecting, supporting and exploring [12 13], as well as the following general communication factors rated by the study team: information given regarding diagnosis, symptoms, treatment steps, social issues; talking "about" instead of "with" the patient; communicating at cross purposes. Furthermore, moments were identified, when raters had the impression that a patient did not



For peer review only - http://bmjopen.bmj.com/site/about/guidelines.xhtml

understand information but did not ask more questions; istances when the physician explicitly disagreed with or corrected a patient; when a patient disagreed with / corrected a physician; occurrence of Current Unvoiced Elements [*cue*s] and concerns; addressing *cue* and concerns; and replying by providing information instead of exploration. *Cues* and concerns were defined as proposed by previous research.[14] In short, a *cue* or concern included a verbally or non-verbally expressed hint expressed by the patient that might have a certain subjective importance and a negative emotional impact.

*Primary and secondary endpoints*

The primary endpoint for this analysis was patients' overall satisfaction, defined by the mean of several satisfaction measures (see secondary endpoints) and measured on a visual analogue scale (VAS) of 0-100, with 0 indicating the lowest and 100 the highest possible satisfaction. For the purpose of this study, patient satisfaction below the median were defined as low satisfaction. Secondary endpoints included satisfaction outcomes for: ward rounds, hospital stay, medical care, physician communication, and nursing team communication, measured on the same a visual analogue scale (VAS) from 0-100.

We also assessed three elements of patients' subjective and objective knowledge of their own medical situation: the understanding of the disease, therapeutic approach, and further plans for care. Each dimension was rated by the patient on a visual analogue scale from 0 to 100 (0 "no knowledge about the situation" to 100 "best possible knowledge about the situation"). Subjective knowledge was defined as patient's self-assessment of being informed and was rated during a structured interview in the afternoon after the ward round. The study team rated objective



For peer review only - http://bmjopen.bmj.com/site/about/guidelines.xhtml

knowledge by comparing patients' recall of information about their main disease with medical information from the chart.

Ward round duration was measured in minutes and included: total time during the ward round, individual outside the room discussions or bedside discussions, and debriefing outside the room.

Further secondary endpoints included multiple items relating to patients' perception of: time spent on the ward round, patients' discomfort during the ward round, physician behaviour during the ward round, and general quality of care (**Supplement Tables**). All were rated in the structured interview after the ward round and measured on a visual analogue scale from 0 to 100.

## Statistical analysis

Baseline parameters and outcomes were stratified among patients with and without sensitive topics. Logistic regression analyses were conducted to evaluate factors associated with sensitive topics. Furthermore, univariable linear and logistic regression models were conducted to investigate associations of sensitive topics with primary and secondary endpoints. We additionally calculated multivariable models adjusted for study centre and randomisation arm (bedside or hallway). In the subgroup of patients with sensitive topics, potential risk factors for low satisfaction compared to high satisfaction were assessed using Student's t-test. We used STATA 15.0 (Stata Corp., College Station, TX, USA) for all statistical analyses. A p-value of <0.05 (two-tailed) was considered statistically significant. STATA 15.0 (Stata Corp., College Station, TX, USA) was used for all analyses.



For peer review only - http://bmjopen.bmj.com/site/about/guidelines.xhtml

# Results

## Study flow of the original trial

Of 1441 patients approached for inclusion in the original trial, 1092 patients agreed and gave written informed consent. After exclusions, 919 patients were included in the final analysis.[4]

## Baseline characteristics (Table 1 and Supplement Table 1)

A total of 474 (51.6%) patients had at least one sensitive topic needing discussion during the ward round. In total, 791 sensitive topics emerged during ward round discussions, including: medical uncertainty (n = 251), psychiatric comorbidities (n = 161), tumour diagnosis (n = 137), social issues (n = 125), non-adherence (n = 43), interaction with the patient (n = 38), and treatment failure (n = 36) (**Figure 1**).



For peer review only - http://bmjopen.bmj.com/site/about/guidelines.xhtml

**Table 1. Associations of patient characteristics with sensitive topics**

| | n | All | No sensitive topics | Sensitive topics | *p-value* | OR (95%CI)* | *p-value* |
|---|---|---|---|---|---|---|---|
| **n** | | | 445 | 474 | | | |
| **Sociodemographic factors** | | | | | | | |
| Age, years; mean (SD) | 919 | 65.0 (15.9) | 65.7 (15.9) | 64.3 (15.9) | *0.16* | 1 (0.99, 1) | *0.304* |
| Female sex, n (%) | 919 | 361 (39.3%) | 173 (38.9%) | 188 (39.7%) | *0.81* | 1.05 (0.8, 1.37) | *0.741* |
| Number of children, mean (SD) | 919 | 2.5 (9.2) | 2.1 (6.7) | 2.8 (11.0) | *0.26* | 1.01 (0.99, 1.02) | *0.365* |
| **Family status** | 919 | | | | | | |
| Single, relationship, married, registered partnership, n (%) | | 648 (70.5%) | 317 (71.2%) | 331 (69.8%) | *0.84* | | |
| Separated, divorced, n (%) | | 147 (16.0%) | 68 (15.3%) | 79 (16.7%) | | 1.05 (0.73, 1.52) | *0.787* |
| Widowed, n (%) | | 124 (13.5%) | 60 (13.5%) | 64 (13.5%) | | 1.07 (0.72, 1.59) | *0.738* |
| **Citizenship** | 919 | | | | | | |
| Switzerland, n (%) | | 789 (85.9%) | 386 (86.7%) | 403 (85.0%) | *0.71* | | |
| Germany, n (%) | | 55 (6.0%) | 26 (5.8%) | 29 (6.1%) | | 0.97 (0.55, 1.68) | *0.904* |
| Other, n (%) | | 75 (8.2%) | 33 (7.4%) | 42 (8.9%) | | 1.22 (0.75, 1.98) | *0.421* |
| **Occupation** | 919 | | | | | | |
| Employed / working + IV, n (%) | | 259 (28.2%) | 129 (29.0%) | 130 (27.4%) | ***0.041*** | | |
| Unemployed / homemaker, n (%) | | 39 (4.2%) | 13 (2.9%) | 26 (5.5%) | | 2 (0.97, 4.09) | *0.059* |
| Retired / IV support, n (%) | | 605 (65.8%) | 296 (66.5%) | 309 (65.2%) | | 1.05 (0.78, 1.42) | *0.735* |
| In education, n (%) | | 6 (0.7%) | 5 (1.1%) | 1 (0.2%) | | 0.19 (0.02, 1.63) | *0.128* |
| Other, n (%) | | 10 (1.1%) | 2 (0.4%) | 8 (1.7%) | | 3.41 (0.7, 16.56) | *0.128* |
| **Comorbidities** | 919 | | | | | | |
| Charlson Comorbidity Index, mean (SD) | | 4.45 (2.88) | 4.4 (2.8) | 4.5 (3.0) | *0.54* | 0.76 (0.59, 1) | ***0.048*** |
| Cardiology, n (%) | | 485 (52.8%) | 248 (55.7%) | 237 (50.0%) | *0.082* | 0.86 (0.65, 1.13) | *0.273* |
| Neurology, n (%) | | 193 (21.0%) | 80 (18.0%) | 113 (23.8%) | ***0.029*** | 1.01 (0.77, 1.33) | *0.941* |
| Rheumatology/Immunology, n (%) | | 143 (15.6%) | 68 (15.3%) | 75 (15.8%) | *0.82* | 1.03 (0.78, 1.37) | *0.819* |
| Gastrointestinal, n (%) | | 254 (27.6%) | 118 (26.5%) | 136 (28.7%) | *0.46* | 1.18 (0.85, 1.65) | *0.325* |



For peer review only - http://bmjopen.bmj.com/site/about/guidelines.xhtml

| | | | | | | | |
|---|---|---|---|---|---|---|---|
| Endocrinology, n (%) | | 352 (38.3%) | 185 (41.6%) | 167 (35.2%) | ***0.048*** | 1.21 (0.86, 1.71) | *0.264* |
| Respiratory, n (%) | | 315 (34.3%) | 152 (34.2%) | 163 (34.4%) | *0.94* | 1.07 (0.81, 1.4) | *0.627* |
| Infectious diseases, n (%) | | 171 (18.6%) | 77 (17.3%) | 94 (19.8%) | *0.33* | 1.12 (0.85, 1.48) | *0.416* |
| Renal, n (%) | | 317 (34.5%) | 150 (33.7%) | 167 (35.2%) | *0.63* | 0.25 (0.07, 0.91) | ***0.035*** |
| Gynecology, n (%) | | 14 (1.5%) | 11 (2.5%) | 3 (0.6%) | ***0.023*** | 0.26 (0.07, 0.94) | ***0.04*** |
| Urology, n (%) | | 93 (10.1%) | 43 (9.7%) | 50 (10.5%) | *0.66* | 1.24 (0.8, 1.93) | *0.338* |
| Oncology, n (%) | | 287 (31.2%) | 145 (32.6%) | 142 (30.0%) | *0.39* | 1.81 (1.13, 2.9) | ***0.014*** |
| Psychiatry, n (%) | | 104 (11.3%) | 31 (7.0%) | 73 (15.4%) | ***<0.001*** | 1.97 (1.21, 3.2) | ***0.006*** |
| Depression, n (%) | | 82 (8.9%) | 29 (6.5%) | 53 (11.2%) | ***0.013*** | 0.89 (0.68, 1.15) | *0.364* |
| Other, n (%) | | 498 (54.2%) | 248 (55.7%) | 250 (52.7%) | *0.36* | 0.95 (0.73, 1.25) | *0.734* |
| Health self-rating VAS [0-100], mean (SD) | 856 | 56.9 (23.0) | 58.7 (22.5) | 55.1 (23.3) | ***0.021*** | 0.99 (0.99, 1) | ***0.018*** |
| Quality of life (EQ-5D) Index, mean (SD) | 889 | | 0.749 (0.278) | 0.671 (0.301) | ***<0.001*** | 0.42 (0.26, 0.67) | ***<0.001*** |

**adjusted for study centre, intervention. Odds ratios were calculated with logistic regression models. Abbreviations: OR, odds ratio; SD, standard deviation; CI, confidence interval; n, number; IV, Swiss disability insurance*

For peer review only



For peer review only - http://bmjopen.bmj.com/site/about/guidelines.xhtml

The mean age was 65 years ± 15.9 years (SD). Patients with and without sensitive topics were of comparable age. Overall, 39.3% (n = 361) of patients were female with no differences regarding the occurrence of sensitive topics to that of males. Patients' number of children, family status, citizenship, level of education, and occupation were not associated with the presence of sensitive topics. Individuals with psychiatric comorbidities, however, were more likely to have sensitive topics present (73/474 [15.4%] versus 31/445 [7.0%], $p < 0.001$; adjusted OR 1.97, 95% CI 1.21 to 3.2; $p = 0.006$). Quality of life regarding mobility, self-care, usual activities, pain / discomfort, and anxiety / depression was perceived lower by patients with sensitive topics; consequently resulting in a lower EQ-5D quality of life index score.

**Communication-related factors (Table 2)**

Patients with sensitive health topics provided more *cue*s (79/474 [16.7%] versus 32/445 [7.2%], $p < 0.001$; adjusted OR 2.36, 95%CI 1.52 to 3.66; $p < 0.001$) and *cue*s were more often addressed by physicians in patients with sensitive topics (40/474 [8.4%] versus 19/445 [4.3%], $p = 0.01$, adjusted OR 1.83, 95% CI 1.03 to 3.24; $p = 0.039$). In the subgroup of patients who mentioned emotions, physicians applied patient-centred communication techniques (i.e., NURSE, WEMS) similarly among individuals with and without sensitive topics (**Supplement Table 2**).



For peer review only - http://bmjopen.bmj.com/site/about/guidelines.xhtml

**Table 2. Associations of communication factors with sensitive topics**

| | n | No sensitive topics | Sensitive topics | *p-value* | Adjusted OR (95%CI)* | *p-value* |
|---|---|---|---|---|---|---|
| | | 445 | 474 | | | |
| **Communication factors** | 919 | | | | | |
| Visible evidence of emotion, n (%) | | 48 (10.8%) | 70 (14.8%) | *0.071* | 1.35 (0.9, 2.01) | *0.144* |
| Medical staff talks to patient about diagnosis, n (%) | | 215 (48.3%) | 271 (57.2%) | ***0.007*** | 1.59 (1.21, 2.09) | ***0.001*** |
| Medical staff talks to patient about symptoms, n (%) | | 399 (89.7%) | 443 (93.5%) | ***0.038*** | 1.66 (1.02, 2.69) | ***0.041*** |
| Medical staff talks to patient about treatment, n (%) | | 334 (75.1%) | 383 (80.8%) | ***0.036*** | 1.38 (1, 1.9) | ***0.049*** |
| Medical staff talks to patient about next steps, n (%) | | 344 (77.3%) | 431 (90.9%) | ***<0.001*** | 2.8 (1.89, 4.13) | ***<0.001*** |
| Medical staff talks to patient about social issues, n (%) | | 272 (61.1%) | 320 (67.5%) | ***0.043*** | 1.43 (1.08, 1.9) | ***0.013*** |
| Medical staff talks about patient instead of with patient after case discussion, n (%) | | 329 (73.9%) | 351 (74.1%) | *0.97* | 1.19 (0.87, 1.62) | *0.282* |
| Physician and patient talk at cross purposes, n (%) | | 17 (3.8%) | 35 (7.4%) | ***0.019*** | 2.29 (1.24, 4.25) | ***0.008*** |
| Patient seems not to understand something, but does not ask, n (%) | | 6 (1.3%) | 17 (3.6%) | ***0.030*** | 4.15 (1.55, 11.08) | ***0.005*** |
| Physician disagrees with / corrects patient, n (%) | | 30 (6.7%) | 57 (12.0%) | ***0.006*** | 2.05 (1.28, 3.3) | ***0.003*** |
| Patient disagrees with / corrects physician, n (%) | | 27 (6.1%) | 53 (11.2%) | ***0.006*** | 2.12 (1.29, 3.48) | ***0.003*** |
| Occurrence of "*cue*", n (%) | | 32 (7.2%) | 79 (16.7%) | ***<0.001*** | 2.36 (1.52, 3.66) | ***<0.001*** |
| Addressing "*cue*" concern, n (%) | | 19 (4.3%) | 40 (8.4%) | ***0.010*** | 1.83 (1.03, 3.24) | ***0.039*** |
| Reacts by providing information instead of exploration, n (%) | | 19 (4.3%) | 33 (7.0%) | *0.077* | 1.59 (0.88, 2.87) | *0.122* |

**adjusted for study centre, intervention. Odds ratios were calculated with logistic regression models. Abbreviations: OR, odds ratio; CI, confidence interval; n, number; cue, current unvoiced element*

For peer review only

**Primary and secondary outcomes (Table 3)**

Patients with sensitive topics reported lower overall satisfaction (87.7 ± 14.6 versus 90.2 ± 12.1, p = 0.006; adjusted difference -2.5, 95%CI -4.28 to -0.72; p = 0.006). Sensitive topics were associated with less subjective knowledge (77.8 ± 22 versus 81.2 ± 19, p = 0.013; adjusted difference -3.86, 95% CI -6.57 to -1.15; p = 0.005) as well as objective knowledge (68.7 ± 25.8 versus 72.6 ± 24.9, p = 0.021; adjusted difference -3.83, 95% CI -7.18 to -0.48; p = 0.025).



For peer review only - http://bmjopen.bmj.com/site/about/guidelines.xhtml

# Table 3. Association of sensitive topics with various outcomes

| | n | No sensitive topics | Sensitive topics | *p-value* | Adjusted difference or OR (95%CI)* | *p-value* |
|---|---|---|---|---|---|---|
| **Primary endpoint** | | 445 | 474 | | | |
| Overall satisfaction (VAS 0-100), mean (SD) | 906 | 90.2 (12.1) | 87.7 (14.6) | ***0.006*** | -2.5 (-4.28, -0.72) | ***0.006*** |
| **Patient knowledge about medical care** | | | | | | |
| Average subjective knowledge about their medical care, mean (SD) | 919 | 81.2 (19.0) | 77.8 (22.0) | ***0.013*** | -3.86 (-6.57, -1.15) | ***0.005*** |
| Average objective knowledge about their medical care, mean (SD) | 919 | 72.6 (24.9) | 68.7 (25.8) | ***0.021*** | -3.83 (-7.18, -0.48) | ***0.025*** |
| **Measured timeliness of ward round** | | | | | | |
| Duration of outside the room discussions (min), mean (SD) | 919 | 2.8 (3.6) | 4.9 (5.0) | ***<0.001*** | 1.41 (1.02, 1.81) | ***<0.001*** |
| Duration of bedside discussions (min), mean (SD) | 919 | 8.2 (4.2) | 9.0 (5.0) | ***0.005*** | 1.56 (1.03, 2.09) | ***<0.001*** |
| Duration of debriefing outside the room (min), mean (SD) | 919 | 0.4 (1.1) | 0.5 (1.1) | *0.063* | 0.16 (0.01, 0.31) | ***0.038*** |
| Total duration of ward round per patient (min), mean (SD) | 919 | 11.3 (4.6) | 14.5 (5.6) | ***<0.001*** | 3.13 (2.47, 3.79) | ***<0.001*** |
| **Patient perception regarding discomfort during the ward round** | | | | | | |
| Medical terms used during ward round were confusing (VAS 0-100), mean (SD) | 853 | 15.9 (28.2) | 19.2 (30.4) | *0.099* | 3.89 (-0.07, 7.85) | *0.054* |
| Ward round discussions made me worry (VAS 0-100), mean (SD) | 871 | 6.6 (25.7) | 11.7 (24.8) | ***0.003*** | 4.82 (1.4, 8.24) | ***0.006*** |
| I felt uncomfortable during ward round (VAS 0-100), mean (SD) | 870 | 4.5 (15.6) | 8.2 (21.6) | ***0.005*** | 3.2 (0.64, 5.76) | ***0.014*** |
| Ward round discussions unsettled me (VAS 0-100), mean (SD) | 752 | 4.4 (15.9) | 7.2 (19.9) | ***0.033*** | 2.84 (0.22, 5.45) | ***0.034*** |
| **Patient perception regarding physician`s behaviour during the ward round** | | | | | | |
| Physicians treated me with respect (VAS 0-100), mean (SD) | 867 | 97.4 (8.8) | 95.5 (13.1) | ***0.011*** | -2.05 (-3.57, -0.53) | ***0.008*** |
| I was taken seriously (VAS 0-100), mean (SD) | 861 | 96.1 (10.9) | 94.0 (15.6) | ***0.021*** | -2.17 (-4.01, -0.32) | ***0.021*** |
| Physicians respected my privacy (VAS 0-100), mean (SD) | 794 | 89.7 (24.8) | 91.1 (20.9) | *0.38* | 1.41 (-1.85, 4.66) | *0.396* |
| Physicians showed compassion (VAS 0-100), mean (SD) | 693 | 81.0 (28.4) | 75.2 (33.5) | ***0.014*** | -5.56 (-10.33, -0.8) | ***0.022*** |
| My issues were dealt with discreetly (VAS 0-100), mean (SD) | 764 | 88.3 (23.7) | 89.2 (21.1) | *0.55* | 1.33 (-1.91, 4.57) | *0.42* |
| Some topics during ward round communication caused inconvenience (VAS 0-100), mean (SD) | 853 | 4.0 (15.4) | 7.6 (21.8) | ***0.005*** | 3.58 (0.99, 6.17) | ***0.007*** |

**adjusted for study centre, intervention. All differences calculated with linear regression models for continuous data. Odds ratios were calculated with logistic regression models. Abbreviations: OR, odds ratio; SD, standard deviation; CI, confidence interval; VAS, visual analogue scale; min, minutes*



For peer review only - http://bmjopen.bmj.com/site/about/guidelines.xhtml

Duration of outside the room discussions, bedside discussions, and debriefings was longer in patients with sensitive topics. Mean (±SD) duration (min) of ward round per patient was 14.5 (± 5.6) versus 11.3 (± 4.6), p < 0.001; adjusted difference 3.13, 95% CI 2.47 to 3.79; p < 0.001. Compared to patients without sensitive topics, patients with sensitive topics felt more uncomfortable (4.5 ± 15.6 versus 8.2 ± 21.6, p = 0.005; adjusted difference 3.2, 95%CI 0.64 to 5.76; p = 0.014) and unsettled (4.4 ± 15.9 versus 7.2 ± 19.9, p = 0.033; adjusted difference 2.84, 95%CI 0.22 to 5.45; p = 0.034) during the ward round, and discussions causing them to worry (6.6 ± 25.7 versus 11.7 ± 24.8, p = 0.003; adjusted difference 4.82, 95% CI 1.4 to 8.24; p = 0.006). They also felt less confident with the physician team (91.9 ±14.4 versus 89.5 ± 16.8, p = 0.02; adjusted difference -2.52, 95%CI -4.65 to -0.4; p = 0.02), whereas there were no significant differences in confidence with the nursing team (**Supplement Table 3**).

Patients with sensitive topics perceived physicians less compassionate and also felt less respected, taken seriously, and more uneasy **(Table 3**).

**Risk factors for low satisfaction among patients with sensitive topics (Table 4)**

In a further step, we investigated factors associated with low satisfaction among patients with sensitive topics **(Table 4).** Several factors were associated with low satisfaction, such as parameters regarding the patient-physician interaction during ward rounds such as physicians disagreeing with patients (14/212 [6.6%] versus 41/254 [16.1%], adjusted OR 2.78 [95%CI 1.47 to 5.27], p=0.002), and patients disagreeing with physicians (14/212 [6.6%] versus 37/254 [14.6%], adjusted OR 2.42 [95%CI 1.27 to 4.61), p=0.007). Further factors were a patient’s lack of knowledge about their own medical care (both subjective and objective knowledge), an overall



For peer review only - http://bmjopen.bmj.com/site/about/guidelines.xhtml

longer duration of the ward round, reduced capacity to understand the main disease, the implemented therapeutic measure and further plans of care, as well as other physician-patient interactions (e.g., respectful treatment, physician compassion, observing privacy), see also **Supplement Table 4**.



For peer review only - http://bmjopen.bmj.com/site/about/guidelines.xhtml

**Table 4. Risk factors for low satisfaction among patients with sensitive topics**

| | n | high satisfaction | low satisfaction | *p-value* | adjusted difference or OR (95%CI)* | *p-value* |
|---|---|---|---|---|---|---|
| | | 212 | 254 | | | |
| **Other communication factors** | 466 | | | | | |
| Physician disagrees with/corrects patient, n (%) | | 14 (6.6%) | 41 (16.1%) | ***0.001*** | *2.78 (1.47, 5.27)* | ***0.002*** |
| Patient disagrees with/corrects physician, n (%) | | 14 (6.6%) | 37 (14.6%) | ***0.006*** | *2.42 (1.27, 4.61)* | ***0.007*** |
| **Patient knowledge about medical care** | 466 | | | | | |
| Average subjective knowledge about their medical care, mean (SD) | | 87.0 (16.2) | 70.7 (22.9) | ***<0.001*** | 0.95 (0.94, 0.97) | ***<0.001*** |
| Average objective knowledge about their medical care, mean (SD) | | 71.7 (25.3) | 66.2 (26.0) | ***0.023*** | 0.99 (0.98, 1) | ***0.027*** |
| **Measured timeliness of ward round** | 466 | | | | | |
| Total duration of ward round per patient (min), mean (SD) | | 13.9 (5.6) | 15.1 (5.6) | ***0.019*** | 1.05 (1.01, 1.08) | ***0.01*** |
| **Patient perception regarding time spent on ward round** | 466 | | | | | |
| Overall duration of ward round was sufficient (VAS 0-100), mean (SD) | | 95.78 (15.38) | 83.81 (24.55) | ***<0.001*** | 0.96 (0.95, 0.98) | ***<0.001*** |
| Time spent with physicians was sufficient (VAS 0-100), mean (SD) | | 96.71 (10.02) | 80.66 (22.53) | ***<0.001*** | 0.92 (0.9, 0.94) | ***<0.001*** |
| Patient estimation of time spent with patient on ward round (min), mean (SD) | | 12.18 (7.18) | 11.26 (6.58) | *0.15* | 0.98 (0.96, 1.01) | *0.176* |
| Patient estimation of time spent per day with patient case overall (min), mean (SD) | | 82.09 (86.72) | 66.73 (66.44) | ***0.031*** | 1 (0.99, 1) | ***0.035*** |
| **The ward round was helpful for better** | | | | | | |
| - understanding the main illness (VAS 0-100), mean (SD) | | 75.03 (32.91) | 54.64 (33.95) | ***<0.001*** | 0.98 (0.98, 0.99) | ***<0.001*** |
| - further therapeutic measures (VAS 0-100), mean (SD) | | 74.14 (32.45) | 53.03 (33.52) | ***<0.001*** | 0.98 (0.97, 0.99) | ***<0.001*** |
| - further plans of care (VAS 0-100), mean (SD) | | 83.33 (69.99) | 62.12 (52.73) | ***<0.001*** | 0.99 (0.98, 0.99) | ***<0.001*** |
| All my questions were answered (VAS 0-100), mean (SD) | | 97.05 (8.55) | 82.10 (24.16) | ***<0.001*** | 0.92 (0.9, 0.94) | ***<0.001*** |
| I was able to understand all answers to my questions (VAS 0-100), mean (SD) | | 96.84 (10.74) | 85.85 (20.14) | ***<0.001*** | 0.93 (0.91, 0.95) | ***<0.001*** |
| Information during visit has been adequate, n(%) | | 197 (92.9%) | 176 (69.3%) | ***<0.001*** | 0.17 (0.1, 0.31) | ***<0.001*** |
| Patient did not understand something during the round, n(%) | | 13 (6.1%) | 36 (14.2%) | ***0.005*** | 2.58 (1.32, 5.02) | ***0.005*** |
| Estimation of my participation in the discussion (VAS 0-100), mean (SD) | | 67.80 (30.61) | 51.78 (29.76) | ***<0.001*** | 0.98 (0.98, 0.99) | ***<0.001*** |
| Disease was explained in an understandable way (VAS 0-100), mean (SD) | | 91.95 (19.46) | 76.61 (27.1) | ***<0.001*** | 0.97 (0.96, 0.98) | ***<0.001*** |
| Treatment was explained in an understandable way (VAS 0-100), mean (SD) | | 91.32 (20.51) | 69.49 (31.19) | ***<0.001*** | 0.96 (0.95, 0.97) | ***<0.001*** |
| Upcoming examinations were explained in an understandable way (VAS 0-100), mean (SD) | | 91.64 (19.12) | 74.34 (28.26) | ***<0.001*** | 0.96 (0.95, 0.97) | ***<0.001*** |
| The information given during the visit was clear and understandable (VAS 0-100), mean (SD) | | 97.86 (9.04) | 82.92 (22.92) | ***<0.001*** | 0.9 (0.88, 0.93) | ***<0.001*** |
| **Patient perception regarding discomfort during the ward round** | 466 | | | | | |
| Medical terms used during ward round were confusing (VAS 0-100), mean (SD) | | 14.1 (27.87) | 23.13 (30.42) | ***0.003*** | 1.01 (1, 1.02) | ***0.003*** |
| Ward round discussions made me worry (VAS 0-100), mean (SD) | | 7.93 (22.02) | 15.86 (25.73) | ***<0.001*** | 1.02 (1.01, 1.02) | ***0.001*** |



For peer review only - http://bmjopen.bmj.com/site/about/guidelines.xhtml

| | | | | | | |
|---|---|---|---|---|---|---|
| I felt uncomfortable during ward round (VAS 0-100), mean (SD) | | 3.8 (14.88) | 11.98 (24.44) | ***<0.001*** | 1.02 (1.01, 1.04) | ***<0.001*** |
| Ward round discussions unsettled me (VAS 0-100), mean (SD) | | 2.23 (9.31) | 12.01 (22.3) | ***<0.001*** | 1.06 (1.03, 1.08) | ***<0.001*** |
| **Patient perception regarding physician`s behaviour during the ward round** | | | | | | |
| Physicians treated me with respect (VAS 0-100), mean (SD) | 466 | 99.31 (3.02) | 91.87 (16.37) | ***<0.001*** | 0.85 (0.8, 0.9) | ***<0.001*** |
| I was taken seriously (VAS 0-100), mean (SD) | 466 | 98.82 (4.97) | 88.87 (19.54) | ***<0.001*** | 0.89 (0.85, 0.92) | ***<0.001*** |
| Physicians respected my privacy (VAS 0-100), mean (SD) | 466 | 95.83 (14.64) | 86.64 (21.55) | ***<0.001*** | 0.96 (0.94, 0.98) | ***<0.001*** |
| Physicians showed compassion (VAS 0-100), mean (SD) | 466 | 83.98 (27.57) | 66.79 (30.85) | ***<0.001*** | 0.98 (0.97, 0.99) | ***<0.001*** |
| My issues were dealt with discreetly (VAS 0-100), mean (SD) | 466 | 95.01 (14.75) | 83.95 (21.16) | ***<0.001*** | 0.95 (0.94, 0.97) | ***<0.001*** |
| Some topics during ward round communication caused inconvenience (VAS 0-100), mean (SD) | 466 | 3.66 (15.99) | 10.88 (23.80) | ***<0.001*** | 1.02 (1.01, 1.03) | ***0.001*** |
| I was encouraged to address personal topics (VAS 0-100), mean (SD) | 466 | 90.57 (21.35) | 76.54 (27.65) | ***<0.001*** | 0.97 (0.96, 0.98) | ***<0.001*** |
| My privacy was violated (VAS 0-100), mean (SD) | 466 | 0.88 (5.30) | 5.19 (15.55) | ***<0.001*** | 1.07 (1.02, 1.12) | ***0.003*** |
| Teaching took place during ward round (yes, %) | 466 | 61 (28.8%) | 65 (25.6%) | *0.44* | 0.87 (0.57, 1.33) | *0.51* |
| - if yes , teaching was perceived as disruptive, (VAS 0-100) mean (SD) | 125 | 0.8 (6.4) | 10.3 (29.3) | ***0.015*** | 1.04 (1, 1.08) | *0.07* |
| **Patients' perception regarding quality of care (VAS 0-100)** | 466 | | | | | |
| I felt "in good hands" in this hospital, mean (SD) | | 96.19 (9.28) | 83.57 (17.04) | ***<0.001*** | 0.9 (0.88, 0.93) | ***<0.001*** |
| I felt there were contradicting statements from physicians and nursing team, mean (SD) | | 7.58 (20.90) | 18.87 (28.23) | ***<0.001*** | 1.02 (1.01, 1.03) | ***<0.001*** |
| I feel confident with the physician team, mean (SD) | | 95.88 (11.54) | 83.79 (18.07) | ***<0.001*** | 0.92 (0.9, 0.94) | ***<0.001*** |
| I feel confident with the nursing team, mean (SD) | | 97.45 (6.46) | 86.91 (15.65) | ***<0.001*** | 0.89 (0.87, 0.92) | ***<0.001*** |
| Physicians and nurses collaborate well, mean (SD) | | 95.68 (9.14) | 84.25 (15.8) | ***<0.001*** | 0.91 (0.89, 0.93) | ***<0.001*** |
| I feel physicians are highly competent to treat the current illness, mean (SD) | | 96.35 (9.71) | 82.84 (18.49) | ***<0.001*** | 0.9 (0.88, 0.93) | ***<0.001*** |
| I feel nurses are highly competent to treat the current illness, mean (SD) | | 96.51 (9.16) | 86.39 (31.79) | ***<0.001*** | 0.93 (0.91, 0.95) | ***<0.001*** |

*adjusted for centrum, intervention. All differences calculated with linear regression models for continuous data. Odds ratios were calculated with logistic regression models. Abbreviations: OR, odds ratio; SD, standard deviation; CI, confidence interval; VAS, visual analogue scale*

For peer review only



For peer review only - http://bmjopen.bmj.com/site/about/guidelines.xhtml

## Qualitative analysis

Examples of conversations extracted from audiotapes are provided in **Supplement Table 5**.

For peer review only



For peer review only - http://bmjopen.bmj.com/site/about/guidelines.xhtml

## Discussion

We report several key findings of this ancillary project from a multicentre randomised controlled trial that investigated sensitive topics and specific elements recorded during ward round communication: i) Sensitive topics arose frequently during ward rounds, with more than half of patients reporting at least one specific issue. Participants reporting a sensitive topic were more complex in their treatment and required more attention from medical staff, making ward rounds longer and more demanding compared to individuals without a sensitive topic; ii) Medical uncertainty was the most frequent sensitive topic; iii) The presence of sensitive topics in patientswas strongly associated with being less satisfied overall with their care; iv) Risk factors for low satisfaction among patients with sensitive topics included several parameters regarding patient-physician interaction during the ward round (various disagreements) - highlighting the importance of patient-centred communication.

Several results of the analysis provide important information and are worth further discussion. To our knowledge, this is the first trial to systematically analyse sensitive topics arising during teaching ward rounds in a typical internal medicine population. Many issues could benefit from special attention, but often take time and may not fit into the ward round time-schedule. However, it is important to address them, as patients may feel unheard and dissatisfied if these topics are not discussed (see an example of a social issue from qualitative analysis in **Box 1**).

*Patient:* I planned to move in with my partner after my hospital stay. She's a former nurse, but now a relative of hers has died.

*Chief physician:* Oh dear.

*Patient (tearful):* When she heard what could happen to me, she said no. I mean, over the last years I've burdened her with my illness. But now I am really down.

*Chief physician:* Yes, that is… I can understand that.

*Patient:* Now I had to agree with her and promised to look for assisted accommodation.



For peer review only - http://bmjopen.bmj.com/site/about/guidelines.xhtml

> *Chief physician:* Yes, we can discuss this with our case management team. Or have you already found something?
>
> *Patient:* No, because I expected to move in with her. But now her relative has passed away and it is all so complicated…
>
> *Chief physician:* Yeah, I understand! But unfortunately, we don't have much time during the ward round so we'll have to discuss your further care on another occasion.
>
> *Patient:* Okay, yes, thank you.

***Box 1:*** *Example of a patient-physician discussion about social burden*

Another frequently encountered sensitive topic concerned tumour diagnosis. Previous literature has shown an association between physicians' communicative competence and its effect on quality of life in cancer patients.[15] As quality of life was also lower in patients with sensitive topics in our study, this again underlines the importance of communication skills when talking about tumour diagnoses.

Psychiatric comorbidities and psychological issues are also perceived as sensitive topics and may be directly linked to physical disease. Physicians often hesitate to address mental health issues during the ward round because of a lack of privacy.[5] However, our results suggest that patients do not feel violated in their privacy when sensitive topics are addressed (see **Supplement Table 3**). Also, previous research has shown that 63% of patients prefer to discuss mental health issues in general, at least in the primary care setting.[16] However, similar data for the inpatient setting with less pricacy is currently lacking.

Recent studies highlight that medical uncertainty remains a significant factor in medicine despite the advances in diagnostic possibilities particularly in patients reporting various unspecific symptoms.[17] Thus, during wardrounds,  the treating team often discusses possible differentiate diagnoses of finding leaving the patient potentially with remaining uncertainty about the specific disease. This is in line with our results where medical uncertainty was reported as the most common sensitive



For peer review only - http://bmjopen.bmj.com/site/about/guidelines.xhtml

topic. Medical uncertainty has been shown to negatively affect patients' physical, and mental, well-being.[18] Moreover, dealing with medical uncertainty may also cause anxiety in physicians, negatively affect their work-related satisfaction, and result in substandard care.[19] Guidance on how best to deal with medical uncertainty and how to address it in the patient's presence is warranted to ensure both physician well-being and patient-centred care. Specific communication skills training may help achieve these aims.[17 20]

Importantly, our study found lower satisfaction in patients with sensitive topics compared to patients without sensitive topics. This may be due to confounding because patients with sensitive topics are often more complex and have different medical needs. Interestingly, previous literature in outpatient settings suggests that adequately addressing sensitive topics is associated with higher satisfaction[6 7]. Santelli et al. (2019) reported  higher levels of confidentiality during the conversation when sensitive topic were discussed.[21] We did not have information about the extend sensitive topics were discussed with patients in our study. The restricted opportunity for confidentiality during ward rounds may provide some barriers heree – even though patients did not expressly state that their privacy was violated. Limited time with the physician may be another contributor to the discrepancy in satisfaction between out- and inpatient settings. Especially time constraints during ward rounds might be a challenge to effectively address sensitive topics, compromising patient satisfaction. However, most patients stated that the time was sufficient, and the duration of ward rounds was significantly longer in patients with sensitive topics. Also, we also found the duration of ward rounds to be associated with lower satisfaction in patients with sensitive topics. It would be important to study the effect of addressing

For peer review only

sensitive topics in the inpatient population regarding satisfaction with care in an interventional study.

Over the last decades, medicine has shifted from a paternalistic to a participatory, patient-involving model, with shared decision-making as a key element. Schifferli hypothesises that disagreements between patients and physicians may be a consequence of increasing patient involvement and highlights the importance of patients' wish for participation in clinical decision-making.[22] However, not all patients may prefer shared decision- making and a personalized approach in this regard may be needed [23]. We found that disagreements between physicians and patients were a significant risk factor for low satisfaction in patients with sensitive topics. The example of our qualitative analysis in **Box 2** shows a potential for disagreement when patients wish to actively participate in the definition of treatment options, and the concept of best care differs between the patient and physician.

*Chief physician*: We know that you normally take Diazepam, which has a long half-life …

*Patient:* Yes, 24 hours.

*Chief physician*: Even longer, especially if the liver is not working properly. I've seen two people die from Diazepam. We prefer to prescribe Lorazepam

*Patient:* I do not tolerate Lorazepam.

*Chief physician*: What do you mean by "don't tolerate"?

*Patient:* Once when I withdrew from alcohol and doctors gave me Lorazepam I hallucinated. I saw a mouse in my room. Then I had delirium and woke up in another hospital.

*Chief physician*: We cannot assume that this was because of the Lorazepam. It was more likely due to alcohol withdrawal.

*Patient:* Yes, but I've never experienced anything like that with Diazepam.

*Chief physician*: It is important to me that we informed you about this …

***Box 2:*** *Example of a disagreement between patient and physician*

A recent study showed that patients with active decisional preference are less satisfied with their care and have less trust in the healthcare team compared to patients preferring less in depth involvement in medical decisions.[23] This is in line



For peer review only - http://bmjopen.bmj.com/site/about/guidelines.xhtml

with the present findings, suggesting that disagreements are a risk factor for low satisfaction. A patient's wish for participation is therefore relevant, and a more personalised approach may improve the patient-physician relationship and increase patients' satisfaction with medical care. However, as shown in our study, correctly addressing sensitive topics remains a major challenge during ward rounds due to several factors including lack of privacy, time constraints, lack of training and standards how to best address sensitive topcis among others. Future research and quality initiatives should focus on this important issue to improve the care of patients with sensitive topics.

This trial has several limitations. First, we only included Swiss teaching hospitals. This might cause a selection bias and limits generalisability of the findings. Second, using a pragmatic approach, ward rounds in the three participating hospitals were not standardised regarding clinical practice and to ensure external validity. Consequently, the internal validity might be reduced. Third, the questionnaire used to assess patient perception has not yet been validated.

**Conclusion**

This ancillary analysis suggests that a large proportion of medical inpatients have sensitive health topics, which was associated with lower satisfaction with care - particularly if the patient perceived patient-physician interactions during ward rounds as unsatisfactory. Specific physicians training in communication techniques how to identify, discuss and address sensitive topics may help to improve the care of these patients.



For peer review only - http://bmjopen.bmj.com/site/about/guidelines.xhtml

## Data Sharing Statement

The data will be made available to parties placing a reasonable request to the lead author.

## Author statement

All listed authors were involved in conducting the present study.

SG designed, analysed, and interpreted this ancillary analysis, and drafted the article.

CB designed the original trial, wrote the proposal for the ethics committee, provided study material or patients and critically revised the manuscript for important intellectual content.

KB collected patient data and critically revised the manuscript for important intellectual content.

VM analysed and interpreted patient data and critically revised the manuscript for important intellectual content.

JG contributed to design of this ancillary analyses and critically revised the manuscript for important intellectual content.

PS designed the original trial, contributed statistical expertise, and critically revised the manuscript for important intellectual content.

JL provided study material or patients and critically revised the manuscript for important intellectual content.



For peer review only - http://bmjopen.bmj.com/site/about/guidelines.xhtml

RS contributed to the study design and critically revised the manuscript for important intellectual content.

WL designed the original trial and critically revised the manuscript for important intellectual content.

MT provided study material or patients and critically revised the manuscript for important intellectual content.

TB provided study material or patients and critically revised the manuscript for important intellectual content.

JE provided study material or patients and critically revised the manuscript for important intellectual content.

MO provided study material or patients and critically revised the manuscript for important intellectual content.

SB designed the original trial, provided study material or patients, and critically revised the manuscript for important intellectual content.

SH designed the original trial, wrote the proposal for the ethics committee, analysed and interpreted patient data, drafted the article, critically revised the manuscript for important intellectual content, provided study material or patients, contributed statistical expertise, and obtained funding.

All authors approved the final manuscript.

## Financial disclosures



For peer review only - http://bmjopen.bmj.com/site/about/guidelines.xhtml

This work was supported by the Swiss National Science Foundation grant number SNSF_182422.

S. Hunziker and her research team are supported by the Swiss National Science Foundation (SNSF) (Ref 10001C_192850/1 and 10531C_182422) and the Gottfried Julia Bangerter-Rhyner Foundation (8472/HEG-DSV).

P. Schuetz has received support from the SNF (SNSF Professorship, PP00P3_150531), the Research Committee (Forschungsrat) of the Kantonsspital Aarau (1410.000.058 and 1410.000.044) and Funds of the Argovia Professorship of the Medical University Clinic (FG 1500000083). P. Schuetz has previously received unrestricted grant money unrelated to this project from Nestlé Health Science and Abbott Nutrition.

JD Leuppi and his research team are supported by the Swiss Personalized Health Network (Ref Driver-Project – 2018DR108)) and the Swiss National Science Foundation (SNSF) (Ref SNFS-160072 und -185592). JD Leuppi has also received unrestricted grant money unrelated to the project from AstraZeneca AG Switzerland, Boehringer GmbH Switzerland, GSK AG Switzerland, and Novartis AG Switzerland.

RS received funding from the Stanley Thomas Johnson Stiftung & Gottfried und Julia Bangerter-Rhyner-Stiftung under projects no. PC 28/17 and PC 05/18, from the Swiss Cancer League (Krebsliga Schweiz) under project no. KLS-4304-08-2017, from Promotion Santé Suisse *(Gesundheitsförderung Schweiz)* under contract no. 18.191/ K50001, and in the context of a Horizon Europe project from the Swiss State Secretariat for Education, Research and Innovation (SERI) under contract number 22.00094.RS received a speaker honorarium from Novartis.



For peer review only - http://bmjopen.bmj.com/site/about/guidelines.xhtml

MT is recipient of a project grant of the Swiss National Science Foundation (grant No. 320030_200423) and has research collaborations with Roche, Novartis and Idorsia (all Switzerland).

**Competing interest**

All authors report no conflicts of interest.

For peer review only

## References

1. Cowles KV. Issues in qualitative research on sensitive topics. *West J Nurs Res* 1988;10(2):163-79. doi: 10.1177/019394598801000205 [published Online First: 1988/04/01]

2. Weber H, Stockli M, Nubling M, et al. Communication during ward rounds in internal medicine. An analysis of patient-nurse-physician interactions using RIAS. *Patient Educ Couns* 2007;67(3):343-8. doi: 10.1016/j.pec.2007.04.011 [published Online First: 2007/06/08]

3. Ahluwalia SC, Levin JR, Lorenz KA, et al. Missed opportunities for advance care planning communication during outpatient clinic visits. *Journal of General Internal Medicine* 2012;27(4):445-51. doi: 10.1007/s11606-011-1917-0

4. Becker C, Gamp M, Schuetz P, et al. Effect of Bedside Compared With Outside the Room Patient Case Presentation on Patients' Knowledge About Their Medical Care : A Randomized, Controlled, Multicenter Trial. *Ann Intern Med* 2021;174(9):1282-92. doi: 10.7326/M21-0909 [published Online First: 2021/06/29]

5. Gross S, Beck K, Becker C, et al. Perception of physicians and nursing staff members regarding outside versus bedside ward rounds: ancillary analysis of the randomised BEDSIDE-OUTSIDE trial. *Swiss Medical Weekly* 2022;152 doi: ARTN w30112

10.4414/SMW.2022.w30112

6. Brahmania M, Young M, Muthiah C, et al. Resident trainees do not affect patient satisfaction in an outpatient gastroenterology clinic: A prospective study conducted in a Canadian gastroenterology clinic. *Can J Gastroenterol Hepatol*;29(7):363-68.

7. Brown JD, Wissow LS. Discussion of sensitive health topics with youth during primary care visits: relationship to youth perceptions of care. *J Adolesc Health* 2009;44(1):48-54. doi: 10.1016/j.jadohealth.2008.06.018 [published Online First: 2008/12/23]

8. Schulz KF, Altman DG, Moher D, et al. CONSORT 2010 Statement: updated guidelines for reporting parallel group randomised trials. *Trials* 2010;11:32. doi: 10.1186/1745-6215-11-32 [published Online First: 2010/03/26]

9. Kerim-Sade C, Crispin A, Wasem J. An external control of validity of the German EuroQol-5D questionnaire. *Wirtschaftswissenschaftliche Diskussionspapiere* 2000;No. 06/2000

10. Lesley F. Degner JAS, Peri Venkatesh. The Control Preference Scale. *Canadian Journal of Nursing Research* 1997;29(3):21-43.



For peer review only - http://bmjopen.bmj.com/site/about/guidelines.xhtml

11. Degner L, Kristjanson L, Bowman D, et al. Information Needs and Decisional Preferences in Women With Breast Cancer. *JAMA* 1997;277(18):1485-92.

12. von Campenhausen J. Ärztliche Kommunikation für Medizinstudierende.: Springer Berlin Heidelberg. 2020.

13. Pollak KI, Arnold RM, Jeffreys AS, et al. Oncologist communication about emotion during visits with patients with advanced cancer. *Journal of Clinical Oncology* 2007;25(36):5748-52. doi: 10.1200/Jco.2007.12.4180

14. Zimmermann C, Del Piccolo L, Finset A. Cues and concerns by patients in medical consultations: a literature review. *Psychol Bull* 2007;133(3):438-63. doi: 10.1037/0033-2909.133.3.438

15. Yoon H, Chatters L, Kao TS, et al. Factors Affecting Quality of Life for Korean American Cancer Survivors: An Integrative Review. *Oncol Nurs Forum* 2016;43(3):E132-42. doi: 10.1188/16.ONF.E132-E142

16. Lish JD, Kuzma MA, Lush DT, et al. PSYCHIATRIC SCREENING IN PRIMARY CARE: WHAT DO PATIENTS REALLY WANT? *Journal of Psychosomatic Research* 1997;43:167-75.

17. Alam R, Cheraghi-Sohi S, Panagioti M, et al. Managing diagnostic uncertainty in primary care: a systematic critical review. *BMC Fam Pract* 2017;18(1):79. doi: 10.1186/s12875-017-0650-0 [published Online First: 20170807]

18. Guan T, Santacroce SJ, Chen DG, et al. Illness uncertainty, coping, and quality of life among patients with prostate cancer. *Psycho-oncology* 2020;29(6):1019-25. doi: 10.1002/pon.5372

19. Bovier PA, Perneger TV. Stress from uncertainty from graduation to retirement - A population-based study of Swiss physicians. *Journal of general internal medicine* 2007;22(5):632-38. doi: 10.1007/s11606-007-0159-7

20. Papanagnou D, Ankam N, Ebbott D, et al. Towards a medical school curriculum for uncertainty in clinical practice. *Med Educ Online* 2021;26(1):1972762. doi: 10.1080/10872981.2021.1972762 [published Online First: 2021/08/31]

21. Santelli JS, Klein JD, Song X, et al. Discussion of Potentially Sensitive Topics With Young People. *Pediatrics* 2019;143(2) doi: 10.1542/peds.2018-1403 [published Online First: 20190116]

22. Schifferli JA. [Shared decision making: a new challenge for the physicians?]. *Praxis (Bern 1994)* 2005;94(39):1505-7. doi: 10.1024/0369-8394.94.39.1505

23. Becker C, Gross S, Gamp M, et al. Patients' Preference for Participation in Medical Decision-Making: Secondary Analysis of the BEDSIDE-OUTSIDE Trial. *J Gen Intern Med* 2022 doi: 10.1007/s11606-022-07775-z [published Online First: 2022/09/10]

For peer review only

## Figure legends

### Figure 1. Sensitive topics and their frequency (n=791)



For peer review only - http://bmjopen.bmj.com/site/about/guidelines.xhtml

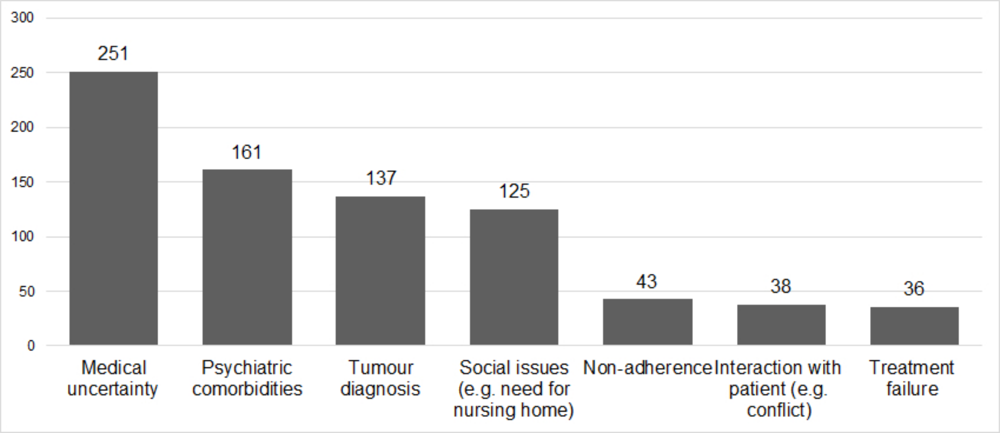


Figure 1. Sensitive topics and their frequency (n=791)

299x129mm (72 x 72 DPI)

# Supplementary Appendix

For peer review only

## Supplement 1. Associations of patient characteristics with sensitive topics

| | n | All | No Sensitive topics | Sensitive topics | *p-value* | univariable OR (95%CI) | *p-value* | OR (95%CI) *adjusted for centrum, intervention* | *p-value* |
|---|---|---|---|---|---|---|---|---|---|
| **n** | | | 445 | 474 | | | | | |
| **Sociodemographic factors** | | | | | | | | | |
| *Age, years; mean (SD)* | 919 | 65.0 (15.9) | 65.7 (15.9) | 64.3 (15.9) | *0.16* | 0.99 (0.99, 1) | *0.164* | 1 (0.99, 1) | *0.304* |
| *Female sex, n (%)* | 919 | 361 (39.3%) | 173 (38.9%) | 188 (39.7%) | *0.81* | 1.03 (0.79, 1.35) | *0.807* | 1.05 (0.8, 1.37) | *0.741* |
| *Number of children, mean (SD)* | 919 | 2.5 (9.2) | 2.1 (6.7) | 2.8 (11.0) | *0.26* | 1.01 (0.99, 1.02) | *0.275* | 1.01 (0.99, 1.02) | *0.365* |
| **Family status** | 919 | | | | | | | | |
| Single, relationship, married, registered partnership, n (%) | | 648 (70.5%) | 317 (71.2%) | 331 (69.8%) | *0.84* | 1 (ref.) | | | |
| Separated, divorced, n (%) | | 147 (16.0%) | 68 (15.3%) | 79 (16.7%) | | 1.11 (0.78, 1.59) | *0.56* | 1.05 (0.73, 1.52) | *0.787* |
| Widowed, n (%) | | 124 (13.5%) | 60 (13.5%) | 64 (13.5%) | | 1.02 (0.7, 1.5) | *0.913* | 1.07 (0.72, 1.59) | *0.738* |
| **Citizenship** | 919 | | | | | | | | |
| Switzerland, n (%) | | 789 (85.9%) | 386 (86.7%) | 403 (85.0%) | *0.71* | 1 (ref.) | | | |
| Germany, n (%) | | 55 (6.0%) | 26 (5.8%) | 29 (6.1%) | | 1.07 (0.62, 1.85) | *0.813* | 0.97 (0.55, 1.68) | *0.904* |
| Other, n (%) | | 75 (8.2%) | 33 (7.4%) | 42 (8.9%) | | 1.22 (0.76, 1.96) | *0.416* | 1.22 (0.75, 1.98) | *0.421* |
| **Highest level of education** | | | | | | | | | |
| No School graduation, n (%) | | 23 (2.5%) | 10 (2.2%) | 13 (2.7%) | *0.86* | 1 (ref.) | | | |
| School graduation, n (%) | | 141 (15.3%) | 67 (15.1%) | 74 (15.6%) | | 0.85 (0.35, 2.07) | *0.719* | 0.96 (0.39, 2.35) | *0.929* |
| Apprenticeship/Diploma, n (%) | | 631 (68.7%) | 309 (69.4%) | 322 (67.9%) | | 0.8 (0.35, 1.86) | *0.605* | 0.92 (0.39, 2.15) | *0.846* |
| University/Higher Education, n (%) | | 118 (12.8%) | 55 (12.4%) | 63 (13.3%) | | 0.88 (0.36, 2.17) | *0.783* | 0.89 (0.36, 2.21) | *0.799* |
| Missings, n (%) | | 6 (0.7%) | 4 (0.9%) | 2 (0.4%) | | 0.38 (0.06, 2.54) | *0.321* | 0.39 (0.06, 2.62) | *0.336* |
| **Occupation** | 919 | | | | | | | | |
| Employed / working + IV, n (%) | | 259 (28.2%) | 129 (29.0%) | 130 (27.4%) | ***0.041*** | 1 (ref.) | | | |
| Unemployed / homemaker, n (%) | | 39 (4.2%) | 13 (2.9%) | 26 (5.5%) | | 1.98 (0.98, 4.03) | *0.058* | 2 (0.97, 4.09) | *0.059* |
| Retired / IV support, n (%) | | 605 (65.8%) | 296 (66.5%) | 309 (65.2%) | | 1.04 (0.77, 1.39) | *0.812* | 1.05 (0.78, 1.42) | *0.735* |
| In education, n (%) | | 6 (0.7%) | 5 (1.1%) | 1 (0.2%) | | 0.2 (0.02, 1.72) | *0.142* | 0.19 (0.02, 1.63) | *0.128* |
| Other, n (%) | | 10 (1.1%) | 2 (0.4%) | 8 (1.7%) | | 3.97 (0.83, 19.05) | *0.085* | 3.41 (0.7, 16.56) | *0.128* |
| **Health-related factors** | 919 | | | | | | | | |
| ***Main admission diagnosis*** | | | | | | | | | |
| Coronary heart disease, n (%) | | 93 (10.1%) | 53 (11.9%) | 40 (8.4%) | ***<0.001*** | 1 (ref.) | | | |
| Congestive heart failure, n (%) | | 80 (8.7%) | 38 (8.5%) | 42 (8.9%) | | 1.46 (0.8, 2.67) | *0.213* | 1.7 (0.92, 3.15) | *0.09* |
| Cardiovascular diseases, n (%) | | 98 (10.7%) | 56 (12.6%) | 42 (8.9%) | | 0.99 (0.56, 1.76) | *0.983* | 1.09 (0.61, 1.95) | *0.78* |
| Infections, n (%) | | 207 (22.5%) | 116 (26.1%) | 91 (19.2%) | | 1.04 (0.63, 1.7) | *0.878* | 1.2 (0.73, 1.99) | *0.468* |
| Gastrointestinal diseases, n (%) | | 58 (6.3%) | 31 (7.0%) | 27 (5.7%) | | 1.15 (0.6, 2.23) | *0.67* | 1.15 (0.59, 2.25) | *0.687* |
| Metabolisms, n (%) | | 51 (5.5%) | 30 (6.7%) | 21 (4.4%) | | 0.93 (0.46, 1.85) | *0.831* | 1 (0.49, 2.02) | *0.999* |
| Tumour, n (%) | | 81 (8.8%) | 36 (8.1%) | 45 (9.5%) | | 1.66 (0.91, 3.02) | *0.1* | 1.64 (0.89, 3.04) | *0.115* |
| Other, n (%) | | 251 (27.3%) | 85 (19.1%) | 166 (35.0%) | | 2.59 (1.59, 4.21) | ***<0.001*** | 3.08 (1.87, 5.07) | ***<0.001*** |
| ***Comorbidities*** | 919 | | | | | | | | |
| Charlson Comorbidity Index, mean (SD) | | 4.45 (2.88) | 4.4 (2.8) | 4.5 (3.0) | *0.54* | 1.01 (0.97, 1.06) | *0.543* | 0.76 (0.59, 1) | ***0.048*** |
| Cardiology, n (%) | | 485 (52.8%) | 248 (55.7%) | 237 (50.0%) | *0.082* | 1.02 (0.98, 1.07) | *0.373* | 0.86 (0.65, 1.13) | *0.273* |
| Neurology, n (%) | | 193 (21.0%) | 80 (18.0%) | 113 (23.8%) | ***0.029*** | 0.79 (0.61, 1.03) | *0.082* | 1.01 (0.77, 1.33) | *0.941* |
| Rheumatology / Immunology, n (%) | | 143 (15.6%) | 68 (15.3%) | 75 (15.8%) | *0.82* | 0.82 (0.63, 1.07) | *0.15* | 1.03 (0.78, 1.37) | *0.819* |
| Gastrointestinal, n (%) | | 254 (27.6%) | 118 (26.5%) | 136 (28.7%) | *0.46* | 1.43 (1.04, 1.97) | ***0.03*** | 1.18 (0.85, 1.65) | *0.325* |
| Endocrinology, n (%) | | 352 (38.3%) | 185 (41.6%) | 167 (35.2%) | ***0.048*** | 1.46 (1.06, 2.03) | *0.022* | 1.21 (0.86, 1.71) | *0.264* |
| Respiratory, n (%) | | 315 (34.3%) | 152 (34.2%) | 163 (34.4%) | *0.94* | 1.04 (0.73, 1.49) | *0.821* | 1.07 (0.81, 1.4) | *0.627* |
| Infectious diseases, n (%) | | 171 (18.6%) | 77 (17.3%) | 94 (19.8%) | *0.33* | 1.11 (0.77, 1.62) | *0.57* | 1.12 (0.85, 1.48) | *0.416* |

For peer review only

| | | | | | | | | | |
|---|---|---|---|---|---|---|---|---|---|
| Renal, n (%) | | 317 (34.5%) | 150 (33.7%) | 167 (35.2%) | *0.63* | 1.12 (0.83, 1.49) | *0.461* | 0.25 (0.07, 0.91) | ***0.035*** |
| Gynaecology, n (%) | | 14 (1.5%) | 11 (2.5%) | 3 (0.6%) | ***0.023*** | 1.18 (0.87, 1.58) | *0.284* | 0.26 (0.07, 0.94) | ***0.04*** |
| Urology, n (%) | | 93 (10.1%) | 43 (9.7%) | 50 (10.5%) | *0.66* | 1.1 (0.72, 1.69) | *0.657* | 1.24 (0.8, 1.93) | *0.338* |
| Oncology, n (%) | | 287 (31.2%) | 145 (32.6%) | 142 (30.0%) | *0.39* | 0.88 (0.67, 1.17) | *0.391* | 1.81 (1.13, 2.9) | ***0.014*** |
| Psychiatry, n (%) | | 104 (11.3%) | 31 (7.0%) | 73 (15.4%) | ***<0.001*** | 2.43 (1.56, 3.78) | ***<0.001*** | 1.97 (1.21, 3.2) | ***0.006*** |
| Depression, n (%) | | 82 (8.9%) | 29 (6.5%) | 53 (11.2%) | ***0.013*** | 2.43 (1.56, 3.78) | ***<0.001*** | 0.89 (0.68, 1.15) | *0.364* |
| Other, n (%) | | 498 (54.2%) | 248 (55.7%) | 250 (52.7%) | *0.36* | 2.54 (1.62, 3.99) | ***<0.001*** | 0.95 (0.73, 1.25) | *0.734* |
| **Health self rating VAS [0-100], mean (SD)** | 919 | 56.9 (22.32) | 58.64 (21.97) | 55.25 (22.55) | ***0.021*** | 0.99 (0.99, 1) | ***0.022*** | 0.99 (0.99, 1) | ***0.018*** |
| **Quality of life (EQ-5D) Index, mean (SD)** | 919 | 0.71 (0.3) | 0.75 (0.27) | 0.67 (0.3) | ***<0.001*** | 0.38 (0.24, 0.6) | ***<0.001*** | 0.4 (0.25, 0.65) | ***<0.001*** |
| **Patient's Decisional Control Preferences** | 919 | | | | | | | | |
| SDM, n (%) | | 473 (62.2%) | 234 (61.1%) | 239 (63.2%) | *0.54* | 1 (ref.) | | | |
| active, n (%) | | 117 (15.4%) | 57 (14.9%) | 60 (15.9%) | *0.70* | 1.19 (0.84, 1.69) | *0.332* | 1.22 (0.86, 1.75) | *0.268* |
| passive, n (%) | | 171 (22.5%) | 92 (24.0%) | 79 (20.9%) | *0.30* | 1.23 (0.77, 1.96) | *0.397* | 1.24 (0.77, 2) | *0.382* |
| **Intervention of the original study, n %** | 919 | | | | | | | | |
| Bedside ward round | | 476 (51.8%) | 257 (57.8%) | 219 (46.2%) | ***<0.001*** | 1 (ref.) | | | |
| Outside the rooum ward round | | 443 (48.2%) | 188 (42.2%) | 255 (53.8%) | | 0.63 (0.48, 0.82) | ***<0.001*** | 0.64 (0.49, 0.83)* | ***0.001*** |

**adjusted for study centre, intervention. Odds ratios were calculated with logistic regression models. Abbreviations: OR, odds ratio; SD, standard deviation; CI, confidence interval; IV, Swiss disability insurance; n, number*

For peer review only

## Supplement 2. Associations of communication factors with sensitive topics

| | n | No Sensitive topics | Sensitive topics | *p-value* | OR (95%CI) | *p-value* | Adjusted OR (95%CI) *adjusted for centrum, intervention* | *p-value* |
|---|---|---|---|---|---|---|---|---|
| | | 445 | 474 | | | | | |
| **WEMS-Technique communication** | 919 | | | | | | | |
| Waiting, n (%) | | 71 (16.0%) | 55 (11.6%) | 0.055 | 0.69 (0.47, 1.01) | 0.056 | 0.83 (0.56, 1.24) | 0.37 |
| Echoing, n (%) | | 108 (24.3%) | 107 (22.6%) | 0.54 | 0.91 (0.67, 1.23) | 0.544 | 1.02 (0.74, 1.41) | 0.902 |
| Mirroring, n (%) | | 52 (11.7%) | 40 (8.4%) | 0.10 | 0.7 (0.45, 1.08) | 0.103 | 0.82 (0.52, 1.28) | 0.377 |
| Summarising, n (%) | | 44 (9.9%) | 39 (8.2%) | 0.38 | 0.82 (0.52, 1.28) | 0.381 | 0.84 (0.53, 1.33) | 0.464 |
| Open-ended questions, n (%) | | 352 (79.1%) | 348 (73.4%) | **0.043** | 0.73 (0.54, 0.99) | **0.044** | 0.77 (0.56, 1.05) | 0.102 |
| Confirming, n (%) | | 211 (47.4%) | 195 (41.1%) | 0.056 | 0.78 (0.6, 1.01) | 0.056 | 0.79 (0.61, 1.03) | 0.084 |
| Nodding, n (%) | | 344 (77.3%) | 362 (76.4%) | 0.74 | 0.95 (0.7, 1.29) | 0.738 | 1.09 (0.79, 1.49) | 0.611 |
| **NURSE-Technique communication in patients with emotions (n = 115)** | 115 | 34 | 81 | | | | | |
| Visible evidence of emotion, n (%) | | 7 (21%) | 16 (20%) | 0.92 | 0.95 (0.35, 2.57) | 0.919 | 0.95 (0.33, 2.78) | 0.929 |
| Understanding (verbal/nonverbal), n (%) | | 8 (24%) | 23 (28%) | 0.59 | 1.29 (0.51, 3.26) | 0.592 | 1.24 (0.46, 3.35) | 0.667 |
| Respecting, n (%) | | 4 (12%) | 26 (32%) | **0.023** | 3.55 (1.13, 11.12) | **0.03** | 2.7 (0.79, 9.25) | 0.114 |
| Supporting, n (%) | | 9 (26%) | 25 (31%) | 0.64 | 1.24 (0.51, 3.04) | 0.638 | 1.24 (0.48, 3.23) | 0.66 |
| Exploring, n (%) | | 9 (26%) | 25 (31%) | 0.64 | 1.24 (0.51, 3.04) | 0.638 | 1.24 (0.48, 3.23) | 0.66 |
| Hasty solace, n (%) | | 0 (0%) | 3 (4%) | 0.26 | n.a. | n.a. | n.a. | n.a. |
| Distracting/Ignoring, n (%) | | 0 (0%) | 3 (4%) | 0.26 | n.a. | n.a. | n.a. | n.a. |
| Dismissing ("that's not so bad"), n (%) | | 4 (12%) | 4 (5%) | 0.19 | 0.39 (0.09, 1.66) | 0.202 | 0.58 (0.12, 2.84) | 0.505 |
| Replies with providing information instead of addressing emotion, n (%) | | 15 (44%) | 37 (46%) | 0.88 | 1.07 (0.48, 2.38) | 0.878 | 1.13 (0.48, 2.66) | 0.785 |
| Physician talks about emotion, n (%) | | 4 (12%) | 9 (11%) | 0.92 | 0.94 (0.27, 3.28) | 0.92 | 0.79 (0.21, 3.03) | 0.734 |
| Overall rating: Responding to emotions, n (%) | | 14 (44%) | 28 (35%) | 0.39 | 0.69 (0.3, 1.6) | 0.389 | 0.64 (0.26, 1.61) | 0.346 |
| **Other communication factors** | 919 | | | | | | | |
| Occurrence of emotion, n (%) | | 48 (10.8%) | 70 (14.8%) | 0.071 | 1.43 (0.97, 2.12) | 0.072 | 1.35 (0.9, 2.01) | 0.144 |
| Medical staff talks to patient about diagnosis, n (%) | | 215 (48.3%) | 271 (57.2%) | **0.007** | 1.43 (1.1, 0) | **0.007** | 1.59 (1.21, 2.09) | **0.001** |
| Medical staff talks to patient about symptoms, n (%) | | 399 (89.7%) | 443 (93.5%) | **0.038** | 1.65 (1.02, 2.65) | **0.039** | 1.66 (1.02, 2.69) | **0.041** |
| Medical staff talks to patient about treatment, n (%) | | 334 (75.1%) | 383 (80.8%) | **0.036** | 1.4 (1.02, 1.91) | **0.036** | 1.38 (1, 1.9) | **0.049** |
| Medical staff talks to patient about next steps, n (%) | | 344 (77.3%) | 431 (90.9%) | **<0.001** | 2.94 (2, 4.32) | **<0.001** | 2.8 (1.89, 4.13) | **<0.001** |
| Medical staff talks to patient about social issues, n (%) | | 272 (61.1%) | 320 (67.5%) | **0.043** | 1.32 (1.01, 1.73) | 0.043 | 1.43 (1.08, 1.9) | **0.013** |
| Medical staff talks about patient instead of with patient after case discussion, n (%) | | 329 (73.9%) | 351 (74.1%) | 0.97 | 1.01 (0.75, 1.35) | 0.967 | 1.19 (0.87, 1.62) | 0.282 |
| Physician and patient talk at cross purposes, n (%) | | 17 (3.8%) | 35 (7.4%) | **0.019** | 2.01 (1.11, 3.64) | **0.022** | 2.29 (1.24, 4.25) | **0.008** |
| Patient seems not to understand something, but does not ask, n (%) | | 6 (1.3%) | 17 (3.6%) | **0.030** | 2.72 (1.06, 6.97) | **0.037** | 4.15 (1.55, 11.08) | **0.005** |
| Physician disagrees with / corrects patient, n (%) | | 30 (6.7%) | 57 (12.0%) | **0.006** | 1.89 (1.19, 3) | **0.007** | 2.05 (1.28, 3.3) | **0.003** |
| Patient disagrees with / corrects physician, n (%) | | 27 (6.1%) | 53 (11.2%) | **0.006** | 1.95 (1.2, 3.16) | **0.007** | 2.12 (1.29, 3.48) | **0.003** |
| Occurrence of *"cue"*, n (%) | | 32 (7.2%) | 79 (16.7%) | **<0.001** | 2.58 (1.67, 3.98) | **<0.001** | 2.36 (1.52, 3.66) | **<0.001** |
| Addressing *"cue"* concern, n (%) | | 19 (4.3%) | 40 (8.4%) | **0.010** | 2.07 (1.18, 3.63) | **0.011** | 1.83 (1.03, 3.24) | **0.039** |
| Reacts by providing information instead of exploration, n (%) | | 19 (4.3%) | 33 (7.0%) | 0.077 | 1.68 (0.94, 3) | 0.08 | 1.59 (0.88, 2.87) | 0.122 |

**adjusted for study centre, intervention. Odds ratios were calculated with logistic regression models. Abbreviations: OR, odds ratio; CI, confidence interval; cue, current unvoiced element; n, number*

For peer review only

# Supplement 3. Association of sensitive topics with various outcomes

| | n | No Sensitive topics | Sensitive topics | *p-value* | Difference or OR (95%CI) | *p-value* | Adjusted difference or OR (95%CI) *adjusted for centrum, intervention* | *p-value* |
|---|---|---|---|---|---|---|---|---|
| **Primary endpoint** | | 445 | 474 | | | | | |
| Overall satisfaction (VAS 0-100), mean (SD) | 906 | 90.2 (12.1) | 87.7 (14.6) | **0.006** | -2.49 (-4.24, -0.73) | **0.006** | -2.5 (-4.28, -0.72) | **0.006** |
| **Secondary endpoints** | | | | | | | | |
| **Satisfaction with care (VAS 0-100)** | | | | | | | | |
| Overall satisfaction with ward round, mean (SD) | 898 | 89.9 (16.1) | 86.6 (19.9) | **0.006** | -3.33 (-5.71, -0.95) | **0.006** | -3.51 (-5.93, -1.09) | **0.005** |
| Overall satisfaction with hospital stay, mean (SD) | 901 | 88.3 (17.0) | 86.8 (16.7) | 0.18 | -1.52 (-3.72, 0.68) | 0.176 | -1.1 (-3.32, 1.11) | 0.328 |
| Overall satisfaction with medical care, mean (SD) | 891 | 91.9 (14.1) | 90.0 (16.0) | 0.069 | -1.84 (-3.83, 0.14) | 0.069 | -1.97 (-3.99, 0.06) | 0.057 |
| Overall satisfaction with communication with physicians during ward round, mean (SD) | 886 | 90.7 (15.9) | 86.2 (21.7) | **<0.001** | -4.48 (-7.01, -1.95) | **0.001** | -4.39 (-6.97, -1.82) | **0.001** |
| Overall satisfaction with communication with nursing team during ward round , mean (SD) | 622 | 92.3 (16.6) | 90.0 (17.7) | 0.099 | -2.28 (-4.99, 0.43) | 0.099 | -2.45 (-5.21, 0.31) | 0.082 |
| **Patient knowledge about medical care** | | | | | | | | |
| Average subjective knowledge about their medical care, mean (SD) | 919 | 81.2 (19.0) | 77.8 (22.0) | **0.013** | -3.38 (-6.05, -0.71) | **0.013** | -3.86 (-6.57, -1.15) | **0.005** |
| Subjective understanding of disease, mean (SD) | 902 | 82.7 (19.8) | 80.4 (21.8) | 0.092 | -2.34 (-5.07, 0.39) | 0.092 | -2.7 (-5.48, 0.08) | 0.057 |
| Subjective understanding of therapeutic approach, mean (SD) | 861 | 80.8 (23.3) | 76.0 (27.8) | **0.006** | -4.83 (-8.28, -1.38) | **0.006** | -5.77 (-9.25, -2.29) | **0.001** |
| Subjective understanding of further plans for care, mean (SD) | 851 | 79.8 (25.0) | 76.1 (27.8) | **0.042** | -3.71 (-7.27, -0.14) | **0.042** | -3.99 (-7.61, -0.37) | **0.031** |
| Average objective knowledge about their medical care, mean (SD) | 919 | 72.6 (24.9) | 68.7 (25.8) | **0.021** | -3.88 (-7.17, -0.6) | **0.021** | -3.83 (-7.18, -0.48) | 0.025 |
| Objective understanding of disease, mean (SD) | 916 | 71.5 (32.2) | 66.3 (33.4) | **0.016** | -5.22 (-9.48, -0.96) | **0.016** | -5.24 (-9.59, -0.9) | **0.018** |
| Objective understanding of therapeutic approach, mean (SD) | 908 | 71.3 (34.2) | 68.4 (35.0) | 0.21 | -2.9 (-7.42, 1.61) | 0.208 | -2.39 (-6.99, 2.22) | 0.309 |
| Objective understanding of further plans for care, mean (SD) | 916 | 75.3 (31.5) | 71.2 (34.0) | 0.061 | -4.08 (-8.34, 0.19) | 0.061 | -4.4 (-8.73, -0.06) | 0.047 |
| **Measured timeliness of ward round** | | | | | | | | |
| Duration of outside the room discussions (min), mean (SD) | 919 | 2.8 (3.6) | 4.9 (5.0) | **<0.001** | 2.17 (1.6, 2.73) | **<0.001** | 1.41 (1.02, 1.81) | **<0.001** |
| Duration of bedside discussions (min), mean (SD) | 919 | 8.2 (4.2) | 9.0 (5.0) | **0.005** | 0.88 (0.27, 1.48) | **0.005** | 1.56 (1.03, 2.09) | **<0.001** |
| Duration of debriefing outside the room (min), mean (SD) | 919 | 0.4 (1.1) | 0.5 (1.1) | 0.063 | 0.14 (-0.01, 0.29) | 0.063 | 0.16 (0.01, 0.31) | **0.038** |
| Total duration of ward round per patient (min), mean (SD) | 919 | 11.3 (4.6) | 14.5 (5.6) | **<0.001** | 3.18 (2.52, 3.85) | **<0.001** | 3.13 (2.47, 3.79) | **<0.001** |
| **Patient perception regarding time spent on ward round** | | | | | | | | |
| Overall duration of ward round was sufficient (VAS 0-100), mean (SD) | 859 | 91.2 (18.3) | 89.9 (21.9) | 0.35 | -1.3 (-4, 1.41) | 0.346 | -1.21 (-3.98, 1.55) | 0.389 |
| Time spent with physicians was sufficient (VAS 0-100), mean (SD) | 771 | 90.2 (18.0) | 89.3 (20.0) | 0.52 | -0.88 (-3.58, 1.81) | 0.52 | -0.89 (-3.64, 1.86) | 0.525 |
| Patients estimation on the time spent with patient on ward round (min), mean (SD) | 856 | 11.2 (6.0) | 11.7 (7.1) | 0.27 | 0.5 (-0.39, 1.38) | 0.271 | 0.94 (0.06, 1.81) | **0.036** |
| Patients estimation on the time spent per day with patient case overall (min), mean (SD) | 719 | 69.6 (78.0) | 73.2 (85.1) | 0.55 | 3.63 (-8.34, 15.6) | 0.552 | 5.26 (-6.9, 17.42) | 0.396 |
| The ward round was helpful for better | | | | | | | | |
| - understanding the main illness (VAS 0-100), mean (SD) | 773 | 69.5 (34.3) | 62.7 (37.3) | **0.009** | -6.75 (-11.82, -1.69) | **0.009** | -6.21 (-11.4, -1.01) | **0.019** |
| - further therapeutic measures (VAS 0-100), mean (SD) | 714 | 68.7 (34.6) | 60.5 (37.2) | **0.002** | -8.22 (-13.51, -2.92) | **0.002** | -7.77 (-13.19, -2.36) | **0.005** |
| - further plans of care (VAS 0-100), mean (SD) | 755 | 71.1 (33.9) | 69.9 (67.5) | 0.76 | -1.21 (-8.88, 6.47) | 0.758 | -1.49 (-9.33, 6.35) | 0.71 |
| All my questions were answered (VAS 0-100), mean (SD) | 752 | 93.0 (15.6) | 89.7 (21.0) | **0.017** | -3.25 (-5.91, -0.59) | **0.017** | -3.29 (-6.01, -0.57) | **0.018** |
| I was able to understand all answers to my questions (VAS 0-100), mean (SD) | 747 | 93.3 (15.3) | 91.6 (18.3) | 0.19 | -1.64 (-4.07, 0.8) | 0.187 | -1.94 (-4.43, 0.54) | 0.125 |
| Information during visit has been adequate (yes, %) | 907 | 358 (81.2%) | 370 (79.4%) | 0.50 | 0.89 (0.64, 1.24) | 0.501 | 0.89 (0.64, 1.25) | 0.501 |
| Patient did not understand something during the round (yes, %) | 908 | 54 (12.2%) | 49 (10.5%) | 0.41 | 0.84 (0.56, 1.27) | 0.406 | 0.91 (0.6, 1.39) | 0.679 |
| Estimation of my participation in the discussion (VAS 0-100), mean (SD) | 837 | 63.8 (38.0) | 59.3 (32.2) | 0.064 | -4.51 (-9.28, 0.27) | 0.064 | -4.67 (-9.5, 0.16) | 0.058 |
| Disease was explained in an understandable way (VAS 0-100), mean (SD) | 720 | 84.3 (27.0) | 84.7 (26.3) | 0.84 | 0.4 (-3.5, 4.3) | 0.841 | 0.16 (-3.81, 4.12) | 0.939 |
| Treatment was explained in an understandable way (VAS 0-100), mean (SD) | 707 | 82.1 (28.5) | 80.5 (30.2) | 0.48 | -1.56 (-5.9, 2.78) | 0.48 | -1.61 (-6.04, 2.81) | 0.474 |
| Upcoming examinations were explained in an understandable way (VAS 0-100), mean (SD) | 635 | 82.6 (27.9) | 83.5 (28.2) | 0.71 | 0.84 (-3.53, 5.22) | 0.705 | 0.96 (-3.51, 5.43) | 0.673 |
| The information given during the visit was clear and understandable (VAS 0-100), mean (SD) | 859 | 92.1 (15.9) | 90.3 (19.6) | 0.15 | -1.76 (-4.16, 0.63) | 0.149 | -1.78 (-4.23, 0.67) | 0.154 |
| **Patient perception regarding discomfort during the ward round** | | | | | | | | |
| Medical terms used during ward round were confusing (VAS 0-100), mean (SD) | 853 | 15.9 (28.2) | 19.2 (30.4) | 0.099 | 3.32 (-0.63, 7.27) | 0.099 | 3.89 (-0.07, 7.85) | 0.054 |
| Ward round discussions made me worry (VAS 0-100), mean (SD) | 871 | 6.6 (25.7) | 11.7 (24.8) | **0.003** | 5.13 (1.78, 8.49) | **0.003** | 4.82 (1.4, 8.24) | **0.006** |
| I felt uncomfortable during ward round (VAS 0-100), mean (SD) | 870 | 4.5 (15.6) | 8.2 (21.6) | **0.005** | 3.61 (1.1, 6.13) | **0.005** | 3.2 (0.64, 5.76) | **0.014** |
| Ward round discussions unsettled me (VAS 0-100), mean (SD) | 752 | 4.4 (15.9) | 7.2 (19.9) | **0.033** | 2.81 (0.23, 5.4) | **0.033** | 2.84 (0.22, 5.45) | **0.034** |
| **Patient perception regarding physician`s behaviour during the ward round** | | | | | | | | |

For peer review only

| | | | | | | | | |
|---|---|---|---|---|---|---|---|---|
| Physicians treated me with respect (VAS 0-100), mean (SD) | 867 | 97.4 (8.8) | 95.5 (13.1) | ***0.011*** | -1.94 (-3.43, -0.45) | ***0.011*** | -2.05 (-3.57, -0.53) | ***0.008*** |
| I was taken seriously (VAS 0-100), mean (SD) | 861 | 96.1 (10.9) | 94.0 (15.6) | ***0.021*** | -2.13 (-3.94, -0.32) | ***0.021*** | -2.17 (-4.01, -0.32) | ***0.021*** |
| Physicians respected my privacy (VAS 0-100), mean (SD) | 794 | 89.7 (24.8) | 91.1 (20.9) | *0.38* | 1.42 (-1.78, 4.61) | *0.384* | 1.41 (-1.85, 4.66) | *0.396* |
| Physicians showed compassion (VAS 0-100), mean (SD) | 693 | 81.0 (28.4) | 75.2 (33.5) | ***0.014*** | -5.86 (-10.53, -1.2) | ***0.014*** | -5.56 (-10.33, -0.8) | ***0.022*** |
| My issues were dealt with discreetly (VAS 0-100), mean (SD) | 764 | 88.3 (23.7) | 89.2 (21.1) | *0.55* | 0.97 (-2.21, 4.16) | *0.549* | 1.33 (-1.91, 4.57) | *0.42* |
| Some topics during ward round communication caused inconvenience (VAS 0-100), mean (SD) | 853 | 4.0 (15.4) | 7.6 (21.8) | ***0.005*** | 3.61 (1.07, 6.15) | ***0.005*** | 3.58 (0.99, 6.17) | ***0.007*** |
| I was encouraged to address personal topics (VAS 0-100), mean (SD) | 624 | 85.5 (30.5) | 84.4 (30.2) | *0.65* | -1.11 (-5.88, 3.66) | *0.648* | -1.41 (-6.28, 3.46) | *0.57* |
| My privacy was violated (VAS 0-100), mean (SD) | 831 | 4.2 (16.2) | 3.3 (13.3) | *0.38* | -0.9 (-2.91, 1.12) | *0.383* | -1.17 (-3.23, 0.88) | *0.264* |
| Teaching took place during ward round (yes, %) | 909 | 136 (30.8%) | 126 (26.9%) | *0.19* | 0.83 (0.62, 1.1) | *0.193* | 0.96 (0.71, 1.3) | *0.812* |
| - if yes , teaching was perceived as disruptive, (VAS 0-100) mean (SD) | 257 | 1.7 (8.4) | 5.7 (21.9) | *0.051* | 4.01 (-0.02, 8.05) | ***0.05*** | 4.14 (0.08, 8.2) | ***0.046*** |
| **Patients' perception regarding quality of care (VAS 0-100)** | | | | | | | | |
| I felt "in good hands" in this hospital, mean (SD) | 883 | 90.6 (15.1) | 89.4 (15.6) | *0.27* | -1.15 (-3.18, 0.88) | *0.266* | -0.68 (-2.75, 1.38) | *0.516* |
| I felt there were contradicting statements from physicians and nursing team , mean (SD) | 843 | 11.6 (24.8) | 13.5 (26.6) | *0.27* | 1.95 (-1.53, 5.43) | *0.272* | 1.55 (-2, 5.1) | *0.392* |
| I feel confident with the physician team, mean (SD) | 873 | 91.9 (14.4) | 89.5 (16.8) | ***0.025*** | -2.39 (-4.47, -0.31) | ***0.025*** | -2.52 (-4.65, -0.4) | ***0.02*** |
| I feel confident with the nursing team, mean (SD) | 876 | 93.2 (12.6) | 91.7 (13.7) | *0.093* | -1.49 (-3.24, 0.25) | *0.093* | -1.7 (-3.48, 0.08) | *0.061* |
| There is good collaboration of physicians and nurses, mean (SD) | 809 | 92.3 (12.4) | 89.6 (14.9) | ***0.007*** | -2.63 (-4.54, -0.73) | ***0.007*** | -2.95 (-4.89, -1.01) | ***0.003*** |
| I feel physicians have high competence to treat the current illness , mean (SD) | 834 | 93.3 (43.0) | 89.4 (16.9) | *0.081* | -3.93 (-8.34, 0.48) | *0.081* | -3.66 (-8.16, 0.84) | *0.111* |
| I feel nurses have high competence to treat the current illness, mean (SD) | 845 | 92.7 (11.5) | 91.2 (25.9) | *0.28* | -1.5 (-4.21, 1.21) | *0.279* | -1.62 (-4.38, 1.15) | *0.251* |

*adjusted for study centre, intervention. All differences calculated with linear regression models for continuous data. Odds ratios were calculated with logistic regression models. Abbreviations: OR, odds ratio; SD, standard deviation; CI, confidence interval; VAS, visual analogue scale; n, number*

For peer review only

## Supplement 4. Risk factors for low satisfaction among patients with sensitive topics

| | n | high Satisfaction | low Satisfaction | *p-value* | Difference or OR (95%CI) | *p-value* | Adjusted difference or OR (95%CI) *adjusted for centrum, intervention* | *p-value* |
|---|---|---|---|---|---|---|---|---|
| | | 212 | 254 | | | | | |
| **WEMS-Technique communication** | 466 | | | | | | | |
| Waiting, n (%) | | 24 (11.3%) | 30 (11.8%) | *0.87* | 1.05 (0.59, 1.86) | *0.869* | *1.09 (0.61, 1.96)* | *0.766* |
| Echoing, n (%) | | 43 (20.3%) | 64 (25.2%) | *0.21* | 1.32 (0.85, 2.05) | *0.21* | *1.47 (0.92, 2.33)* | *0.107* |
| Mirroring, n (%) | | 14 (6.6%) | 26 (10.2%) | *0.16* | 1.61 (0.82, 3.17) | *0.166* | *1.75 (0.87, 3.51)* | *0.115* |
| Summarising, n (%) | | 18 (8.5%) | 21 (8.3%) | *0.93* | 0.97 (0.5, 1.88) | *0.931* | *1.01 (0.52, 1.96)* | *0.982* |
| Open-ended questions, n (%) | | 158 (74.5%) | 188 (74.0%) | *0.90* | 0.97 (0.64, 1.48) | *0.9* | *1 (0.65, 1.52)* | *0.982* |
| Confirming, n (%) | | 90 (42.5%) | 102 (40.2%) | *0.62* | 0.91 (0.63, 1.32) | *0.616* | *0.92 (0.63, 1.33)* | *0.64* |
| Nodding, n (%) | | 160 (75.5%) | 197 (77.6%) | *0.60* | 1.12 (0.73, 1.73) | *0.596* | *1.16 (0.75, 1.79)* | *0.505* |
| **NURSE-Technique communication (in patients with emotions)** | | 34 | 46 | | | | | |
| Naming (of emotion), n (%) | 78 | 8 (24%) | 7 (15%) | *0.35* | 0.58 (0.19, 1.8) | *0.35* | *0.46 (0.14, 1.47)* | *0.189* |
| Understanding (verbal/nonverbal), n (%) | 78 | 8 (24%) | 15 (33%) | *0.38* | 1.57 (0.58, 4.29) | *0.377* | *1.59 (0.55, 4.61)* | *0.395* |
| Respecting, n (%) | 78 | 13 (38%) | 12 (26%) | *0.25* | 0.57 (0.22, 1.48) | *0.249* | *0.37 (0.13, 1.09)* | *0.07* |
| Supporting, n (%) | 78 | 13 (38%) | 12 (26%) | *0.25* | 0.57 (0.22, 1.48) | *0.249* | *0.54 (0.2, 1.48)* | *0.232* |
| Exploring, n (%) | 78 | 13 (38%) | 12 (26%) | *0.25* | 0.57 (0.22, 1.48) | *0.249* | *0.54 (0.2, 1.48)* | *0.232* |
| Hasty solcae, n (%) | 78 | 1 (3%) | 2 (4%) | *0.74* | 1.5 (0.13, 17.25) | *0.745* | *1.54 (0.13, 19.05)* | *0.735* |
| Distracting/Ignoring, n (%) | 78 | 1 (3%) | 2 (4%) | *0.74* | 1.5 (0.13, 17.25) | *0.745* | *1.54 (0.13, 19.05)* | *0.735* |
| Dismissing (“that’s not so bad”), n (%) | 78 | 2 (6%) | 2 (4%) | *0.76* | 0.73 (0.1, 5.44) | *0.756* | *0.61 (0.08, 4.69)* | *0.636* |
| Replies with providing information instead of addressing emotion, n (%) | 78 | 16 (47%) | 20 (43%) | *0.75* | 0.87 (0.36, 2.11) | *0.75* | *0.88 (0.35, 2.23)* | *0.793* |
| Physician talks about emotion, n (%) | 78 | 6 (18%) | 3 (7%) | *0.12* | 0.33 (0.08, 1.41) | *0.133* | *0.35 (0.07, 1.67)* | *0.188* |
| Overall rating: Responding to emotions, n (%) | 77 | 13 (38%) | 14 (31%) | *0.51* | 0.73 (0.29, 1.86) | *0.509* | *0.98 (0.36, 2.67)* | *0.969* |
| **Other communication factors** | 466 | | | | | | | |
| Medical staff talks to patient about diagnosis, n (%) | | 117 (55.2%) | 149 (58.7%) | *0.45* | 1.15 (0.8, 1.67) | *0.451* | *1.19 (0.82, 1.73)* | *0.368* |
| Medical staff talks to patient about symptoms, n (%) | | 198 (93.4%) | 237 (93.3%) | *0.97* | 0.99 (0.47, 2.05) | *0.969* | *1.02 (0.49, 2.12)* | *0.963* |
| Medical staff talks to patient about treatment, n (%) | | 167 (78.8%) | 209 (82.3%) | *0.34* | 1.25 (0.79, 1.98) | *0.34* | *1.23 (0.78, 1.96)* | *0.375* |
| Medical staff talks to patient about next steps, n (%) | | 190 (89.6%) | 234 (92.1%) | *0.35* | 1.35 (0.72, 2.56) | *0.349* | *1.33 (0.71, 2.52)* | *0.376* |

For peer review only

| | | | | | | | | |
|---|---|---|---|---|---|---|---|---|
| Medical staff talks to patient about social issues, n (%) | | 151 (71.2%) | 164 (64.6%) | 0.13 | 0.74 (0.5, 1.09) | 0.127 | 0.75 (0.5, 1.12) | 0.163 |
| Medical staff talks about instead of with the patient after case discussion, n (%) | | 157 (74.1%) | 189 (74.4%) | 0.93 | 1.02 (0.67, 1.55) | 0.931 | 1.07 (0.7, 1.65) | 0.748 |
| Physician and patient talk talk at cross purposes, n (%) | | 17 (8.0%) | 16 (6.3%) | 0.47 | 0.77 (0.38, 1.57) | 0.472 | 0.78 (0.38, 1.58) | 0.486 |
| Patient does not seem to understand something, but does not ask, n (%) | | 9 (4.2%) | 8 (3.1%) | 0.53 | 0.73 (0.28, 1.94) | 0.531 | 0.78 (0.29, 2.11) | 0.624 |
| Physician disagrees with/corrects patient, n (%) | | 14 (6.6%) | 41 (16.1%) | **0.001** | 2.72 (1.44, 5.15) | **0.002** | 2.78 (1.47, 5.27) | **0.002** |
| Patient disagrees with/corrects physician, n (%) | | 14 (6.6%) | 37 (14.6%) | **0.006** | 2.41 (1.27, 4.59) | **0.007** | 2.42 (1.27, 4.61) | **0.007** |
| Occurrence of cue, n (%) | | 28 (13.2%) | 49 (19.3%) | 0.078 | 1.57 (0.95, 2.6) | 0.08 | 1.55 (0.93, 2.57) | 0.093 |
| Addressing cue concern, n (%) | | 17 (8.0%) | 22 (8.7%) | 0.80 | 1.09 (0.56, 2.11) | 0.803 | 1.05 (0.54, 2.04) | 0.888 |
| Replies with providing information instead of exploration, n (%) | | 10 (4.7%) | 22 (8.7%) | 0.094 | 1.92 (0.89, 4.14) | 0.098 | 1.91 (0.88, 4.14) | 0.1 |
| **Patient knowledge about medical care** | 466 | | | | | | | |
| Average subjective knowledge about their medical care, mean (SD) | | 87.0 (16.2) | 70.7 (22.9) | **<0.001** | 0.96 (0.94, 0.97) | **<0.001** | 0.95 (0.94, 0.97) | **<0.001** |
| Subjective understanding of disease, mean (SD) | | 88.48 (16.27) | 74.14 (23.03) | **<0.001** | 0.96 (0.95, 0.97) | **<0.001** | 0.96 (0.95, 0.97) | **<0.001** |
| Subjective understanding of therapeutic approach, mean (SD) | | 85.18 (23.36) | 69.55 (27.99) | **<0.001** | 0.98 (0.97, 0.98) | **<0.001** | 0.97 (0.96, 0.98) | **<0.001** |
| Subjective understanding of further plans for care, mean (SD) | | 87.14 (22.39) | 68.21 (27.81) | **<0.001** | 0.97 (0.96, 0.98) | **<0.001** | 0.97 (0.96, 0.98) | **<0.001** |
| Average objective knowledge about their medical care, mean (SD) | | 71.7 (25.3) | 66.2 (26.0) | **0.023** | 0.99 (0.98, 1) | **0.024** | 0.99 (0.98, 1) | **0.027** |
| Objective understanding of disease, mean (SD) | | 68.42 (33.64) | 64.5 (33.26) | 0.21 | 1 (0.99, 1) | 0.207 | 1 (0.99, 1) | 0.229 |
| Objective understanding of therapeutic approach, mean (SD) | | 72.52 (33.61) | 65.35 (35.44) | **0.026** | 0.99 (0.99, 1) | **0.027** | 0.99 (0.99, 1) | **0.026** |
| Objective understanding of further plans for care, mean (SD) | | 74.01 (33.45) | 68.8 (34.46) | 0.10 | 1 (0.99, 1) | 0.101 | 1 (0.99, 1) | 0.112 |
| **Measured timeliness of ward round** | 466 | | | | | | | |
| Duration of outside the room discussions (min), mean (SD) | | 4.6 (4.8) | 5.2 (5.0) | 0.22 | 1.02 (0.99, 1.06) | 0.215 | 1.05 (0.99, 1.1) | 0.095 |
| Duration of bedside discussions (min), mean (SD) | | 8.7 (5.0) | 9.4 (5.0) | 0.18 | 1.03 (0.99, 1.06) | 0.179 | 1.04 (1, 1.09) | 0.078 |
| Duration of debriefing outside the room (min), mean (SD) | | .53 (1.1) | .57 (1.2) | 0.81 | 1.02 (0.87, 1.2) | 0.807 | 1.03 (0.87, 1.21) | 0.739 |
| Total duration of ward round per patient (min), mean (SD) | | 13.9 (5.6) | 15.1 (5.6) | **0.019** | 1.04 (1.01, 1.08) | **0.02** | 1.05 (1.01, 1.08) | **0.01** |
| **Patient perception regarding time spent on ward round** | 466 | | | | | | | |
| Overall duration of ward round was sufficient (VAS 0-100), mean (SD) | | 95.78 (15.38) | 83.81 (24.55) | **<0.001** | 0.97 (0.95, 0.98) | **<0.001** | 0.96 (0.95, 0.98) | **<0.001** |
| Time spent with physicians was sufficient (VAS 0-100), mean (SD) | | 96.71 (10.02) | 80.66 (22.53) | **<0.001** | 0.92 (0.9, 0.94) | **<0.001** | 0.92 (0.9, 0.94) | **<0.001** |
| Patient estimation of time spent with patient on ward round (min), mean (SD) | | 12.18 (7.18) | 11.26 (6.58) | 0.15 | 0.98 (0.96, 1.01) | 0.154 | 0.98 (0.96, 1.01) | 0.176 |
| Patient estimation of time spent per day with patient case overall (min), mean (SD) | | 82.09 (86.72) | 66.73 (66.44) | **0.031** | 1 (0.99, 1) | **0.036** | 1 (0.99, 1) | **0.035** |
| The ward round was helpful for better | | | | | | | | |
| - understanding the main illness (VAS 0-100), mean (SD) | | 75.03 (32.91) | 54.64 (33.95) | **<0.001** | 0.98 (0.98, 0.99) | **<0.001** | 0.98 (0.98, 0.99) | **<0.001** |
| - further therapeutic measures (VAS 0-100), mean (SD) | | 74.14 (32.45) | 53.03 (33.52) | **<0.001** | 0.98 (0.97, 0.99) | **<0.001** | 0.98 (0.97, 0.99) | **<0.001** |
| - further plans of care (VAS 0-100), mean (SD) | | 83.33 (69.99) | 62.12 (52.73) | **<0.001** | 0.99 (0.98, 0.99) | **<0.001** | 0.99 (0.98, 0.99) | **<0.001** |
| All my questions were answered (VAS 0-100), mean (SD) | | 97.05 (8.55) | 82.10 (24.16) | **<0.001** | 0.92 (0.9, 0.94) | **<0.001** | 0.92 (0.9, 0.94) | **<0.001** |

For peer review only

| | | | | | | | | |
|---|---|---|---|---|---|---|---|---|
| I was able to understand all answers to my questions (VAS 0-100), mean (SD) | | 96.84 (10.74) | 85.85 (20.14) | <0.001 | 0.93 (0.91, 0.95) | <0.001 | 0.93 (0.91, 0.95) | <0.001 |
| Information during visit has been adequate (yes, %) | | 197 (92.9%) | 176 (69.3%) | <0.001 | 0.17 (0.1, 0.31) | <0.001 | 0.17 (0.1, 0.31) | <0.001 |
| Patient did not understand something during the round (yes, %) | | 13 (6.1%) | 36 (14.2%) | 0.005 | 2.53 (1.3, 4.9) | 0.006 | 2.58 (1.32, 5.02) | 0.005 |
| Estimation of my participation in the discussion (VAS 0-100), mean (SD) | | 67.80 (30.61) | 51.78 (29.76) | <0.001 | 0.98 (0.98, 0.99) | <0.001 | 0.98 (0.98, 0.99) | <0.001 |
| Disease was explained in an understandable way (VAS 0-100), mean (SD) | | 91.95 (19.46) | 76.61 (27.1) | <0.001 | 0.97 (0.96, 0.98) | <0.001 | 0.97 (0.96, 0.98) | <0.001 |
| Treatment was explained in an understandable way (VAS 0-100), mean (SD) | | 91.32 (20.51) | 69.49 (31.19) | <0.001 | 0.96 (0.95, 0.97) | <0.001 | 0.96 (0.95, 0.97) | <0.001 |
| Upcoming examinations were explained in an understandable way (VAS 0-100), mean (SD) | | 91.64 (19.12) | 74.34 (28.26) | <0.001 | 0.96 (0.95, 0.98) | <0.001 | 0.96 (0.95, 0.97) | <0.001 |
| The information given during the visit was clear and understandable (VAS 0-100), mean (SD) | | 97.86 (9.04) | 82.92 (22.92) | <0.001 | 0.9 (0.88, 0.93) | <0.001 | 0.9 (0.88, 0.93) | <0.001 |
| **Patient perception regarding discomfort during the ward round** | 466 | | | | | | | |
| Medical terms used during ward round were confusing (VAS 0-100), mean (SD) | | 14.1 (27.87) | 23.13 (30.42) | 0.003 | 1.01 (1, 1.02) | 0.003 | 1.01 (1, 1.02) | 0.003 |
| Ward round discussions made me worry (VAS 0-100), mean (SD) | | 7.93 (22.02) | 15.86 (25.73) | <0.001 | 1.01 (1.01, 1.02) | 0.001 | 1.02 (1.01, 1.02) | 0.001 |
| I felt uncomfortable during ward round (VAS 0-100), mean (SD) | | 3.8 (14.88) | 11.98 (24.44) | <0.001 | 1.02 (1.01, 1.04) | <0.001 | 1.02 (1.01, 1.04) | <0.001 |
| Ward round discussions unsettled me (VAS 0-100), mean (SD) | | 2.23 (9.31) | 12.01 (22.3) | <0.001 | 1.06 (1.03, 1.08) | <0.001 | 1.06 (1.03, 1.08) | <0.001 |
| **Patient perception regarding physician`s behaviour during the ward round** | | | | | | | | |
| Physicians treated me with respect (VAS 0-100), mean (SD) | 466 | 99.31 (3.02) | 91.87 (16.37) | <0.001 | 0.85 (0.8, 0.9) | <0.001 | 0.85 (0.8, 0.9) | <0.001 |
| I was taken seriously (VAS 0-100), mean (SD) | 466 | 98.82 (4.97) | 88.87 (19.54) | <0.001 | 0.89 (0.85, 0.92) | <0.001 | 0.89 (0.85, 0.92) | <0.001 |
| Physicians respected my privacy (VAS 0-100), mean (SD) | 466 | 95.83 (14.64) | 86.64 (21.55) | <0.001 | 0.96 (0.94, 0.98) | <0.001 | 0.96 (0.94, 0.98) | <0.001 |
| Physicians showed compassion (VAS 0-100), mean (SD) | 466 | 83.98 (27.57) | 66.79 (30.85) | <0.001 | 0.98 (0.97, 0.99) | <0.001 | 0.98 (0.97, 0.99) | <0.001 |
| My issues were dealt with discreetly (VAS 0-100), mean (SD) | 466 | 95.01 (14.75) | 83.95 (21.16) | <0.001 | 0.95 (0.94, 0.97) | <0.001 | 0.95 (0.94, 0.97) | <0.001 |
| Some topics during ward round communication caused inconvenience (VAS 0-100), mean (SD) | 466 | 3.66 (15.99) | 10.88 (23.80) | <0.001 | 1.02 (1.01, 1.03) | 0.001 | 1.02 (1.01, 1.03) | 0.001 |
| I was encouraged to address personal topics (VAS 0-100), mean (SD) | 466 | 90.57 (21.35) | 76.54 (27.65) | <0.001 | 0.97 (0.96, 0.98) | <0.001 | 0.97 (0.96, 0.98) | <0.001 |
| My privacy was violated (VAS 0-100), mean (SD) | 466 | 0.88 (5.30) | 5.19 (15.55) | <0.001 | 1.07 (1.02, 1.12) | 0.003 | 1.07 (1.02, 1.12) | 0.003 |
| Teaching took place during ward round (yes, %) | 466 | 61 (28.8%) | 65 (25.6%) | 0.44 | 0.85 (0.57, 1.28) | 0.441 | 0.87 (0.57, 1.33) | 0.51 |
| - if yes , teaching was perceived as disruptive, (VAS 0-100) mean (SD) | 125 | 0.8 (6.4) | 10.3 (29.3) | 0.015 | 1.04 (0.99, 1.08) | 0.09 | 1.04 (1, 1.08) | 0.07 |
| **Patients' perception regarding quality of care (VAS 0-100)** | 466 | | | | | | | |
| I felt "in good hands" in this hospital, mean (SD) | | 96.19 (9.28) | 83.57 (17.04) | <0.001 | 0.91 (0.88, 0.93) | <0.001 | 0.9 (0.88, 0.93) | <0.001 |
| I felt there were contradicting statements from physicians and nursing team, mean (SD) | | 7.58 (20.90) | 18.87 (28.23) | <0.001 | 1.02 (1.01, 1.03) | <0.001 | 1.02 (1.01, 1.03) | <0.001 |
| I feel Confident with the physician team, mean (SD) | | 95.88 (11.54) | 83.79 (18.07) | <0.001 | 0.92 (0.9, 0.94) | <0.001 | 0.92 (0.9, 0.94) | <0.001 |
| I feel Confident with the nursing team, mean (SD) | | 97.45 (6.46) | 86.91 (15.65) | <0.001 | 0.89 (0.87, 0.92) | <0.001 | 0.89 (0.87, 0.92) | <0.001 |
| There is good collaboration of physicians and nurses, mean (SD) | | 95.68 (9.14) | 84.25 (15.8) | <0.001 | 0.91 (0.89, 0.93) | <0.001 | 0.91 (0.89, 0.93) | <0.001 |
| I feel physicians have high competence to treat the current illness, mean (SD) | | 96.35 (9.71) | 82.84 (18.49) | <0.001 | 0.9 (0.88, 0.93) | <0.001 | 0.9 (0.88, 0.93) | <0.001 |
| I feel nurses have high competence to treat the current illness, mean (SD) | | 96.51 (9.16) | 86.39 (31.79) | <0.001 | 0.92 (0.9, 0.94) | <0.001 | 0.93 (0.91, 0.95) | <0.001 |

For peer review only

**adjusted for study centre, intervention. All differences calculated with linear regression models for continuous data. Odds ratios were calculated with logistic regression models. Abbreviations: OR, odds ratio; SD, standard deviation; CI, confidence interval; VAS, visual analogue scale; n, number*

For peer review only

## Supplement 5. Examples of discussions addressing delicate topics

| Number of Example | Sensitive Topic | Predictor | Transcript | Current Location | Intervention group |
|---|---|---|---|---|---|
| 1 | Medical ambiguity | Physician disagrees with/corrects patient | Chief physician (CP): We know that you normally take Diazepam, which has a long half-life…<br>Patient (P): Yes, 24 hours.<br>Chief physician: Even longer, especially if the liver is not working properly. I've seen two people die from Diazepam. We prefer to prescribe Lorazepam<br>Patient: I do not tolerate Lorazepam.<br>Chief physician: What do you mean by “don’t tolerate”?<br>Patient: Once when I withdrew from alcohol and doctors gave me Lorazepam I hallucinated. I saw a mouse in my room. Then I had a delirium and woke up in another hospital.<br>Chief physician: We cannot assume that this was because of the Lorazepam. It was more likely due to the alcohol withdrawal.<br>Patient: Yes, but I've never experienced anything like that with Diazepam.<br>Chief physician: It is important to me that we informed you about this… | bedside | outside |
| 2 | Medical ambiguity | Occurrence of cue | CP: So far, we have tried many different medications, but we cannot solve the issue with drugs alone. In my opinion an intervention is necessary, maybe even two. The goal is to improve the function of your heart and thereby improve your breathing.<br>P: And this intervention is nothing I need to worry about?<br>CP: Well, an intervention is always an intervention. It’s never no risk, right? But it is a low risk I have to say. | bedside | outside |
| 3 | Psychiatric comorbidities | Physician disagrees with/corrects patient | CP: The functioning of your liver is very reduced. After all, it was poisoned by the alcohol, and now you have a cirrhosis.<br>P: A cirrhosis already?<br>CP: Certainly, an early stage of a cirrhosis, yes. If someone does drink such a high amount over such a long time…<br>P: I did drink for 4 weeks only.<br>CP: Yes, but you have a history of 26 years of recurrent relapses.<br>P: Yes, but you did an ultrasound last time and I was told everything was fine?<br>CP: Well, everything fine would be an exaggeration. A photo of someone only gives limited information about her or his character. It's the same with the liver.<br>P: Okay… | bedside | outside |
| 4 | Tumour diagnosis | Distracting/Ignoring emotion | CP: Did anyone close to you have had stomach problems?<br>P: Yes, my siblings have. They had cancer as well. And my husband, of course.<br>CP: Mhm, I am sorry about this.<br>P: Yeah, well no one can do anything about it. I’ll have to deal with it by myself, right? Because it still hurts. It was not long ago.<br>Nurse (N): How long?<br>P: 4 years.<br>CA: What about diarrhoea? Anyone close to you suffered from diarrhoea?<br>P: I don’t know. | bedside | bedside |
| 5 | Social issues (e.g. need for nursing home) | NURSE-Technique, Understanding | Patient: I planned to move in with my partner after my hospital stay. She’s a former nurse, but now a relative of hers has died.<br>Chief physician: Oh dear.<br>Patient (tearful): When she heard what could happen to me, she said no. I mean, over the last years I’ve burdened her with my illness. But now I am really down.<br>Chief physician: Yes, that is… I can understand that.<br>Patient: Now I had to agree with her and promised to look for assisted accommodation.<br>Chief physician: Yes, we can discuss this with our case management team. Or have you already found something?<br>Patient: No, because I expected to move in with her. But now her relative has passed away and it is all so complicated…<br>Chief physician: Yeah, I understand! But unfortunately, we don’t have much time during the ward round so | bedside | outside |

| | | | | | |
|---|---|---|---|---|---|
| | | | we'll have to discuss your further care on another occasion.<br>Patient: Okay, yes, thank you. | | |
| 6 | Non-adherence | Medical staff talks to patient about treatment | CP: Your gastrointestinal tract is attacked by your immune system due to the transplant (GvHD). You will receive some medication against it. However, treatment is kind of tricky as you must take that medication very carefully. To treat this, you need to be more compliant with the medication.<br>P: I did so.<br>CP: Sure…<br>P: We already talked about that last time. I took my medication but at times that were more comfortable for me.<br>CP: This is okay, as long as the time interval between the intake of the Cyclosporine remains the same.<br>P: Yeah, I tried…<br>CP: So, you are aware, that this is important?<br>P: Yes, I am. | bedside | bedside |

For peer review only

# CONSORT 2010 checklist of information to include when reporting a randomised trial

| Section/Topic | Item No | Checklist item | Reported on page No |
|---|---|---|---|
| **Title and abstract** | | | |
| | 1a | Identification as a randomised trial in the title | 1 |
| | 1b | Structured summary of trial design, methods, results, and conclusions (for specific guidance see CONSORT for abstracts) | 3 |
| **Introduction** | | | |
| Background and objectives | 2a | Scientific background and explanation of rationale | 5-6 |
| | 2b | Specific objectives or hypotheses | 6 |
| **Methods** | | | |
| Trial design | 3a | Description of trial design (such as parallel, factorial) including allocation ratio | 6 |
| | 3b | Important changes to methods after trial commencement (such as eligibility criteria), with reasons | n.a. |
| Participants | 4a | Eligibility criteria for participants | See original trial (Becker et al, 2021) |
| | 4b | Settings and locations where the data were collected | 6-8 |
| Interventions | 5 | The interventions for each group with sufficient details to allow replication, including how and when they were actually administered | 7 |
| Outcomes | 6a | Completely defined pre-specified primary and secondary outcome measures, including how and when they were assessed | 8-10 |
| | 6b | Any changes to trial outcomes after the trial commenced, with reasons | n.a. |
| Sample size | 7a | How sample size was determined | See original trial (Becker et al, 2021) |
| | 7b | When applicable, explanation of any interim analyses and stopping guidelines | n.a. |
| Randomisation: | | | |
| Sequence generation | 8a | Method used to generate the random allocation sequence | See original trial (Becker et al, 2021) |
| | 8b | Type of randomisation; details of any restriction (such as blocking and block size) | See original |

For peer review only



For peer review only - http://bmjopen.bmj.com/site/about/guidelines.xhtml

| | | | |
|---|---|---|---|
| | | | trial (Becker et al, 2021) |
| Allocation concealment mechanism | 9 | Mechanism used to implement the random allocation sequence (such as sequentially numbered containers), describing any steps taken to conceal the sequence until interventions were assigned | See original trial (Becker et al, 2021) |
| Implementation | 10 | Who generated the random allocation sequence, who enrolled participants, and who assigned participants to interventions | See original trial (Becker et al, 2021) |
| Blinding | 11a | If done, who was blinded after assignment to interventions (for example, participants, care providers, those assessing outcomes) and how | 7, see also at original trial (Becker et al, 2021) |
| | 11b | If relevant, description of the similarity of interventions | See original trial (Becker et al, 2021) |
| Statistical methods | 12a | Statistical methods used to compare groups for primary and secondary outcomes | 10 |
| | 12b | Methods for additional analyses, such as subgroup analyses and adjusted analyses | 10 |
| **Results** | | | |
| Participant flow (a diagram is strongly recommended) | 13a | For each group, the numbers of participants who were randomly assigned, received intended treatment, and were analysed for the primary outcome | See original trial (Becker et al, 2021)/ 12(Table1) |
| | 13b | For each group, losses and exclusions after randomisation, together with reasons | See original trial (Becker et al, 2021) |
| Recruitment | 14a | Dates defining the periods of recruitment and follow-up | 6 |
| | 14b | Why the trial ended or was stopped | n.a. |
| Baseline data | 15 | A table showing baseline demographic and clinical characteristics for each group | 12 (Table1) |
| Numbers analysed | 16 | For each group, number of participants (denominator) included in each analysis and whether the analysis was by original assigned groups | See original trial (Becker et al, 2021) |
| Outcomes and estimation | 17a | For each primary and secondary outcome, results for each group, and the estimated effect size and its precision (such as 95% confidence interval) | 16-18 |



For peer review only - http://bmjopen.bmj.com/site/about/guidelines.xhtml

| | | | |
|---|---|---|---|
| | 17b | For binary outcomes, presentation of both absolute and relative effect sizes is recommended | |
| Ancillary analyses | 18 | Results of any other analyses performed, including subgroup analyses and adjusted analyses, distinguishing pre-specified from exploratory | 14-18 |
| Harms | 19 | All important harms or unintended effects in each group (for specific guidance see CONSORT for harms) | n.a. |
| **Discussion** | | | |
| Limitations | 20 | Trial limitations, addressing sources of potential bias, imprecision, and, if relevant, multiplicity of analyses | 26-27 |
| Generalisability | 21 | Generalisability (external validity, applicability) of the trial findings | 26-27 |
| Interpretation | 22 | Interpretation consistent with results, balancing benefits and harms, and considering other relevant evidence | 27 |
| **Other information** | | | |
| Registration | 23 | Registration number and name of trial registry | 4 |
| Protocol | 24 | Where the full trial protocol can be accessed, if available | 7 |
| Funding | 25 | Sources of funding and other support (such as supply of drugs), role of funders | 29-30 |

For peer review only



For peer review only - http://bmjopen.bmj.com/site/about/guidelines.xhtml

BMJ Open

# The occurrence of sensitive topics during ward round: An ancillary analysis of the BEDSIDE-OUTSIDE Trial

| | |
|---|---|
| Journal: | *BMJ Open* |
| Manuscript ID | bmjopen-2023-073584.R1 |
| Article Type: | Original research |
| Date Submitted by the Author: | 27-Jul-2023 |
| Complete List of Authors: | Gross, Sebastian; University Hospital Basel, Medical Communication / Psychosomatic Medicine<br>Becker, Christoph; University Hospital Basel, Medical Communication / Psychosomatic Medicine<br>Beck, Katharina; University Hospital Basel, Medical Communication / Psychosomatic Medicine<br>Memma, Valentina; University Hospital Basel, Medical Communication / Psychosomatic Medicine<br>Gaab, Jens; University of Basel<br>Schütz, Philipp; Kantonsspital Aarau, Endocrinology, Diabetes and Metabolism<br>Leuppi, Jörg; Kantonsspital Baselland Standort Liestal, University Clinic of Medicine<br>Schaefert, Rainer; Universitatsspital Basel, Psychosomatics<br>Langewitz, Wolf Axel ; University Hospital Basle, Psychosomatic Medicine<br>Trendelenburg, Marten; University of Basel<br>Breidthardt, Tobias; Innere Medizin Universitatsspital Basel, Internal Medicine<br>Eckstein, J;  Basel University Hospital<br>Osthoff, Michael; Univ Basel Hosp, Internal Medicine<br>Bassetti, Stefano; University Hospital Basel, Division of Internal Medicine<br>Hunziker, Sabina; University Hospital Basel, Medical Communication / Psychosomatic Medicine |
| <b>Primary Subject Heading</b>: | Communication |
| Secondary Subject Heading: | Evidence based practice |
| Keywords: | Hospitalization, MEDICAL ETHICS, MEDICAL EDUCATION & TRAINING, INTERNAL MEDICINE |
| | |

SCHOLARONE™
Manuscripts

*I, the Submitting Author has the right to grant and does grant on behalf of all authors of the Work (as defined in the below author licence), an exclusive licence and/or a non-exclusive licence for contributions from authors who are: i) UK Crown employees; ii) where BMJ has agreed a CC-BY licence shall apply, and/or iii) in accordance with the terms applicable for US Federal Government officers or employees acting as part of their official duties; on a worldwide, perpetual, irrevocable, royalty-free basis to BMJ Publishing Group Ltd ("BMJ") its licensees and where the relevant Journal is co-owned by BMJ to the co-owners of the Journal, to publish the Work in this journal and any other BMJ products and to exploit all rights, as set out in our licence.*

*The Submitting Author accepts and understands that any supply made under these terms is made by BMJ to the Submitting Author unless you are acting as an employee on behalf of your employer or a postgraduate student of an affiliated institution which is paying any applicable article publishing charge ("APC") for Open Access articles. Where the Submitting Author wishes to make the Work available on an Open Access basis (and intends to pay the relevant APC), the terms of reuse of such Open Access shall be governed by a Creative Commons licence – details of these licences and which Creative Commons licence will apply to this Work are set out in our licence referred to above.*

*Other than as permitted in any relevant BMJ Author's Self Archiving Policies, I confirm this Work has not been accepted for publication elsewhere, is not being considered for publication elsewhere and does not duplicate material already published. I confirm all authors consent to publication of this Work and authorise the granting of this licence.*

For peer review only

# The occurrence of sensitive topics during ward round: An ancillary analysis of the BEDSIDE-OUTSIDE Trial

Sebastian Gross, MSc[1], Christoph Becker, MD[1,2], Katharina Beck, Ph.D.[1], Valentina Memma, MSc[1], Jens Gaab, Ph.D.[4], Philipp Schuetz, MD[3,5], Joerg D. Leuppi, MD[3,6], Rainer Schaefert, MD[1,3,8], Wolf Langewitz, MD[1,3], Marten Trendelenburg, MD[3,7], Tobias Breidthardt, MD[3,7], Jens Eckstein, MD[3,7], Michael Osthoff, MD[3,7], Stefano Bassetti, MD[3,7] and Sabina Hunziker, MD[1,7]

[1]Medical Communication, Department of Psychosomatic Medicine, University Hospital Basel, Basel, Switzerland

[2]Emergency Department, University Hospital Basel, Basel, Switzerland

[3]Faculty of Medicine, University of Basel, Switzerland

[4] Division of Clinical Psychology and Psychotherapy, Faculty of Psychology, University of Basel, Basel, Switzerland

[5]Division of Internal Medicine, Kantonsspital Aarau, Aarau, Switzerland

[6]University Center of Internal Medicine, Kantonsspital Baselland, Liestal, Switzerland

[7]Division of Internal Medicine, University Hospital Basel, Basel, Switzerland

[8]Department of Psychosomatics and Psychiatry, Bethesda Hospital, Basel, Switzerland

**Corresponding author:**

Prof. Dr. med. Sabina Hunziker, MD, MPH



For peer review only - http://bmjopen.bmj.com/site/about/guidelines.xhtml

University Hospital Basel

Petersgraben 4

CH - 4031 Basel

Switzerland

Email: sabina.hunziker@usb.ch

**Word count**

**Abstract: 283**

**Main text: 3323**

For peer review only

## Abstract

**Objective:** Discussing sensitive topics (e.g., medical uncertainty, social issues, non-adherence) during ward rounds is challenging and may negatively impact patient satisfaction with the health care they are receiving. In the previous multicentre randomised BEDSIDE-OUTSIDE trial focusing on communication during ward rounds, we investigated the interplay between sensitive topics and low reported satisfaction with care.

**Design:** Pre-planned secondary analysis of a randomised controlled trial. For this analysis data of the original trial was pooled across intervention groups.

**Setting**: Three Swiss teaching hospitals

**Participants**: Adult patients hospitalized for medical care

**Interventions:** We analysed predefined sensitive health topics and specific elements of communication from audiotapes recorded during ward rounds, for both patients dealing with and without sensitive topics.

**Primary and secondary outcome measures:** The primary endpoint was overall patient satisfaction with care; measured on a visual analogue scale from 0 to 100. Secondary endpoints included duration of ward rounds and further satisfaction outcomes.

**Results:** Of the 919 included patients, 474 had at least one sensitive topic including medical uncertainty (n=251), mental comorbidities (n=137), tumour diagnosis (n=137), and social issues (n= 125). Compared to patients without sensitive topics, patients with sensitive topics reported lower satisfaction with care (mean (SD), 87.7 [±14.6] versus 90.2 [±12.1], adjusted difference -2.5 [95%CI -4.28 to -0.72], p=0.006. Among patients with sensitive topics, risk factors for low satisfaction included several



For peer review only - http://bmjopen.bmj.com/site/about/guidelines.xhtml

parameters concerning patient-physician interaction such as disagreements during ward rounds (mean (SD), 14/212 [6.6%] versus 41/254 [16.1%], adjusted OR 2.78 [95%CI 1.47 to 5.27], p=0.002).

**Conclusions**: A large proportion of medical inpatients must deal with sensitive health topics. This is associated with lower satisfaction with care, particularly if the patient perceives the interaction with doctors during ward rounds as unsatisfactory. Educating physicians on specific communication techniques may help improve care for these patients.

**Trial registration**: clinicaltrials.gov; NCT03210987

## Strengths and limitations

- We investigated a large sample of a broad general medicine population from multiple centres.
- Only Swiss teaching hospitals were included, which might cause selection bias and limit the generalisability of the findings.
- Ward rounds of clinical practice procedures in the three participating hospitals were not standardised to ensure external validity – which may therefore reduce the internal validity.
- The questionnaire used to assess patients' perceptions has not yet been validated.
- As there is no universal definition for sensitive topics, different pre-defined sensitive topics have been pooled into a combined variable, which is a limitation of this study.

For peer review only



For peer review only - http://bmjopen.bmj.com/site/about/guidelines.xhtml

## Introduction

Life threatening disease, conflict with patients, patient non-adherence, psychiatric comorbidities, substance abuse, medical uncertainty among others are often considered to be sensitive topics. However, most research in this regard was mainly qualitative.[1] There is important work about the experiences of patients regarding bedside shift suggesting that asking patient consent, discussing potential critical issues and the degree of involvement preferred at hospital admission is strongly recommended.[2] Also, one quantitative analysis of mixed pediatric and adult patients including 622 ward round discussions found that social issues were addressed in 52%.[3] Thus further research is needed to understand frequency and implications of discussing sensitive topics during ward rounds in clinical practice. Sensitive topics may be important to address as they could affect the patient's well-being and include relevant health care information. On the other hand, the sharing of personal and sensitive information during clinical ward rounds in a non-private setting could potentially offend patients who interpret this as a breach of doctor-patient confidentiality.[4] Physicians may avoid addressing sensitive topics e.g. by terminating problematic conversations, withholding frank responses, downplaying the patient's expressed emotions, or inadequately acknowledging the sentiment underlying the patient's statements.[5, 6]

A previous multicentre randomised trial including 919 internal medicine patients (BEDSIDE-OUTSIDE trial) aimed to compare the impact of patient case presentation during ward rounds on patients' medical knowledge. Patient case presentations were either conducted at the bedside or outside the patient room.[6] Compared with outside the room case presentation, bedside case presentation was shorter and



For peer review only - http://bmjopen.bmj.com/site/about/guidelines.xhtml

resulted in similar patient knowledge, but importantly, sensitive topics were more often avoided and patient confusion was higher. Moreover, an ancillary analysis of the BEDSIDE-OUTSIDE trial focusing on staff satisfaction showed that physicians prefer to discuss sensitive topics outside the room than at the patient's bedside.[7] Despite concerns about addressing sensitive topics with patients, evidence in outpatient settings indicates that discussing these topics may be associated with high satisfaction, positive perceptions of health care, reduced worry, and increased patient participation in treatment decisions in the outpatient setting.[8, 9]

The current study aimed to quantitatively and qualitatively analyse audio tapes from internal medicine ward rounds of patients included in the BEDSIDE-OUTSIDE trial and to determine if discussing sensitive topics in clinical practice is associated with patient satisfaction.

## Material and methods

### Study setting

The current study is a pre-planned secondary analysis of the BEDSIDE-OUTSIDE trial[6] - a pragmatic, investigator-initiated, open-label, multicentre randomised trial conducted in the general medical divisions of three Swiss teaching hospitals (University Hospital Basel, Kantonsspital Aarau, and Kantonsspital Baselland) between July 2017 and October 2019. This report adheres to the CONSORT guidelines[10].



For peer review only - http://bmjopen.bmj.com/site/about/guidelines.xhtml

This analysis studied potential risk factors associated with sensitive topics, the association of sensitive topics with different outcomes, and potential risk factors for low or high levels of patient satisfaction with care. We also provided a qualitative overview of sensitive topic discussions during ward rounds. Because, to our knowledge, there is no well accepted definition of sensitive topics, we defined “sensitive topics” based on the clinical experience of the physician–researcher team and by reviewing previous literature. Sensitive topics were coded prospectively as a situation where at least one of the follow topics was discussed with the patient during the ward round: medical uncertainty, psychiatric comorbidities, tumour diagnosis, social issues, non-adherence, previous conflicts between patient and treating team, and treatment failure.

## Original study population

Newly admitted adult inpatients on medical wards expecting their first weekly ward round consultation were approached by a member of the study team regarding inclusion. Only one patient per room was eligible and we excluded individuals with cognitive or hearing impairment, those unable to understand the local language, and patients who had previously been included in the study. All provided written informed consent.

## Study design, randomisation, and intervention of the original study

Patients were randomised to either the “bedside group” or the “outside the room group” in a 1:1 ratio. In line with current practice, ward rounds for both groups followed the standard practices of each participating hospital.



For peer review only - http://bmjopen.bmj.com/site/about/guidelines.xhtml

Details of the study intervention and a detailed description of the ward round procedure have been reported earlier.[6] In brief, for the purpose of standardization and in line with current practice in Switzerland, the ward round followed the routine medical ward round procedures in both groups, with defined roles of physicians and nurses per usual practice in each participating hospital. In the bedside presentation group, case presentations and discussions occurred only at the bedside in front of the patient, including clinical examination as appropriate, with no discussions beforehand. In the outside the room group, case presentation and discussions were primarily held in the hallway outside the room without the patient present. Afterwards, the team entered the room and gave the patient a short summary of the medical situation, completed the gathering of medical information, examined the patient as needed, and discussed the next steps. Patients, study coordinators, and treating clinicians were not blinded to the allocation. However, study investigators involved in a patient's outcome assessment were blinded to trial allocation.

**Data collection**

Data collection was conducted at different points in time. Baseline patient data was collected before the ward round. During the ward rounds, which were conducted between 9 and 11 a.m., an observer from the research team was present to document timing (i.e., the duration of the ward round allocated per patient). All visits were recorded with an Apple iPad with the device-internal 'Voice Memo' software and rated afterwards by the research team. This approach allowed for the coding of various predefined sensitive topics and elements of communication. After the ward rounds, a second blinded member of the research team interviewed participating



For peer review only - http://bmjopen.bmj.com/site/about/guidelines.xhtml

patients using a standardised questionnaire. All case report forms and items have been described in the original trial.[6]

**Patient and public involvement**

A total of 25 patients hospitalised on the medical wards of the University Hospital of Basel, as well as 15 healthcare providers (physicians and nurses) with experience in daily medical practice and specially in medical ward rounds were involved in the design of the study and intervention of the main paper[6]. To design the trial, patients and healthcare providers helped us in prioritisation and selection of outcomes. Patients were asked for priority focus of this study. To design the intervention for the main trial, input was sought from patients and healthcare providers.

**Outcome measures**

*Baseline factors and predictors*

We assessed patient baseline characteristics including age, sex, number of children, family status, citizenship, level of education, occupation, main diagnosis, and comorbidities. Predictors for the occurrence of sensitive topics included patients' quality of life assessed with the validated EuroQuol EQ-5D questionnaire[11], patient's Decisional Control Preference[12, 13], application of specific patient-centred communication techniques (WEMS: waiting, echoing, mirroring, summarising; NURSE: naming, understanding, respecting, supporting and exploring [14, 15], as well as the following general communication factors rated by the study team: information given regarding diagnosis, symptoms, treatment steps, social issues; talking “about” instead of “with” the patient; communicating at cross purposes. Furthermore, moments were identified, when raters had the impression that a patient



For peer review only - http://bmjopen.bmj.com/site/about/guidelines.xhtml

did not understand information but did not ask more questions; instances when the physician explicitly disagreed with or corrected a patient; when a patient disagreed with / corrected a physician; occurrence of Current Unvoiced Elements [*cues*] and concerns; addressing *cue* and concerns; and replying by providing information instead of exploration. *Cues* and concerns were defined as proposed by previous research.[16] In short, a *cue* or concern included a verbally or non-verbally expressed hint expressed by the patient that might have a certain subjective importance and a negative emotional impact.

*Primary and secondary endpoints*

The primary endpoint for this analysis was patients' overall satisfaction, defined by the mean of several satisfaction measures (see secondary endpoints) and measured on a visual analogue scale (VAS) of 0-100, with 0 indicating the lowest and 100 the highest possible satisfaction. For the purpose of this study, patient satisfaction below the median were defined as low satisfaction. Secondary endpoints included satisfaction outcomes for: ward rounds, hospital stay, medical care, physician communication, and nursing team communication, measured on the same a visual analogue scale (VAS) from 0-100.

We also assessed three elements of patients' subjective and objective knowledge of their own medical situation: the understanding of the disease, therapeutic approach, and further plans for care. Each dimension was rated by the patient on a visual analogue scale from 0 to 100 (0 "no knowledge about the situation" to 100 "best possible knowledge about the situation"). Subjective knowledge was defined as patient's self-assessment of being informed and was rated during a structured interview in the afternoon after the ward round. The study team rated objective



For peer review only - http://bmjopen.bmj.com/site/about/guidelines.xhtml

knowledge by comparing patients' recall of information about their main disease with medical information from the chart.

Ward round duration was measured in minutes and included: total time during the ward round, individual outside the room discussions or bedside discussions, and debriefing outside the room.

Further secondary endpoints included multiple items relating to patients' perception of: time spent on the ward round, patients' discomfort during the ward round, physician behaviour during the ward round, and general quality of care (**Supplement Tables**). All were rated in the structured interview after the ward round and measured on a visual analogue scale from 0 to 100.

**Statistical analysis**

Baseline parameters and outcomes were stratified among patients with and without sensitive topics. Logistic regression analyses were conducted to evaluate factors associated with sensitive topics. Furthermore, univariable linear and logistic regression models were conducted to investigate associations of sensitive topics with primary and secondary endpoints. We additionally calculated multivariable models adjusted for study centre and randomisation arm (bedside or hallway). In the subgroup of patients with sensitive topics, potential risk factors for low satisfaction compared to high satisfaction were assessed using Student's t-test. We used STATA 15.0 (Stata Corp., College Station, TX, USA) for all statistical analyses. A p-value of <0.05 (two-tailed) was considered statistically significant. STATA 15.0 (Stata Corp., College Station, TX, USA) was used for all analyses.



For peer review only - http://bmjopen.bmj.com/site/about/guidelines.xhtml

# Results

## Study flow of the original trial

Of 1441 patients approached for inclusion in the original trial, 1092 patients agreed and gave written informed consent. After exclusions, 919 patients were included in the final analysis.[6]

## Baseline characteristics (Table 1 and Supplement Table 1)

A total of 474 (51.6%) patients had at least one sensitive topic needing discussion during the ward round. In total, 791 sensitive topics emerged during ward round discussions, including: medical uncertainty (n = 251), psychiatric comorbidities (n = 161), tumour diagnosis (n = 137), social issues (n = 125), non-adherence (n = 43), previous conflicts between patient and treating team (n = 38), and treatment failure (n = 36) (**Figure 1**).

For peer review only

**Table 1. Associations of patient characteristics with sensitive topics**

| | n | All | No sensitive topics | Sensitive topics | *p-value* | OR (95%CI)* | *p-value* |
|---|---|---|---|---|---|---|---|
| **n** | | | 445 | 474 | | | |
| **Sociodemographic factors** | | | | | | | |
| Age, years; mean (SD) | 919 | 65.0 (15.9) | 65.7 (15.9) | 64.3 (15.9) | *0.16* | 1 (0.99, 1) | *0.304* |
| Female sex, n (%) | 919 | 361 (39.3%) | 173 (38.9%) | 188 (39.7%) | *0.81* | 1.05 (0.8, 1.37) | *0.741* |
| Number of children, mean (SD) | 919 | 2.5 (9.2) | 2.1 (6.7) | 2.8 (11.0) | *0.26* | 1.01 (0.99, 1.02) | *0.365* |
| **Family status** | 919 | | | | | | |
| Single, relationship, married, registered partnership, n (%) | | 648 (70.5%) | 317 (71.2%) | 331 (69.8%) | *0.84* | | |
| Separated, divorced, n (%) | | 147 (16.0%) | 68 (15.3%) | 79 (16.7%) | | 1.05 (0.73, 1.52) | *0.787* |
| Widowed, n (%) | | 124 (13.5%) | 60 (13.5%) | 64 (13.5%) | | 1.07 (0.72, 1.59) | *0.738* |
| **Citizenship** | 919 | | | | | | |
| Switzerland, n (%) | | 789 (85.9%) | 386 (86.7%) | 403 (85.0%) | *0.71* | | |
| Germany, n (%) | | 55 (6.0%) | 26 (5.8%) | 29 (6.1%) | | 0.97 (0.55, 1.68) | *0.904* |
| Other, n (%) | | 75 (8.2%) | 33 (7.4%) | 42 (8.9%) | | 1.22 (0.75, 1.98) | *0.421* |
| **Occupation** | 919 | | | | | | |
| Employed / working + IV, n (%) | | 259 (28.2%) | 129 (29.0%) | 130 (27.4%) | ***0.041*** | | |
| Unemployed / homemaker, n (%) | | 39 (4.2%) | 13 (2.9%) | 26 (5.5%) | | 2 (0.97, 4.09) | *0.059* |
| Retired / IV support, n (%) | | 605 (65.8%) | 296 (66.5%) | 309 (65.2%) | | 1.05 (0.78, 1.42) | *0.735* |
| In education, n (%) | | 6 (0.7%) | 5 (1.1%) | 1 (0.2%) | | 0.19 (0.02, 1.63) | *0.128* |
| Other, n (%) | | 10 (1.1%) | 2 (0.4%) | 8 (1.7%) | | 3.41 (0.7, 16.56) | *0.128* |
| **Comorbidities** | 919 | | | | | | |
| Charlson Comorbidity Index, mean (SD) | | 4.45 (2.88) | 4.4 (2.8) | 4.5 (3.0) | *0.54* | 0.76 (0.59, 1) | ***0.048*** |
| Cardiology, n (%) | | 485 (52.8%) | 248 (55.7%) | 237 (50.0%) | *0.082* | 0.86 (0.65, 1.13) | *0.273* |
| Neurology, n (%) | | 193 (21.0%) | 80 (18.0%) | 113 (23.8%) | ***0.029*** | 1.01 (0.77, 1.33) | *0.941* |
| Rheumatology/Immunology, n (%) | | 143 (15.6%) | 68 (15.3%) | 75 (15.8%) | *0.82* | 1.03 (0.78, 1.37) | *0.819* |
| Gastrointestinal, n (%) | | 254 (27.6%) | 118 (26.5%) | 136 (28.7%) | *0.46* | 1.18 (0.85, 1.65) | *0.325* |



For peer review only - http://bmjopen.bmj.com/site/about/guidelines.xhtml

| | | | | | | | | |
|---|---|---|---|---|---|---|---|---|
| Endocrinology, n (%) | | 352 (38.3%) | 185 (41.6%) | 167 (35.2%) | ***0.048*** | 1.21 (0.86, 1.71) | *0.264* |
| Respiratory, n (%) | | 315 (34.3%) | 152 (34.2%) | 163 (34.4%) | *0.94* | 1.07 (0.81, 1.4) | *0.627* |
| Infectious diseases, n (%) | | 171 (18.6%) | 77 (17.3%) | 94 (19.8%) | *0.33* | 1.12 (0.85, 1.48) | *0.416* |
| Renal, n (%) | | 317 (34.5%) | 150 (33.7%) | 167 (35.2%) | *0.63* | 0.25 (0.07, 0.91) | ***0.035*** |
| Gynecology, n (%) | | 14 (1.5%) | 11 (2.5%) | 3 (0.6%) | ***0.023*** | 0.26 (0.07, 0.94) | ***0.04*** |
| Urology, n (%) | | 93 (10.1%) | 43 (9.7%) | 50 (10.5%) | *0.66* | 1.24 (0.8, 1.93) | *0.338* |
| Oncology, n (%) | | 287 (31.2%) | 145 (32.6%) | 142 (30.0%) | *0.39* | 1.81 (1.13, 2.9) | ***0.014*** |
| Psychiatry, n (%) | | 104 (11.3%) | 31 (7.0%) | 73 (15.4%) | ***<0.001*** | 1.97 (1.21, 3.2) | ***0.006*** |
| Depression, n (%) | | 82 (8.9%) | 29 (6.5%) | 53 (11.2%) | ***0.013*** | 0.89 (0.68, 1.15) | *0.364* |
| Other, n (%) | | 498 (54.2%) | 248 (55.7%) | 250 (52.7%) | *0.36* | 0.95 (0.73, 1.25) | *0.734* |
| Health self-rating VAS [0-100], mean (SD) | 856 | 56.9 (23.0) | 58.7 (22.5) | 55.1 (23.3) | ***0.021*** | 0.99 (0.99, 1) | ***0.018*** |
| Quality of life (EQ-5D) Index, mean (SD) | 889 | | 0.749 (0.278) | 0.671 (0.301) | ***<0.001*** | 0.42 (0.26, 0.67) | ***<0.001*** |

**adjusted for study centre, intervention. Odds ratios were calculated with logistic regression models. Abbreviations: OR, odds ratio; SD, standard deviation; CI, confidence interval; n, number; IV, Swiss disability insurance*



For peer review only - http://bmjopen.bmj.com/site/about/guidelines.xhtml

The mean age was 65 years ± 15.9 years (SD). Patients with and without sensitive topics were of comparable age. Overall, 39.3% (n = 361) of patients were female with no differences regarding the occurrence of sensitive topics to that of males. Patients' number of children, family status, citizenship, level of education, and occupation were not associated with the presence of sensitive topics. Individuals with psychiatric comorbidities, however, were more likely to have sensitive topics present (73/474 [15.4%] versus 31/445 [7.0%], $p < 0.001$; adjusted OR 1.97, 95% CI 1.21 to 3.2; $p = 0.006$). Quality of life regarding mobility, self-care, usual activities, pain / discomfort, and anxiety / depression was perceived lower by patients with sensitive topics; consequently resulting in a lower EQ-5D quality of life index score.

**Communication-related factors (Table 2)**

Patients with sensitive health topics provided more *cue*s (79/474 [16.7%] versus 32/445 [7.2%], $p < 0.001$; adjusted OR 2.36, 95%CI 1.52 to 3.66; $p < 0.001$) and *cue*s were more often addressed by physicians in patients with sensitive topics (40/474 [8.4%] versus 19/445 [4.3%], $p = 0.01$, adjusted OR 1.83, 95% CI 1.03 to 3.24; $p = 0.039$). In the subgroup of patients who mentioned emotions, physicians applied patient-centred communication techniques (i.e., NURSE, WEMS) similarly among individuals with and without sensitive topics (**Supplement Table 2**).



For peer review only - http://bmjopen.bmj.com/site/about/guidelines.xhtml

## Table 2. Associations of communication factors with sensitive topics

| | n | No sensitive topics | Sensitive topics | *p-value* | Adjusted OR (95%CI)* | *p-value* |
|---|---|---|---|---|---|---|
| | | 445 | 474 | | | |
| **Communication factors** | 919 | | | | | |
| Visible evidence of emotion, n (%) | | 48 (10.8%) | 70 (14.8%) | *0.071* | 1.35 (0.9, 2.01) | *0.144* |
| Medical staff talks to patient about diagnosis, n (%) | | 215 (48.3%) | 271 (57.2%) | ***0.007*** | 1.59 (1.21, 2.09) | ***0.001*** |
| Medical staff talks to patient about symptoms, n (%) | | 399 (89.7%) | 443 (93.5%) | ***0.038*** | 1.66 (1.02, 2.69) | ***0.041*** |
| Medical staff talks to patient about treatment, n (%) | | 334 (75.1%) | 383 (80.8%) | ***0.036*** | 1.38 (1, 1.9) | ***0.049*** |
| Medical staff talks to patient about next steps, n (%) | | 344 (77.3%) | 431 (90.9%) | ***<0.001*** | 2.8 (1.89, 4.13) | ***<0.001*** |
| Medical staff talks to patient about social issues, n (%) | | 272 (61.1%) | 320 (67.5%) | ***0.043*** | 1.43 (1.08, 1.9) | ***0.013*** |
| Medical staff talks about patient instead of with patient after case discussion, n (%) | | 329 (73.9%) | 351 (74.1%) | *0.97* | 1.19 (0.87, 1.62) | *0.282* |
| Physician and patient talk at cross purposes, n (%) | | 17 (3.8%) | 35 (7.4%) | ***0.019*** | 2.29 (1.24, 4.25) | ***0.008*** |
| Patient seems not to understand something, but does not ask, n (%) | | 6 (1.3%) | 17 (3.6%) | ***0.030*** | 4.15 (1.55, 11.08) | ***0.005*** |
| Physician disagrees with / corrects patient, n (%) | | 30 (6.7%) | 57 (12.0%) | ***0.006*** | 2.05 (1.28, 3.3) | ***0.003*** |
| Patient disagrees with / corrects physician, n (%) | | 27 (6.1%) | 53 (11.2%) | ***0.006*** | 2.12 (1.29, 3.48) | ***0.003*** |
| Occurrence of "*cue*", n (%) | | 32 (7.2%) | 79 (16.7%) | ***<0.001*** | 2.36 (1.52, 3.66) | ***<0.001*** |
| Addressing "*cue*" concern, n (%) | | 19 (4.3%) | 40 (8.4%) | ***0.010*** | 1.83 (1.03, 3.24) | ***0.039*** |
| Reacts by providing information instead of exploration, n (%) | | 19 (4.3%) | 33 (7.0%) | *0.077* | 1.59 (0.88, 2.87) | *0.122* |

**adjusted for study centre, intervention. Odds ratios were calculated with logistic regression models. Abbreviations: OR, odds ratio; CI, confidence interval; n, number; cue, current unvoiced element*

For peer review only

**Primary and secondary outcomes (Table 3)**

Patients with sensitive topics reported lower overall satisfaction (87.7 ± 14.6 versus 90.2 ± 12.1, p = 0.006; adjusted difference -2.5, 95%CI -4.28 to -0.72; p = 0.006). Sensitive topics were associated with less subjective knowledge (77.8 ± 22 versus 81.2 ± 19, p = 0.013; adjusted difference -3.86, 95% CI -6.57 to -1.15; p = 0.005) as well as objective knowledge (68.7 ± 25.8 versus 72.6 ± 24.9, p = 0.021; adjusted difference -3.83, 95% CI -7.18 to -0.48; p = 0.025).



For peer review only - http://bmjopen.bmj.com/site/about/guidelines.xhtml

## Table 3. Association of sensitive topics with various outcomes

| | n | No sensitive topics | Sensitive topics | *p-value* | Adjusted difference or OR (95%CI)* | *p-value* |
|---|---|---|---|---|---|---|
| **Primary endpoint** | | 445 | 474 | | | |
| Overall satisfaction (VAS 0-100), mean (SD) | 906 | 90.2 (12.1) | 87.7 (14.6) | ***0.006*** | -2.5 (-4.28, -0.72) | ***0.006*** |
| **Patient knowledge about medical care** | | | | | | |
| Average subjective knowledge about their medical care, mean (SD) | 919 | 81.2 (19.0) | 77.8 (22.0) | ***0.013*** | -3.86 (-6.57, -1.15) | ***0.005*** |
| Average objective knowledge about their medical care, mean (SD) | 919 | 72.6 (24.9) | 68.7 (25.8) | ***0.021*** | -3.83 (-7.18, -0.48) | ***0.025*** |
| **Measured timeliness of ward round** | | | | | | |
| Duration of outside the room discussions (min), mean (SD) | 919 | 2.8 (3.6) | 4.9 (5.0) | ***<0.001*** | 1.41 (1.02, 1.81) | ***<0.001*** |
| Duration of bedside discussions (min), mean (SD) | 919 | 8.2 (4.2) | 9.0 (5.0) | ***0.005*** | 1.56 (1.03, 2.09) | ***<0.001*** |
| Duration of debriefing outside the room (min), mean (SD) | 919 | 0.4 (1.1) | 0.5 (1.1) | *0.063* | 0.16 (0.01, 0.31) | ***0.038*** |
| Total duration of ward round per patient (min), mean (SD) | 919 | 11.3 (4.6) | 14.5 (5.6) | ***<0.001*** | 3.13 (2.47, 3.79) | ***<0.001*** |
| **Patient perception regarding discomfort during the ward round** | | | | | | |
| Medical terms used during ward round were confusing (VAS 0-100), mean (SD) | 853 | 15.9 (28.2) | 19.2 (30.4) | *0.099* | 3.89 (-0.07, 7.85) | *0.054* |
| Ward round discussions made me worry (VAS 0-100), mean (SD) | 871 | 6.6 (25.7) | 11.7 (24.8) | ***0.003*** | 4.82 (1.4, 8.24) | ***0.006*** |
| I felt uncomfortable during ward round (VAS 0-100), mean (SD) | 870 | 4.5 (15.6) | 8.2 (21.6) | ***0.005*** | 3.2 (0.64, 5.76) | ***0.014*** |
| Ward round discussions unsettled me (VAS 0-100), mean (SD) | 752 | 4.4 (15.9) | 7.2 (19.9) | ***0.033*** | 2.84 (0.22, 5.45) | ***0.034*** |
| **Patient perception regarding physician`s behaviour during the ward round** | | | | | | |
| Physicians treated me with respect (VAS 0-100), mean (SD) | 867 | 97.4 (8.8) | 95.5 (13.1) | ***0.011*** | -2.05 (-3.57, -0.53) | ***0.008*** |
| I was taken seriously (VAS 0-100), mean (SD) | 861 | 96.1 (10.9) | 94.0 (15.6) | ***0.021*** | -2.17 (-4.01, -0.32) | ***0.021*** |
| Physicians respected my privacy (VAS 0-100), mean (SD) | 794 | 89.7 (24.8) | 91.1 (20.9) | *0.38* | 1.41 (-1.85, 4.66) | *0.396* |
| Physicians showed compassion (VAS 0-100), mean (SD) | 693 | 81.0 (28.4) | 75.2 (33.5) | ***0.014*** | -5.56 (-10.33, -0.8) | ***0.022*** |
| My issues were dealt with discreetly (VAS 0-100), mean (SD) | 764 | 88.3 (23.7) | 89.2 (21.1) | *0.55* | 1.33 (-1.91, 4.57) | *0.42* |
| Some topics during ward round communication caused inconvenience (VAS 0-100), mean (SD) | 853 | 4.0 (15.4) | 7.6 (21.8) | ***0.005*** | 3.58 (0.99, 6.17) | ***0.007*** |

**adjusted for study centre, intervention. All differences calculated with linear regression models for continuous data. Odds ratios were calculated with logistic regression models. Abbreviations: OR, odds ratio; SD, standard deviation; CI, confidence interval; VAS, visual analogue scale; min, minutes*



For peer review only - http://bmjopen.bmj.com/site/about/guidelines.xhtml

Duration of outside the room discussions, bedside discussions, and debriefings was longer in patients with sensitive topics. Mean (±SD) duration (min) of ward round per patient was 14.5 ± 5.6 versus 11.3 ± 4.6, $p < 0.001$; adjusted difference 3.13, 95% CI 2.47 to 3.79; $p < 0.001$. Compared to patients without sensitive topics, patients with sensitive topics felt more uncomfortable (4.5 ± 15.6 versus 8.2 ± 21.6, p = 0.005; adjusted difference 3.2, 95%CI 0.64 to 5.76; p = 0.014) and unsettled (4.4 ± 15.9 versus 7.2 ± 19.9, p = 0.033; adjusted difference 2.84, 95%CI 0.22 to 5.45; p = 0.034) during the ward round, and discussions causing them to worry (6.6 ± 25.7 versus 11.7 ± 24.8, p = 0.003; adjusted difference 4.82, 95% CI 1.4 to 8.24; p = 0.006). They also felt less confident with the physician team (91.9 ±14.4 versus 89.5 ± 16.8, p = 0.02; adjusted difference -2.52, 95%CI -4.65 to -0.4; p = 0.02), whereas there were no significant differences in confidence with the nursing team (**Supplement Table 3**).

Patients with sensitive topics perceived physicians less compassionate and also felt less respected, taken seriously, and more uneasy (**Table 3**).

**Risk factors for low satisfaction among patients with sensitive topics (Table 4)**

In a further step, we investigated factors associated with low satisfaction among patients with sensitive topics **(Table 4).** Several factors were associated with low satisfaction, such as parameters regarding the patient-physician interaction during ward rounds such as physicians disagreeing with patients (14/212 [6.6%] versus 41/254 [16.1%], adjusted OR 2.78,95%CI 1.47 to 5.27; p=0.002), and patients disagreeing with physicians (14/212 [6.6%] versus 37/254 [14.6%], adjusted OR 2.42, 95%CI 1.27 to 4.61; p=0.007). Further factors were a patient's lack of knowledge about their own medical care (both subjective and objective knowledge),



For peer review only - http://bmjopen.bmj.com/site/about/guidelines.xhtml

an overall longer duration of the ward round, reduced capacity to understand the main disease, the implemented therapeutic measure and further plans of care, as well as other physician-patient interactions (e.g., respectful treatment, physician compassion, observing privacy), see also **Supplement Table 4**.

For peer review only



For peer review only - http://bmjopen.bmj.com/site/about/guidelines.xhtml

**Table 4. Risk factors for low satisfaction among patients with sensitive topics**

| | n | high satisfaction | low satisfaction | *p-value* | adjusted difference or OR (95%CI)* | *p-value* |
|---|---|---|---|---|---|---|
| | | 212 | 254 | | | |
| **Other communication factors** | 466 | | | | | |
| Physician disagrees with/corrects patient, n (%) | | 14 (6.6%) | 41 (16.1%) | ***0.001*** | *2.78 (1.47, 5.27)* | ***0.002*** |
| Patient disagrees with/corrects physician, n (%) | | 14 (6.6%) | 37 (14.6%) | ***0.006*** | *2.42 (1.27, 4.61)* | ***0.007*** |
| **Patient knowledge about medical care** | 466 | | | | | |
| Average subjective knowledge about their medical care, mean (SD) | | 87.0 (16.2) | 70.7 (22.9) | ***<0.001*** | 0.95 (0.94, 0.97) | ***<0.001*** |
| Average objective knowledge about their medical care, mean (SD) | | 71.7 (25.3) | 66.2 (26.0) | ***0.023*** | 0.99 (0.98, 1) | ***0.027*** |
| **Measured timeliness of ward round** | 466 | | | | | |
| Total duration of ward round per patient (min), mean (SD) | | 13.9 (5.6) | 15.1 (5.6) | ***0.019*** | 1.05 (1.01, 1.08) | ***0.01*** |
| **Patient perception regarding time spent on ward round** | 466 | | | | | |
| Overall duration of ward round was sufficient (VAS 0-100), mean (SD) | | 95.78 (15.38) | 83.81 (24.55) | ***<0.001*** | 0.96 (0.95, 0.98) | ***<0.001*** |
| Time spent with physicians was sufficient (VAS 0-100), mean (SD) | | 96.71 (10.02) | 80.66 (22.53) | ***<0.001*** | 0.92 (0.9, 0.94) | ***<0.001*** |
| Patient estimation of time spent with patient on ward round (min), mean (SD) | | 12.18 (7.18) | 11.26 (6.58) | *0.15* | 0.98 (0.96, 1.01) | *0.176* |
| Patient estimation of time spent per day with patient case overall (min), mean (SD) | | 82.09 (86.72) | 66.73 (66.44) | ***0.031*** | 1 (0.99, 1) | ***0.035*** |
| **The ward round was helpful for better** | | | | | | |
| - understanding the main illness (VAS 0-100), mean (SD) | | 75.03 (32.91) | 54.64 (33.95) | ***<0.001*** | 0.98 (0.98, 0.99) | ***<0.001*** |
| - further therapeutic measures (VAS 0-100), mean (SD) | | 74.14 (32.45) | 53.03 (33.52) | ***<0.001*** | 0.98 (0.97, 0.99) | ***<0.001*** |
| - further plans of care (VAS 0-100), mean (SD) | | 83.33 (69.99) | 62.12 (52.73) | ***<0.001*** | 0.99 (0.98, 0.99) | ***<0.001*** |
| All my questions were answered (VAS 0-100), mean (SD) | | 97.05 (8.55) | 82.10 (24.16) | ***<0.001*** | 0.92 (0.9, 0.94) | ***<0.001*** |
| I was able to understand all answers to my questions (VAS 0-100), mean (SD) | | 96.84 (10.74) | 85.85 (20.14) | ***<0.001*** | 0.93 (0.91, 0.95) | ***<0.001*** |
| Information during visit has been adequate, n(%) | | 197 (92.9%) | 176 (69.3%) | ***<0.001*** | 0.17 (0.1, 0.31) | ***<0.001*** |
| Patient did not understand something during the round, n(%) | | 13 (6.1%) | 36 (14.2%) | ***0.005*** | 2.58 (1.32, 5.02) | ***0.005*** |
| Estimation of my participation in the discussion (VAS 0-100), mean (SD) | | 67.80 (30.61) | 51.78 (29.76) | ***<0.001*** | 0.98 (0.98, 0.99) | ***<0.001*** |
| Disease was explained in an understandable way (VAS 0-100), mean (SD) | | 91.95 (19.46) | 76.61 (27.1) | ***<0.001*** | 0.97 (0.96, 0.98) | ***<0.001*** |
| Treatment was explained in an understandable way (VAS 0-100), mean (SD) | | 91.32 (20.51) | 69.49 (31.19) | ***<0.001*** | 0.96 (0.95, 0.97) | ***<0.001*** |
| Upcoming examinations were explained in an understandable way (VAS 0-100), mean (SD) | | 91.64 (19.12) | 74.34 (28.26) | ***<0.001*** | 0.96 (0.95, 0.97) | ***<0.001*** |
| The information given during the visit was clear and understandable (VAS 0-100), mean (SD) | | 97.86 (9.04) | 82.92 (22.92) | ***<0.001*** | 0.9 (0.88, 0.93) | ***<0.001*** |
| **Patient perception regarding discomfort during the ward round** | 466 | | | | | |
| Medical terms used during ward round were confusing (VAS 0-100), mean (SD) | | 14.1 (27.87) | 23.13 (30.42) | ***0.003*** | 1.01 (1, 1.02) | ***0.003*** |
| Ward round discussions made me worry (VAS 0-100), mean (SD) | | 7.93 (22.02) | 15.86 (25.73) | ***<0.001*** | 1.02 (1.01, 1.02) | ***0.001*** |



For peer review only - http://bmjopen.bmj.com/site/about/guidelines.xhtml

| | | | | | | |
|---|---|---|---|---|---|---|
| I felt uncomfortable during ward round (VAS 0-100), mean (SD) | | 3.8 (14.88) | 11.98 (24.44) | ***<0.001*** | 1.02 (1.01, 1.04) | ***<0.001*** |
| Ward round discussions unsettled me (VAS 0-100), mean (SD) | | 2.23 (9.31) | 12.01 (22.3) | ***<0.001*** | 1.06 (1.03, 1.08) | ***<0.001*** |
| **Patient perception regarding physician`s behaviour during the ward round** | | | | | | |
| Physicians treated me with respect (VAS 0-100), mean (SD) | 466 | 99.31 (3.02) | 91.87 (16.37) | ***<0.001*** | 0.85 (0.8, 0.9) | ***<0.001*** |
| I was taken seriously (VAS 0-100), mean (SD) | 466 | 98.82 (4.97) | 88.87 (19.54) | ***<0.001*** | 0.89 (0.85, 0.92) | ***<0.001*** |
| Physicians respected my privacy (VAS 0-100), mean (SD) | 466 | 95.83 (14.64) | 86.64 (21.55) | ***<0.001*** | 0.96 (0.94, 0.98) | ***<0.001*** |
| Physicians showed compassion (VAS 0-100), mean (SD) | 466 | 83.98 (27.57) | 66.79 (30.85) | ***<0.001*** | 0.98 (0.97, 0.99) | ***<0.001*** |
| My issues were dealt with discreetly (VAS 0-100), mean (SD) | 466 | 95.01 (14.75) | 83.95 (21.16) | ***<0.001*** | 0.95 (0.94, 0.97) | ***<0.001*** |
| Some topics during ward round communication caused inconvenience (VAS 0-100), mean (SD) | 466 | 3.66 (15.99) | 10.88 (23.80) | ***<0.001*** | 1.02 (1.01, 1.03) | ***0.001*** |
| I was encouraged to address personal topics (VAS 0-100), mean (SD) | 466 | 90.57 (21.35) | 76.54 (27.65) | ***<0.001*** | 0.97 (0.96, 0.98) | ***<0.001*** |
| My privacy was violated (VAS 0-100), mean (SD) | 466 | 0.88 (5.30) | 5.19 (15.55) | ***<0.001*** | 1.07 (1.02, 1.12) | ***0.003*** |
| Teaching took place during ward round (yes, %) | 466 | 61 (28.8%) | 65 (25.6%) | *0.44* | 0.87 (0.57, 1.33) | *0.51* |
| - if yes , teaching was perceived as disruptive, (VAS 0-100) mean (SD) | 125 | 0.8 (6.4) | 10.3 (29.3) | ***0.015*** | 1.04 (1, 1.08) | *0.07* |
| **Patients' perception regarding quality of care (VAS 0-100)** | 466 | | | | | |
| I felt "in good hands" in this hospital, mean (SD) | | 96.19 (9.28) | 83.57 (17.04) | ***<0.001*** | 0.9 (0.88, 0.93) | ***<0.001*** |
| I felt there were contradicting statements from physicians and nursing team, mean (SD) | | 7.58 (20.90) | 18.87 (28.23) | ***<0.001*** | 1.02 (1.01, 1.03) | ***<0.001*** |
| I feel confident with the physician team, mean (SD) | | 95.88 (11.54) | 83.79 (18.07) | ***<0.001*** | 0.92 (0.9, 0.94) | ***<0.001*** |
| I feel confident with the nursing team, mean (SD) | | 97.45 (6.46) | 86.91 (15.65) | ***<0.001*** | 0.89 (0.87, 0.92) | ***<0.001*** |
| Physicians and nurses collaborate well, mean (SD) | | 95.68 (9.14) | 84.25 (15.8) | ***<0.001*** | 0.91 (0.89, 0.93) | ***<0.001*** |
| I feel physicians are highly competent to treat the current illness, mean (SD) | | 96.35 (9.71) | 82.84 (18.49) | ***<0.001*** | 0.9 (0.88, 0.93) | ***<0.001*** |
| I feel nurses are highly competent to treat the current illness, mean (SD) | | 96.51 (9.16) | 86.39 (31.79) | ***<0.001*** | 0.93 (0.91, 0.95) | ***<0.001*** |

**adjusted for centrum, intervention. All differences calculated with linear regression models for continuous data. Odds ratios were calculated with logistic regression models. Abbreviations: OR, odds ratio; SD, standard deviation; CI, confidence interval; VAS, visual analogue scale*

For peer review only

## Qualitative analysis

Examples of conversations extracted from audiotapes are provided in **Supplement Table 5**.



For peer review only - http://bmjopen.bmj.com/site/about/guidelines.xhtml

## Discussion

We report several key findings of this ancillary project from a multicentre randomised controlled trial that investigated sensitive topics and specific elements recorded during ward round communication: i) Sensitive topics arose frequently during ward rounds, with more than half of patients reporting at least one specific issue. Participants reporting a sensitive topic were more complex in their treatment and required more attention from medical staff, making ward rounds longer and more demanding compared to individuals without a sensitive topic; ii) Medical uncertainty was the most frequent sensitive topic; iii) The presence of sensitive topics in patients was associated with being less satisfied overall with their care; iv) Risk factors for low satisfaction among patients with sensitive topics included several parameters regarding patient-physician interaction during the ward round (various disagreements) - highlighting the importance of patient-centred communication.

Several results of the analysis provide important information and are worth further discussion. Many issues could benefit from special attention, but often take time and may not fit into the ward round time-schedule. However, it is important to address them, as patients may feel unheard and dissatisfied if these topics are not discussed (see an example of a social issue from qualitative analysis in **Box 1**).

*Patient:* I planned to move in with my partner after my hospital stay. She's a former nurse, but now a relative of hers has died.

*Chief physician:* Oh dear.

*Patient (tearful):* When she heard what could happen to me, she said no. I mean, over the last years I've burdened her with my illness. But now I am really down.

*Chief physician:* Yes, that is… I can understand that.

*Patient:* Now I had to agree with her and promised to look for assisted accommodation.

*Chief physician:* Yes, we can discuss this with our case management team. Or have you already found something?



For peer review only - http://bmjopen.bmj.com/site/about/guidelines.xhtml

*Patient:* No, because I expected to move in with her. But now her relative has passed away and it is all so complicated…

*Chief physician:* Yeah, I understand! But unfortunately, we don't have much time during the ward round so we'll have to discuss your further care on another occasion.

*Patient:* Okay, yes, thank you.

***Box 1:*** *Example of a patient-physician discussion about social burden*

Another frequently encountered sensitive topic concerned tumour diagnosis. Previous literature has shown an association between physicians' communicative competence and its effect on quality of life in cancer patients.[17] As quality of life was also lower in patients with sensitive topics in our study, this again underlines the importance of communication skills when talking about tumour diagnoses.

Psychiatric comorbidities and psychological issues are also perceived as sensitive topics and may be directly linked to physical disease. Physicians often hesitate to address mental health issues during the ward round because of a lack of privacy.[7] However, our results suggest that patients do not feel violated in their privacy when sensitive topics are addressed (see **Supplement Table 3**). Also, previous research has shown that 63% of patients prefer to discuss mental health issues in general, at least in the primary care setting.[18] However, similar data for the inpatient setting with less privacy is currently lacking.

Recent studies highlight that medical uncertainty remains a significant factor in medicine despite the advances in diagnostic possibilities particularly in patients reporting various unspecific symptoms.[19] Thus, during ward rounds, the treating team often discusses possible differential diagnoses leaving the patient potentially with remaining uncertainty about the specific disease. This is in line with our results where medical uncertainty was reported as the most common sensitive topic. Medical uncertainty has been shown to negatively affect patients' physical, and mental, well-



For peer review only - http://bmjopen.bmj.com/site/about/guidelines.xhtml

being.[20] Moreover, dealing with medical uncertainty may also cause anxiety in physicians, negatively affect their work-related satisfaction, and result in substandard care.[21] Guidance on how best to deal with medical uncertainty and how to address it in the patient's presence is warranted to ensure both physician well-being and patient-centred care. Specific communication skills training may help achieve these aims.[19, 22]

Importantly, our study found lower satisfaction in patients with sensitive topics compared to patients without sensitive topics. This may be due to confounding because patients with sensitive topics are often more complex and have different medical needs. Interestingly, previous literature in outpatient settings suggests that adequately addressing sensitive topics is associated with higher satisfaction[8, 9]. Santelli et al. (2019) reported higher levels of confidentiality during the conversation when sensitive topic were discussed.[23] We did not have information about the extend sensitive topics were discussed with patients in our study. The restricted opportunity for confidentiality during ward rounds may provide some barriers here – even though patients did not expressly state that their privacy was violated. Limited time with the physician may be another contributor to the discrepancy in satisfaction between out- and inpatient settings. Especially time constraints during ward rounds might be a challenge to effectively address sensitive topics, compromising patient satisfaction. However, most patients stated that the time was sufficient, and the duration of ward rounds was significantly longer in patients with sensitive topics. Also, we also found the duration of ward rounds to be associated with lower satisfaction in patients with sensitive topics. It would be important to study the effect of addressing sensitive topics in the inpatient population regarding satisfaction with care in an interventional study.



For peer review only - http://bmjopen.bmj.com/site/about/guidelines.xhtml

Over the last decades, medicine has shifted from a paternalistic to a participatory, patient-involving model, with shared decision-making as a key element. Schifferli hypothesises that disagreements between patients and physicians may be a consequence of increasing patient involvement and highlights the importance of patients' wish for participation in clinical decision-making.[24] However, not all patients may prefer shared decision- making and a personalized approach in this regard may be needed [25]. We found that disagreements between physicians and patients were a significant risk factor for low satisfaction in patients with sensitive topics. The example of our qualitative analysis in **Box 2** shows a potential for disagreement when patients wish to actively participate in the definition of treatment options, and the concept of best care differs between the patient and physician.

*Chief physician*: We know that you normally take Diazepam, which has a long half-life …

*Patient:* Yes, 24 hours.

*Chief physician*: Even longer, especially if the liver is not working properly. I've seen two people die from Diazepam. We prefer to prescribe Lorazepam

*Patient:* I do not tolerate Lorazepam.

*Chief physician*: What do you mean by "don't tolerate"?

*Patient:* Once when I withdrew from alcohol and doctors gave me Lorazepam I hallucinated. I saw a mouse in my room. Then I had delirium and woke up in another hospital.

*Chief physician*: We cannot assume that this was because of the Lorazepam. It was more likely due to alcohol withdrawal.

*Patient:* Yes, but I've never experienced anything like that with Diazepam.

*Chief physician*: It is important to me that we informed you about this …

***Box 2:** Example of a disagreement between patient and physician*

A recent study showed that patients with active decisional preference are less satisfied with their care and have less trust in the healthcare team compared to patients preferring less in depth involvement in medical decisions.[25] This is in line with the present findings, suggesting that disagreements are a risk factor for low satisfaction. A patient's wish for participation is therefore relevant, and a more personalised approach may improve the patient-physician relationship and increase



For peer review only - http://bmjopen.bmj.com/site/about/guidelines.xhtml

patients' satisfaction with medical care. However, as shown in our study, correctly addressing sensitive topics remains a major challenge during ward rounds due to several factors including lack of privacy, time constraints, lack of training and standards how to best address sensitive topcis  among others. Future research and quality initiatives should focus on this important issue to improve the care of patients with sensitive topics.

This trial has several limitations. First, we only included Swiss teaching hospitals. This might cause a selection bias and limits generalisability of the findings. Second, using a pragmatic approach, ward rounds in the three participating hospitals were not standardised regarding clinical practice and to ensure external validity. Consequently, the internal validity might be reduced. Third, the questionnaire used to assess patient perception has not yet been validated. Finally, there is no well accepted definition of sensitive topics and we thus defined it based on the clinical experience of the physician-researcher team. Still, there is some data suggesting that patients find value and comfort when their doctors openly discuss uncertainty with them.[26]  Yet, a sensitivity analysis excluding uncertainty from the definition of sensitive topics showed similar results.

## Conclusion

This analysis suggests that a large proportion of a broad medical inpatient sample have sensitive health topics, which was associated with lower satisfaction with care - particularly if the patient perceived patient-physician interactions during ward rounds as unsatisfactory. Specific physicians training in communication techniques how to identify, discuss and address sensitive topics may help to improve the care of these patients. Besides improving communication skills, future studies need to address the



For peer review only - http://bmjopen.bmj.com/site/about/guidelines.xhtml

question which sensitive topics can be discussed at the bedside or in a more private setting.

## Contributorship statement

All listed authors were involved in conducting the present study.

SG designed, analysed, and interpreted this ancillary analysis, and drafted the article.

CB designed the original trial, wrote the proposal for the ethics committee, provided study material or patients and critically revised the manuscript for important intellectual content.

KB collected patient data and critically revised the manuscript for important intellectual content.

VM analysed and interpreted patient data and critically revised the manuscript for important intellectual content.

JG contributed to design of this ancillary analyses and critically revised the manuscript for important intellectual content.

PS designed the original trial, contributed statistical expertise, and critically revised the manuscript for important intellectual content.

JL provided study material or patients and critically revised the manuscript for important intellectual content.

RS contributed to the study design and critically revised the manuscript for important intellectual content.



For peer review only - http://bmjopen.bmj.com/site/about/guidelines.xhtml

WL designed the original trial and critically revised the manuscript for important intellectual content.

MT provided study material or patients and critically revised the manuscript for important intellectual content.

TB provided study material or patients and critically revised the manuscript for important intellectual content.

JE provided study material or patients and critically revised the manuscript for important intellectual content.

MO provided study material or patients and critically revised the manuscript for important intellectual content.

SB designed the original trial, provided study material or patients, and critically revised the manuscript for important intellectual content.

SH designed the original trial, wrote the proposal for the ethics committee, analysed and interpreted patient data, drafted the article, critically revised the manuscript for important intellectual content, provided study material or patients, contributed statistical expertise, and obtained funding.

All authors approved the final manuscript.

## Competing interests

S. Hunziker and her research team are supported by the Swiss National Science Foundation (SNSF) (Ref 10001C_192850/1 and 10531C_182422) and the Gottfried Julia Bangerter-Rhyner Foundation (8472/HEG-DSV).



For peer review only - http://bmjopen.bmj.com/site/about/guidelines.xhtml

P. Schuetz has received support from the SNF (SNSF Professorship, PP00P3_150531), the Research Committee (Forschungsrat) of the Kantonsspital Aarau (1410.000.058 and 1410.000.044) and Funds of the Argovia Professorship of the Medical University Clinic (FG 1500000083). P. Schuetz has previously received unrestricted grant money unrelated to this project from Nestlé Health Science and Abbott Nutrition.

JD Leuppi and his research team are supported by the Swiss Personalized Health Network (Ref Driver-Project – 2018DR108)) and the Swiss National Science Foundation (SNSF) (Ref SNFS-160072 und -185592). JD Leuppi has also received unrestricted grant money unrelated to the project from AstraZeneca AG Switzerland, Boehringer GmbH Switzerland, GSK AG Switzerland, and Novartis AG Switzerland.

RS received funding from the Stanley Thomas Johnson Stiftung & Gottfried und Julia Bangerter-Rhyner-Stiftung under projects no. PC 28/17 and PC 05/18, from the Swiss Cancer League (Krebsliga Schweiz) under project no. KLS-4304-08-2017, from Promotion Santé Suisse *(Gesundheitsförderung Schweiz)* under contract no. 18.191/ K50001, and in the context of a Horizon Europe project from the Swiss State Secretariat for Education, Research and Innovation (SERI) under contract number 22.00094.RS received a speaker honorarium from Novartis.

MT is recipient of a project grant of the Swiss National Science Foundation (grant No. 320030_200423) and has research collaborations with Roche, Novartis and Idorsia (all Switzerland).

## Funding

Theoriginal trial was funded by the Swiss National Science Foundation grant number SNSF_182422.



For peer review only - http://bmjopen.bmj.com/site/about/guidelines.xhtml

## Data Sharing Statement

The data will be made available to parties placing a reasonable request to the lead author.

## Ethics statement

The BEDSIDE-OUTSIDE trial including this pre-planned ancillary analysis was registered prior to initiation at clinicaltrials.gov on 7 July 2017 (https://clinicaltrials.gov/ct2/show/NCT03210987) and approved by the local Ethics Committee (Northwest and Central Switzerland, EKNZ, 2017-00991).

## References


1. Cowles, K.V., *Issues in qualitative research on sensitive topics.* West J Nurs Res, 1988. **10**(2): p. 163-79.
2. Bressan, V., et al., *Patients experiences of bedside handover: findings from a meta-synthesis.* Scandinavian journal of caring sciences, 2019. **33**(3): p. 556-568.
3. Satterfield, J.M., et al., *The prevalence of social and behavioral topics and related educational opportunities during attending rounds.* Academic medicine: journal of the Association of American Medical Colleges, 2014. **89**(11): p. 1548.
4. Weber, H., et al., *Communication during ward rounds in internal medicine. An analysis of patient-nurse-physician interactions using RIAS.* Patient Educ Couns, 2007. **67**(3): p. 343-8.
5. Ahluwalia, S.C., et al., *Missed opportunities for advance care planning communication during outpatient clinic visits.* Journal of General Internal Medicine, 2012. **27**(4): p. 445-451.
6. Becker, C., et al., *Effect of Bedside Compared With Outside the Room Patient Case Presentation on Patients' Knowledge About Their Medical Care : A Randomized, Controlled, Multicenter Trial.* Ann Intern Med, 2021. **174**(9): p. 1282-1292.
7. Gross, S., et al., *Perception of physicians and nursing staff members regarding outside versus bedside ward rounds: ancillary analysis of the randomised BEDSIDE-OUTSIDE trial.* Swiss Medical Weekly, 2022. **152**.
8. Brahmania, M., et al., *Resident trainees do not affect patient satisfaction in an outpatient gastroenterology clinic: A prospective study conducted in a Canadian gastroenterology clinic.* Can J Gastroenterol Hepatol. **29**(7): p. 363-368.
9. Brown, J.D. and L.S. Wissow, *Discussion of sensitive health topics with youth during primary care visits: relationship to youth perceptions of care.* J Adolesc Health, 2009. **44**(1): p. 48-54.
10. Schulz, K.F., et al., *CONSORT 2010 Statement: updated guidelines for reporting parallel group randomised trials.* Trials, 2010. **11**: p. 32.


For peer review only



For peer review only - http://bmjopen.bmj.com/site/about/guidelines.xhtml

11. Kerim-Sade, C., A. Crispin, and J. Wasem, *An external control of validity of the German EuroQol-5D questionnaire.* Wirtschaftswissenschaftliche Diskussionspapiere, 2000. **No. 06/2000**.

12. Lesley F. Degner, J.A.S., Peri Venkatesh, *The Control Preference Scale.* Canadian Journal of Nursing Research, 1997. **29**(3): p. 21-43.

13. Degner, L., et al., *Information Needs and Decisional Preferences in Women With Breast Cancer.* JAMA, 1997. **277**(18): p. 1485-1492.

14. von Campenhausen, J., *Ärztliche Kommunikation für Medizinstudierende.* 2020: Springer Berlin Heidelberg.

15. Pollak, K.I., et al., *Oncologist communication about emotion during visits with patients with advanced cancer.* Journal of Clinical Oncology, 2007. **25**(36): p. 5748-5752.

16. Zimmermann, C., L. Del Piccolo, and A. Finset, *Cues and concerns by patients in medical consultations: a literature review.* Psychol Bull, 2007. **133**(3): p. 438-63.

17. Yoon, H., et al., *Factors Affecting Quality of Life for Korean American Cancer Survivors: An Integrative Review.* Oncol Nurs Forum, 2016. **43**(3): p. E132-42.

18. Lish, J.D., et al., *PSYCHIATRIC SCREENING IN PRIMARY CARE: WHAT DO PATIENTS REALLY WANT?* Journal of Psychosomatic Research, 1997. **43**: p. 167-175.

19. Alam, R., et al., *Managing diagnostic uncertainty in primary care: a systematic critical review.* BMC Fam Pract, 2017. **18**(1): p. 79.

20. Guan, T., et al., *Illness uncertainty, coping, and quality of life among patients with prostate cancer.* Psycho-Oncology, 2020. **29**(6): p. 1019-1025.

21. Bovier, P.A. and T.V. Perneger, *Stress from uncertainty from graduation to retirement - A population-based study of Swiss physicians.* Journal of General Internal Medicine, 2007. **22**(5): p. 632-638.

22. Papanagnou, D., et al., *Towards a medical school curriculum for uncertainty in clinical practice.* Med Educ Online, 2021. **26**(1): p. 1972762.

23. Santelli, J.S., et al., *Discussion of Potentially Sensitive Topics With Young People.* Pediatrics, 2019. **143**(2).

24. Schifferli, J.A., *[Shared decision making: a new challenge for the physicians?].* Praxis (Bern 1994), 2005. **94**(39): p. 1505-7.

25. Becker, C., et al., *Patients' Preference for Participation in Medical Decision-Making: Secondary Analysis of the BEDSIDE-OUTSIDE Trial.* J Gen Intern Med, 2022.

26. van Enk, A., et al., *Presenting cases in front of patients: implications for a key medical education genre.* Advances in Health Sciences Education, 2022. **27**(3): p. 621-643.

# Figure legends

### Figure 1. Sensitive topics and their frequency (n=791)



For peer review only - http://bmjopen.bmj.com/site/about/guidelines.xhtml

For peer review only - http://bmjopen.bmj.com/site/about/guidelines.xhtml

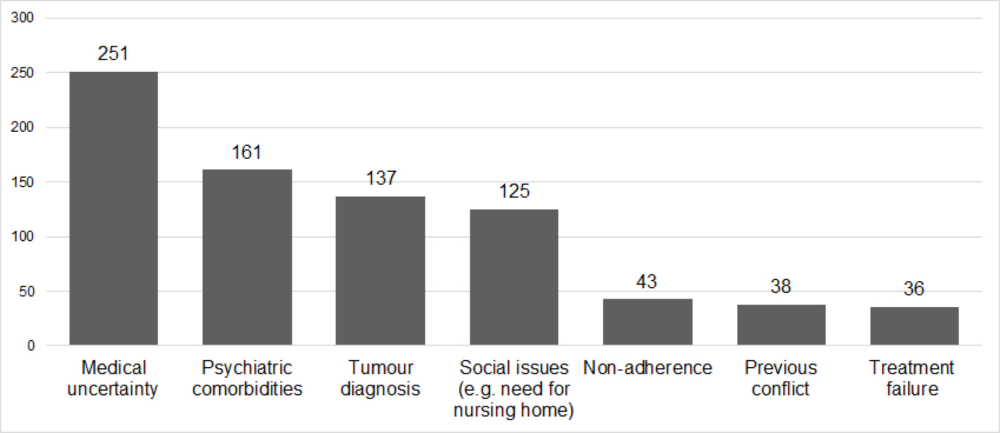


Figure 1. Sensitive topics and their frequency (n=791)

299x129mm (72 x 72 DPI)

# Supplementary Appendix

For peer review only

For peer review only - http://bmjopen.bmj.com/site/about/guidelines.xhtml

## Supplement 1. Associations of patient characteristics with sensitive topics

| | n | All | No Sensitive topics | Sensitive topics | *p-value* | univariable OR (95%CI) | *p-value* | OR (95%CI) *adjusted for centrum, intervention* | *p-value* |
|---|---|---|---|---|---|---|---|---|---|
| **n** | | | 445 | 474 | | | | | |
| **Sociodemographic factors** | | | | | | | | | |
| *Age, years; mean (SD)* | 919 | 65.0 (15.9) | 65.7 (15.9) | 64.3 (15.9) | *0.16* | 0.99 (0.99, 1) | *0.164* | 1 (0.99, 1) | *0.304* |
| *Female sex, n (%)* | 919 | 361 (39.3%) | 173 (38.9%) | 188 (39.7%) | *0.81* | 1.03 (0.79, 1.35) | *0.807* | 1.05 (0.8, 1.37) | *0.741* |
| *Number of children, mean (SD)* | 919 | 2.5 (9.2) | 2.1 (6.7) | 2.8 (11.0) | *0.26* | 1.01 (0.99, 1.02) | *0.275* | 1.01 (0.99, 1.02) | *0.365* |
| **Family status** | 919 | | | | | | | | |
| Single, relationship, married, registered partnership, n (%) | | 648 (70.5%) | 317 (71.2%) | 331 (69.8%) | *0.84* | 1 (ref.) | | | |
| Separated, divorced, n (%) | | 147 (16.0%) | 68 (15.3%) | 79 (16.7%) | | 1.11 (0.78, 1.59) | *0.56* | 1.05 (0.73, 1.52) | *0.787* |
| Widowed, n (%) | | 124 (13.5%) | 60 (13.5%) | 64 (13.5%) | | 1.02 (0.7, 1.5) | *0.913* | 1.07 (0.72, 1.59) | *0.738* |
| **Citizenship** | 919 | | | | | | | | |
| Switzerland, n (%) | | 789 (85.9%) | 386 (86.7%) | 403 (85.0%) | *0.71* | 1 (ref.) | | | |
| Germany, n (%) | | 55 (6.0%) | 26 (5.8%) | 29 (6.1%) | | 1.07 (0.62, 1.85) | *0.813* | 0.97 (0.55, 1.68) | *0.904* |
| Other, n (%) | | 75 (8.2%) | 33 (7.4%) | 42 (8.9%) | | 1.22 (0.76, 1.96) | *0.416* | 1.22 (0.75, 1.98) | *0.421* |
| **Highest level of education** | | | | | | | | | |
| No School graduation, n (%) | | 23 (2.5%) | 10 (2.2%) | 13 (2.7%) | *0.86* | 1 (ref.) | | | |
| School graduation, n (%) | | 141 (15.3%) | 67 (15.1%) | 74 (15.6%) | | 0.85 (0.35, 2.07) | *0.719* | 0.96 (0.39, 2.35) | *0.929* |
| Apprenticeship/Diploma, n (%) | | 631 (68.7%) | 309 (69.4%) | 322 (67.9%) | | 0.8 (0.35, 1.86) | *0.605* | 0.92 (0.39, 2.15) | *0.846* |
| University/Higher Education, n (%) | | 118 (12.8%) | 55 (12.4%) | 63 (13.3%) | | 0.88 (0.36, 2.17) | *0.783* | 0.89 (0.36, 2.21) | *0.799* |
| Missings, n (%) | | 6 (0.7%) | 4 (0.9%) | 2 (0.4%) | | 0.38 (0.06, 2.54) | *0.321* | 0.39 (0.06, 2.62) | *0.336* |
| **Occupation** | 919 | | | | | | | | |
| Employed / working + IV, n (%) | | 259 (28.2%) | 129 (29.0%) | 130 (27.4%) | ***0.041*** | 1 (ref.) | | | |
| Unemployed / homemaker, n (%) | | 39 (4.2%) | 13 (2.9%) | 26 (5.5%) | | 1.98 (0.98, 4.03) | *0.058* | 2 (0.97, 4.09) | *0.059* |
| Retired / IV support, n (%) | | 605 (65.8%) | 296 (66.5%) | 309 (65.2%) | | 1.04 (0.77, 1.39) | *0.812* | 1.05 (0.78, 1.42) | *0.735* |
| In education, n (%) | | 6 (0.7%) | 5 (1.1%) | 1 (0.2%) | | 0.2 (0.02, 1.72) | *0.142* | 0.19 (0.02, 1.63) | *0.128* |
| Other, n (%) | | 10 (1.1%) | 2 (0.4%) | 8 (1.7%) | | 3.97 (0.83, 19.05) | *0.085* | 3.41 (0.7, 16.56) | *0.128* |
| **Health-related factors** | 919 | | | | | | | | |
| ***Main admission diagnosis*** | | | | | | | | | |
| Coronary heart disease, n (%) | | 93 (10.1%) | 53 (11.9%) | 40 (8.4%) | ***<0.001*** | 1 (ref.) | | | |
| Congestive heart failure, n (%) | | 80 (8.7%) | 38 (8.5%) | 42 (8.9%) | | 1.46 (0.8, 2.67) | *0.213* | 1.7 (0.92, 3.15) | *0.09* |
| Cardiovascular diseases, n (%) | | 98 (10.7%) | 56 (12.6%) | 42 (8.9%) | | 0.99 (0.56, 1.76) | *0.983* | 1.09 (0.61, 1.95) | *0.78* |
| Infections, n (%) | | 207 (22.5%) | 116 (26.1%) | 91 (19.2%) | | 1.04 (0.63, 1.7) | *0.878* | 1.2 (0.73, 1.99) | *0.468* |
| Gastrointestinal diseases, n (%) | | 58 (6.3%) | 31 (7.0%) | 27 (5.7%) | | 1.15 (0.6, 2.23) | *0.67* | 1.15 (0.59, 2.25) | *0.687* |
| Metabolisms, n (%) | | 51 (5.5%) | 30 (6.7%) | 21 (4.4%) | | 0.93 (0.46, 1.85) | *0.831* | 1 (0.49, 2.02) | *0.999* |
| Tumour, n (%) | | 81 (8.8%) | 36 (8.1%) | 45 (9.5%) | | 1.66 (0.91, 3.02) | *0.1* | 1.64 (0.89, 3.04) | *0.115* |
| Other, n (%) | | 251 (27.3%) | 85 (19.1%) | 166 (35.0%) | | 2.59 (1.59, 4.21) | ***<0.001*** | 3.08 (1.87, 5.07) | ***<0.001*** |
| ***Comorbidities*** | 919 | | | | | | | | |
| Charlson Comorbidity Index, mean (SD) | | 4.45 (2.88) | 4.4 (2.8) | 4.5 (3.0) | *0.54* | 1.01 (0.97, 1.06) | *0.543* | 0.76 (0.59, 1) | ***0.048*** |
| Cardiology, n (%) | | 485 (52.8%) | 248 (55.7%) | 237 (50.0%) | *0.082* | 1.02 (0.98, 1.07) | *0.373* | 0.86 (0.65, 1.13) | *0.273* |
| Neurology, n (%) | | 193 (21.0%) | 80 (18.0%) | 113 (23.8%) | ***0.029*** | 0.79 (0.61, 1.03) | *0.082* | 1.01 (0.77, 1.33) | *0.941* |
| Rheumatology / Immunology, n (%) | | 143 (15.6%) | 68 (15.3%) | 75 (15.8%) | *0.82* | 0.82 (0.63, 1.07) | *0.15* | 1.03 (0.78, 1.37) | *0.819* |
| Gastrointestinal, n (%) | | 254 (27.6%) | 118 (26.5%) | 136 (28.7%) | *0.46* | 1.43 (1.04, 1.97) | ***0.03*** | 1.18 (0.85, 1.65) | *0.325* |
| Endocrinology, n (%) | | 352 (38.3%) | 185 (41.6%) | 167 (35.2%) | ***0.048*** | 1.46 (1.06, 2.03) | *0.022* | 1.21 (0.86, 1.71) | *0.264* |
| Respiratory, n (%) | | 315 (34.3%) | 152 (34.2%) | 163 (34.4%) | *0.94* | 1.04 (0.73, 1.49) | *0.821* | 1.07 (0.81, 1.4) | *0.627* |
| Infectious diseases, n (%) | | 171 (18.6%) | 77 (17.3%) | 94 (19.8%) | *0.33* | 1.11 (0.77, 1.62) | *0.57* | 1.12 (0.85, 1.48) | *0.416* |

For peer review only

| | | | | | | | | | |
|---|---|---|---|---|---|---|---|---|---|
| Renal, n (%) | | 317 (34.5%) | 150 (33.7%) | 167 (35.2%) | 0.63 | 1.12 (0.83, 1.49) | 0.461 | 0.25 (0.07, 0.91) | 0.035 |
| Gynaecology, n (%) | | 14 (1.5%) | 11 (2.5%) | 3 (0.6%) | 0.023 | 1.18 (0.87, 1.58) | 0.284 | 0.26 (0.07, 0.94) | 0.04 |
| Urology, n (%) | | 93 (10.1%) | 43 (9.7%) | 50 (10.5%) | 0.66 | 1.1 (0.72, 1.69) | 0.657 | 1.24 (0.8, 1.93) | 0.338 |
| Oncology, n (%) | | 287 (31.2%) | 145 (32.6%) | 142 (30.0%) | 0.39 | 0.88 (0.67, 1.17) | 0.391 | 1.81 (1.13, 2.9) | 0.014 |
| Psychiatry, n (%) | | 104 (11.3%) | 31 (7.0%) | 73 (15.4%) | <0.001 | 2.43 (1.56, 3.78) | <0.001 | 1.97 (1.21, 3.2) | 0.006 |
| Depression, n (%) | | 82 (8.9%) | 29 (6.5%) | 53 (11.2%) | 0.013 | 2.43 (1.56, 3.78) | <0.001 | 0.89 (0.68, 1.15) | 0.364 |
| Other, n (%) | | 498 (54.2%) | 248 (55.7%) | 250 (52.7%) | 0.36 | 2.54 (1.62, 3.99) | <0.001 | 0.95 (0.73, 1.25) | 0.734 |
| **Health self rating VAS [0-100], mean (SD)** | 919 | 56.9 (22.32) | 58.64 (21.97) | 55.25 (22.55) | 0.021 | 0.99 (0.99, 1) | 0.022 | 0.99 (0.99, 1) | 0.018 |
| **Quality of life (EQ-5D) Index, mean (SD)** | 919 | 0.71 (0.3) | 0.75 (0.27) | 0.67 (0.3) | <0.001 | 0.38 (0.24, 0.6) | <0.001 | 0.4 (0.25, 0.65) | <0.001 |
| **Patient's Decisional Control Preferences** | 919 | | | | | | | | |
| SDM, n (%) | | 473 (62.2%) | 234 (61.1%) | 239 (63.2%) | 0.54 | 1 (ref.) | | | |
| active, n (%) | | 117 (15.4%) | 57 (14.9%) | 60 (15.9%) | 0.70 | 1.19 (0.84, 1.69) | 0.332 | 1.22 (0.86, 1.75) | 0.268 |
| passive, n (%) | | 171 (22.5%) | 92 (24.0%) | 79 (20.9%) | 0.30 | 1.23 (0.77, 1.96) | 0.397 | 1.24 (0.77, 2) | 0.382 |
| **Intervention of the original study, n %** | 919 | | | | | | | | |
| Bedside ward round | | 476 (51.8%) | 257 (57.8%) | 219 (46.2%) | <0.001 | 1 (ref.) | | | |
| Outside the rooum ward round | | 443 (48.2%) | 188 (42.2%) | 255 (53.8%) | | 0.63 (0.48, 0.82) | <0.001 | 0.64 (0.49, 0.83)* | 0.001 |

**adjusted for study centre, intervention. Odds ratios were calculated with logistic regression models. Abbreviations: OR, odds ratio; SD, standard deviation; CI, confidence interval; IV, Swiss disability insurance; n, number*

For peer review only

## Supplement 2. Associations of communication factors with sensitive topics

| | n | No Sensitive topics | Sensitive topics | *p-value* | OR (95%CI) | *p-value* | Adjusted OR (95%CI) *adjusted for centrum, intervention* | *p-value* |
|---|---|---|---|---|---|---|---|---|
| | | 445 | 474 | | | | | |
| **WEMS-Technique communication** | 919 | | | | | | | |
| Waiting, n (%) | | 71 (16.0%) | 55 (11.6%) | *0.055* | 0.69 (0.47, 1.01) | *0.056* | 0.83 (0.56, 1.24) | *0.37* |
| Echoing, n (%) | | 108 (24.3%) | 107 (22.6%) | *0.54* | 0.91 (0.67, 1.23) | *0.544* | 1.02 (0.74, 1.41) | *0.902* |
| Mirroring, n (%) | | 52 (11.7%) | 40 (8.4%) | *0.10* | 0.7 (0.45, 1.08) | *0.103* | 0.82 (0.52, 1.28) | *0.377* |
| Summarising, n (%) | | 44 (9.9%) | 39 (8.2%) | *0.38* | 0.82 (0.52, 1.28) | *0.381* | 0.84 (0.53, 1.33) | *0.464* |
| Open-ended questions, n (%) | | 352 (79.1%) | 348 (73.4%) | ***0.043*** | 0.73 (0.54, 0.99) | ***0.044*** | 0.77 (0.56, 1.05) | *0.102* |
| Confirming, n (%) | | 211 (47.4%) | 195 (41.1%) | *0.056* | 0.78 (0.6, 1.01) | *0.056* | 0.79 (0.61, 1.03) | *0.084* |
| Nodding, n (%) | | 344 (77.3%) | 362 (76.4%) | *0.74* | 0.95 (0.7, 1.29) | *0.738* | 1.09 (0.79, 1.49) | *0.611* |
| **NURSE-Technique communication in patients with emotions (n = 115)** | 115 | 34 | 81 | | | | | |
| Visible evidence of emotion, n (%) | | 7 (21%) | 16 (20%) | *0.92* | 0.95 (0.35, 2.57) | *0.919* | 0.95 (0.33, 2.78) | *0.929* |
| Understanding (verbal/nonverbal), n (%) | | 8 (24%) | 23 (28%) | *0.59* | 1.29 (0.51, 3.26) | *0.592* | 1.24 (0.46, 3.35) | *0.667* |
| Respecting, n (%) | | 4 (12%) | 26 (32%) | ***0.023*** | 3.55 (1.13, 11.12) | ***0.03*** | 2.7 (0.79, 9.25) | *0.114* |
| Supporting, n (%) | | 9 (26%) | 25 (31%) | *0.64* | 1.24 (0.51, 3.04) | *0.638* | 1.24 (0.48, 3.23) | *0.66* |
| Exploring, n (%) | | 9 (26%) | 25 (31%) | *0.64* | 1.24 (0.51, 3.04) | *0.638* | 1.24 (0.48, 3.23) | *0.66* |
| Hasty solace, n (%) | | 0 (0%) | 3 (4%) | *0.26* | n.a. | n.a. | n.a. | n.a. |
| Distracting/Ignoring, n (%) | | 0 (0%) | 3 (4%) | *0.26* | n.a. | n.a. | n.a. | n.a. |
| Dismissing ("that's not so bad"), n (%) | | 4 (12%) | 4 (5%) | *0.19* | 0.39 (0.09, 1.66) | *0.202* | 0.58 (0.12, 2.84) | *0.505* |
| Replies with providing information instead of addressing emotion, n (%) | | 15 (44%) | 37 (46%) | *0.88* | 1.07 (0.48, 2.38) | *0.878* | 1.13 (0.48, 2.66) | *0.785* |
| Physician talks about emotion, n (%) | | 4 (12%) | 9 (11%) | *0.92* | 0.94 (0.27, 3.28) | *0.92* | 0.79 (0.21, 3.03) | *0.734* |
| Overall rating: Responding to emotions, n (%) | | 14 (44%) | 28 (35%) | *0.39* | 0.69 (0.3, 1.6) | *0.389* | 0.64 (0.26, 1.61) | *0.346* |
| **Other communication factors** | 919 | | | | | | | |
| Occurrence of emotion, n (%) | | 48 (10.8%) | 70 (14.8%) | *0.071* | 1.43 (0.97, 2.12) | *0.072* | 1.35 (0.9, 2.01) | *0.144* |
| Medical staff talks to patient about diagnosis, n (%) | | 215 (48.3%) | 271 (57.2%) | ***0.007*** | 1.43 (1.1, 0) | ***0.007*** | 1.59 (1.21, 2.09) | ***0.001*** |
| Medical staff talks to patient about symptoms, n (%) | | 399 (89.7%) | 443 (93.5%) | ***0.038*** | 1.65 (1.02, 2.65) | ***0.039*** | 1.66 (1.02, 2.69) | ***0.041*** |
| Medical staff talks to patient about treatment, n (%) | | 334 (75.1%) | 383 (80.8%) | ***0.036*** | 1.4 (1.02, 1.91) | ***0.036*** | 1.38 (1, 1.9) | ***0.049*** |
| Medical staff talks to patient about next steps, n (%) | | 344 (77.3%) | 431 (90.9%) | ***<0.001*** | 2.94 (2, 4.32) | ***<0.001*** | 2.8 (1.89, 4.13) | ***<0.001*** |
| Medical staff talks to patient about social issues, n (%) | | 272 (61.1%) | 320 (67.5%) | ***0.043*** | 1.32 (1.01, 1.73) | *0.043* | 1.43 (1.08, 1.9) | ***0.013*** |
| Medical staff talks about patient instead of with patient after case discussion, n (%) | | 329 (73.9%) | 351 (74.1%) | *0.97* | 1.01 (0.75, 1.35) | *0.967* | 1.19 (0.87, 1.62) | *0.282* |
| Physician and patient talk at cross purposes, n (%) | | 17 (3.8%) | 35 (7.4%) | ***0.019*** | 2.01 (1.11, 3.64) | ***0.022*** | 2.29 (1.24, 4.25) | ***0.008*** |
| Patient seems not to understand something, but does not ask, n (%) | | 6 (1.3%) | 17 (3.6%) | ***0.030*** | 2.72 (1.06, 6.97) | ***0.037*** | 4.15 (1.55, 11.08) | ***0.005*** |
| Physician disagrees with / corrects patient, n (%) | | 30 (6.7%) | 57 (12.0%) | ***0.006*** | 1.89 (1.19, 3) | ***0.007*** | 2.05 (1.28, 3.3) | ***0.003*** |
| Patient disagrees with / corrects physician, n (%) | | 27 (6.1%) | 53 (11.2%) | ***0.006*** | 1.95 (1.2, 3.16) | ***0.007*** | 2.12 (1.29, 3.48) | ***0.003*** |
| Occurrence of *"cue"*, n (%) | | 32 (7.2%) | 79 (16.7%) | ***<0.001*** | 2.58 (1.67, 3.98) | ***<0.001*** | 2.36 (1.52, 3.66) | ***<0.001*** |
| Addressing *"cue"* concern, n (%) | | 19 (4.3%) | 40 (8.4%) | ***0.010*** | 2.07 (1.18, 3.63) | ***0.011*** | 1.83 (1.03, 3.24) | ***0.039*** |
| Reacts by providing information instead of exploration, n (%) | | 19 (4.3%) | 33 (7.0%) | *0.077* | 1.68 (0.94, 3) | *0.08* | 1.59 (0.88, 2.87) | *0.122* |

**adjusted for study centre, intervention. Odds ratios were calculated with logistic regression models. Abbreviations: OR, odds ratio; CI, confidence interval; cue, current unvoiced element; n, number*

For peer review only

## Supplement 3. Association of sensitive topics with various outcomes

| | n | No Sensitive topics | Sensitive topics | *p-value* | Difference or OR (95%CI) | *p-value* | Adjusted difference or OR (95%CI) *adjusted for centrum, intervention* | *p-value* |
|---|---|---|---|---|---|---|---|---|
| **Primary endpoint** | | 445 | 474 | | | | | |
| Overall satisfaction (VAS 0-100), mean (SD) | 906 | 90.2 (12.1) | 87.7 (14.6) | ***0.006*** | -2.49 (-4.24, -0.73) | ***0.006*** | -2.5 (-4.28, -0.72) | ***0.006*** |
| **Secondary endpoints** | | | | | | | | |
| **Satisfaction with care (VAS 0-100)** | | | | | | | | |
| Overall satisfaction with ward round, mean (SD) | 898 | 89.9 (16.1) | 86.6 (19.9) | ***0.006*** | -3.33 (-5.71, -0.95) | ***0.006*** | -3.51 (-5.93, -1.09) | ***0.005*** |
| Overall satisfaction with hospital stay, mean (SD) | 901 | 88.3 (17.0) | 86.8 (16.7) | *0.18* | -1.52 (-3.72, 0.68) | *0.176* | -1.1 (-3.32, 1.11) | *0.328* |
| Overall satisfaction with medical care, mean (SD) | 891 | 91.9 (14.1) | 90.0 (16.0) | *0.069* | -1.84 (-3.83, 0.14) | *0.069* | -1.97 (-3.99, 0.06) | *0.057* |
| Overall satisfaction with communication with physicians during ward round, mean (SD) | 886 | 90.7 (15.9) | 86.2 (21.7) | ***<0.001*** | -4.48 (-7.01, -1.95) | ***0.001*** | -4.39 (-6.97, -1.82) | ***0.001*** |
| Overall satisfaction with communication with nursing team during ward round , mean (SD) | 622 | 92.3 (16.6) | 90.0 (17.7) | *0.099* | -2.28 (-4.99, 0.43) | *0.099* | -2.45 (-5.21, 0.31) | *0.082* |
| **Patient knowledge about medical care** | | | | | | | | |
| Average subjective knowledge about their medical care, mean (SD) | 919 | 81.2 (19.0) | 77.8 (22.0) | ***0.013*** | -3.38 (-6.05, -0.71) | ***0.013*** | -3.86 (-6.57, -1.15) | ***0.005*** |
| Subjective understanding of disease, mean (SD) | 902 | 82.7 (19.8) | 80.4 (21.8) | *0.092* | -2.34 (-5.07, 0.39) | *0.092* | -2.7 (-5.48, 0.08) | *0.057* |
| Subjective understanding of therapeutic approach, mean (SD) | 861 | 80.8 (23.3) | 76.0 (27.8) | ***0.006*** | -4.83 (-8.28, -1.38) | ***0.006*** | -5.77 (-9.25, -2.29) | ***0.001*** |
| Subjective understanding of further plans for care, mean (SD) | 851 | 79.8 (25.0) | 76.1 (27.8) | ***0.042*** | -3.71 (-7.27, -0.14) | ***0.042*** | -3.99 (-7.61, -0.37) | ***0.031*** |
| Average objective knowledge about their medical care, mean (SD) | 919 | 72.6 (24.9) | 68.7 (25.8) | ***0.021*** | -3.88 (-7.17, -0.6) | ***0.021*** | -3.83 (-7.18, -0.48) | *0.025* |
| Objective understanding of disease, mean (SD) | 916 | 71.5 (32.2) | 66.3 (33.4) | ***0.016*** | -5.22 (-9.48, -0.96) | ***0.016*** | -5.24 (-9.59, -0.9) | ***0.018*** |
| Objective understanding of therapeutic approach, mean (SD) | 908 | 71.3 (34.2) | 68.4 (35.0) | *0.21* | -2.9 (-7.42, 1.61) | *0.208* | -2.39 (-6.99, 2.22) | *0.309* |
| Objective understanding of further plans for care, mean (SD) | 916 | 75.3 (31.5) | 71.2 (34.0) | *0.061* | -4.08 (-8.34, 0.19) | *0.061* | -4.4 (-8.73, -0.06) | *0.047* |
| **Measured timeliness of ward round** | | | | | | | | |
| Duration of outside the room discussions (min), mean (SD) | 919 | 2.8 (3.6) | 4.9 (5.0) | ***<0.001*** | 2.17 (1.6, 2.73) | ***<0.001*** | 1.41 (1.02, 1.81) | ***<0.001*** |
| Duration of bedside discussions (min), mean (SD) | 919 | 8.2 (4.2) | 9.0 (5.0) | ***0.005*** | 0.88 (0.27, 1.48) | ***0.005*** | 1.56 (1.03, 2.09) | ***<0.001*** |
| Duration of debriefing outside the room (min), mean (SD) | 919 | 0.4 (1.1) | 0.5 (1.1) | *0.063* | 0.14 (-0.01, 0.29) | *0.063* | 0.16 (0.01, 0.31) | ***0.038*** |
| Total duration of ward round per patient (min), mean (SD) | 919 | 11.3 (4.6) | 14.5 (5.6) | ***<0.001*** | 3.18 (2.52, 3.85) | ***<0.001*** | 3.13 (2.47, 3.79) | ***<0.001*** |
| **Patient perception regarding time spent on ward round** | | | | | | | | |
| Overall duration of ward round was sufficient (VAS 0-100), mean (SD) | 859 | 91.2 (18.3) | 89.9 (21.9) | *0.35* | -1.3 (-4, 1.41) | *0.346* | -1.21 (-3.98, 1.55) | *0.389* |
| Time spent with physicians was sufficient (VAS 0-100), mean (SD) | 771 | 90.2 (18.0) | 89.3 (20.0) | *0.52* | -0.88 (-3.58, 1.81) | *0.52* | -0.89 (-3.64, 1.86) | *0.525* |
| Patients estimation on the time spent with patient on ward round (min), mean (SD) | 856 | 11.2 (6.0) | 11.7 (7.1) | *0.27* | 0.5 (-0.39, 1.38) | *0.271* | 0.94 (0.06, 1.81) | ***0.036*** |
| Patients estimation on the time spent per day with patient case overall (min), mean (SD) | 719 | 69.6 (78.0) | 73.2 (85.1) | *0.55* | 3.63 (-8.34, 15.6) | *0.552* | 5.26 (-6.9, 17.42) | *0.396* |
| The ward round was helpful for better | | | | | | | | |
| - understanding the main illness (VAS 0-100), mean (SD) | 773 | 69.5 (34.3) | 62.7 (37.3) | ***0.009*** | -6.75 (-11.82, -1.69) | ***0.009*** | -6.21 (-11.4, -1.01) | ***0.019*** |
| - further therapeutic measures (VAS 0-100), mean (SD) | 714 | 68.7 (34.6) | 60.5 (37.2) | ***0.002*** | -8.22 (-13.51, -2.92) | ***0.002*** | -7.77 (-13.19, -2.36) | ***0.005*** |
| - further plans of care (VAS 0-100), mean (SD) | 755 | 71.1 (33.9) | 69.9 (67.5) | *0.76* | -1.21 (-8.88, 6.47) | *0.758* | -1.49 (-9.33, 6.35) | *0.71* |
| All my questions were answered (VAS 0-100), mean (SD) | 752 | 93.0 (15.6) | 89.7 (21.0) | ***0.017*** | -3.25 (-5.91, -0.59) | ***0.017*** | -3.29 (-6.01, -0.57) | ***0.018*** |
| I was able to understand all answers to my questions (VAS 0-100), mean (SD) | 747 | 93.3 (15.3) | 91.6 (18.3) | *0.19* | -1.64 (-4.07, 0.8) | *0.187* | -1.94 (-4.43, 0.54) | *0.125* |
| Information during visit has been adequate (yes, %) | 907 | 358 (81.2%) | 370 (79.4%) | *0.50* | 0.89 (0.64, 1.24) | *0.501* | 0.89 (0.64, 1.25) | *0.501* |
| Patient did not understand something during the round (yes, %) | 908 | 54 (12.2%) | 49 (10.5%) | *0.41* | 0.84 (0.56, 1.27) | *0.406* | 0.91 (0.6, 1.39) | *0.679* |
| Estimation of my participation in the discussion (VAS 0-100), mean (SD) | 837 | 63.8 (38.0) | 59.3 (32.2) | *0.064* | -4.51 (-9.28, 0.27) | *0.064* | -4.67 (-9.5, 0.16) | *0.058* |
| Disease was explained in an understandable way (VAS 0-100), mean (SD) | 720 | 84.3 (27.0) | 84.7 (26.3) | *0.84* | 0.4 (-3.5, 4.3) | *0.841* | 0.16 (-3.81, 4.12) | *0.939* |
| Treatment was explained in an understandable way (VAS 0-100), mean (SD) | 707 | 82.1 (28.5) | 80.5 (30.2) | *0.48* | -1.56 (-5.9, 2.78) | *0.48* | -1.61 (-6.04, 2.81) | *0.474* |
| Upcoming examinations were explained in an understandable way (VAS 0-100), mean (SD) | 635 | 82.6 (27.9) | 83.5 (28.2) | *0.71* | 0.84 (-3.53, 5.22) | *0.705* | 0.96 (-3.51, 5.43) | *0.673* |
| The information given during the visit was clear and understandable (VAS 0-100), mean (SD) | 859 | 92.1 (15.9) | 90.3 (19.6) | *0.15* | -1.76 (-4.16, 0.63) | *0.149* | -1.78 (-4.23, 0.67) | *0.154* |
| **Patient perception regarding discomfort during the ward round** | | | | | | | | |
| Medical terms used during ward round were confusing (VAS 0-100), mean (SD) | 853 | 15.9 (28.2) | 19.2 (30.4) | *0.099* | 3.32 (-0.63, 7.27) | *0.099* | 3.89 (-0.07, 7.85) | *0.054* |
| Ward round discussions made me worry (VAS 0-100), mean (SD) | 871 | 6.6 (25.7) | 11.7 (24.8) | ***0.003*** | 5.13 (1.78, 8.49) | ***0.003*** | 4.82 (1.4, 8.24) | ***0.006*** |
| I felt uncomfortable during ward round (VAS 0-100), mean (SD) | 870 | 4.5 (15.6) | 8.2 (21.6) | ***0.005*** | 3.61 (1.1, 6.13) | ***0.005*** | 3.2 (0.64, 5.76) | ***0.014*** |
| Ward round discussions unsettled me (VAS 0-100), mean (SD) | 752 | 4.4 (15.9) | 7.2 (19.9) | ***0.033*** | 2.81 (0.23, 5.4) | ***0.033*** | 2.84 (0.22, 5.45) | ***0.034*** |
| **Patient perception regarding physician`s behaviour during the ward round** | | | | | | | | |

For peer review only

| | | | | | | | | |
|---|---|---|---|---|---|---|---|---|
| Physicians treated me with respect (VAS 0-100), mean (SD) | 867 | 97.4 (8.8) | 95.5 (13.1) | ***0.011*** | -1.94 (-3.43, -0.45) | ***0.011*** | -2.05 (-3.57, -0.53) | ***0.008*** |
| I was taken seriously (VAS 0-100), mean (SD) | 861 | 96.1 (10.9) | 94.0 (15.6) | ***0.021*** | -2.13 (-3.94, -0.32) | ***0.021*** | -2.17 (-4.01, -0.32) | ***0.021*** |
| Physicians respected my privacy (VAS 0-100), mean (SD) | 794 | 89.7 (24.8) | 91.1 (20.9) | *0.38* | 1.42 (-1.78, 4.61) | *0.384* | 1.41 (-1.85, 4.66) | *0.396* |
| Physicians showed compassion (VAS 0-100), mean (SD) | 693 | 81.0 (28.4) | 75.2 (33.5) | ***0.014*** | -5.86 (-10.53, -1.2) | ***0.014*** | -5.56 (-10.33, -0.8) | ***0.022*** |
| My issues were dealt with discreetly (VAS 0-100), mean (SD) | 764 | 88.3 (23.7) | 89.2 (21.1) | *0.55* | 0.97 (-2.21, 4.16) | *0.549* | 1.33 (-1.91, 4.57) | *0.42* |
| Some topics during ward round communication caused inconvenience (VAS 0-100), mean (SD) | 853 | 4.0 (15.4) | 7.6 (21.8) | ***0.005*** | 3.61 (1.07, 6.15) | ***0.005*** | 3.58 (0.99, 6.17) | ***0.007*** |
| I was encouraged to address personal topics (VAS 0-100), mean (SD) | 624 | 85.5 (30.5) | 84.4 (30.2) | *0.65* | -1.11 (-5.88, 3.66) | *0.648* | -1.41 (-6.28, 3.46) | *0.57* |
| My privacy was violated (VAS 0-100), mean (SD) | 831 | 4.2 (16.2) | 3.3 (13.3) | *0.38* | -0.9 (-2.91, 1.12) | *0.383* | -1.17 (-3.23, 0.88) | *0.264* |
| Teaching took place during ward round (yes, %) | 909 | 136 (30.8%) | 126 (26.9%) | *0.19* | 0.83 (0.62, 1.1) | *0.193* | 0.96 (0.71, 1.3) | *0.812* |
| - if yes , teaching was perceived as disruptive, (VAS 0-100) mean (SD) | 257 | 1.7 (8.4) | 5.7 (21.9) | *0.051* | 4.01 (-0.02, 8.05) | ***0.05*** | 4.14 (0.08, 8.2) | ***0.046*** |
| **Patients' perception regarding quality of care (VAS 0-100)** | | | | | | | | |
| I felt "in good hands" in this hospital, mean (SD) | 883 | 90.6 (15.1) | 89.4 (15.6) | *0.27* | -1.15 (-3.18, 0.88) | *0.266* | -0.68 (-2.75, 1.38) | *0.516* |
| I felt there were contradicting statements from physicians and nursing team , mean (SD) | 843 | 11.6 (24.8) | 13.5 (26.6) | *0.27* | 1.95 (-1.53, 5.43) | *0.272* | 1.55 (-2, 5.1) | *0.392* |
| I feel confident with the physician team, mean (SD) | 873 | 91.9 (14.4) | 89.5 (16.8) | ***0.025*** | -2.39 (-4.47, -0.31) | ***0.025*** | -2.52 (-4.65, -0.4) | ***0.02*** |
| I feel confident with the nursing team, mean (SD) | 876 | 93.2 (12.6) | 91.7 (13.7) | *0.093* | -1.49 (-3.24, 0.25) | *0.093* | -1.7 (-3.48, 0.08) | *0.061* |
| There is good collaboration of physicians and nurses, mean (SD) | 809 | 92.3 (12.4) | 89.6 (14.9) | ***0.007*** | -2.63 (-4.54, -0.73) | ***0.007*** | -2.95 (-4.89, -1.01) | ***0.003*** |
| I feel physicians have high competence to treat the current illness , mean (SD) | 834 | 93.3 (43.0) | 89.4 (16.9) | *0.081* | -3.93 (-8.34, 0.48) | *0.081* | -3.66 (-8.16, 0.84) | *0.111* |
| I feel nurses have high competence to treat the current illness, mean (SD) | 845 | 92.7 (11.5) | 91.2 (25.9) | *0.28* | -1.5 (-4.21, 1.21) | *0.279* | -1.62 (-4.38, 1.15) | *0.251* |

**adjusted for study centre, intervention. All differences calculated with linear regression models for continuous data. Odds ratios were calculated with logistic regression models. Abbreviations: OR, odds ratio; SD, standard deviation; CI, confidence interval; VAS, visual analogue scale; n, number*

For peer review only

## Supplement 4. Risk factors for low satisfaction among patients with sensitive topics

| | n | high Satisfaction | low Satisfaction | *p-value* | Difference or OR (95%CI) | *p-value* | Adjusted difference or OR (95%CI) *adjusted for centrum, intervention* | *p-value* |
|---|---|---|---|---|---|---|---|---|
| | | 212 | 254 | | | | | |
| **WEMS-Technique communication** | 466 | | | | | | | |
| Waiting, n (%) | | 24 (11.3%) | 30 (11.8%) | *0.87* | 1.05 (0.59, 1.86) | *0.869* | *1.09 (0.61, 1.96)* | *0.766* |
| Echoing, n (%) | | 43 (20.3%) | 64 (25.2%) | *0.21* | 1.32 (0.85, 2.05) | *0.21* | *1.47 (0.92, 2.33)* | *0.107* |
| Mirroring, n (%) | | 14 (6.6%) | 26 (10.2%) | *0.16* | 1.61 (0.82, 3.17) | *0.166* | *1.75 (0.87, 3.51)* | *0.115* |
| Summarising, n (%) | | 18 (8.5%) | 21 (8.3%) | *0.93* | 0.97 (0.5, 1.88) | *0.931* | *1.01 (0.52, 1.96)* | *0.982* |
| Open-ended questions, n (%) | | 158 (74.5%) | 188 (74.0%) | *0.90* | 0.97 (0.64, 1.48) | *0.9* | *1 (0.65, 1.52)* | *0.982* |
| Confirming, n (%) | | 90 (42.5%) | 102 (40.2%) | *0.62* | 0.91 (0.63, 1.32) | *0.616* | *0.92 (0.63, 1.33)* | *0.64* |
| Nodding, n (%) | | 160 (75.5%) | 197 (77.6%) | *0.60* | 1.12 (0.73, 1.73) | *0.596* | *1.16 (0.75, 1.79)* | *0.505* |
| **NURSE-Technique communication (in patients with emotions)** | | 34 | 46 | | | | | |
| Naming (of emotion), n (%) | 78 | 8 (24%) | 7 (15%) | *0.35* | 0.58 (0.19, 1.8) | *0.35* | *0.46 (0.14, 1.47)* | *0.189* |
| Understanding (verbal/nonverbal), n (%) | 78 | 8 (24%) | 15 (33%) | *0.38* | 1.57 (0.58, 4.29) | *0.377* | *1.59 (0.55, 4.61)* | *0.395* |
| Respecting, n (%) | 78 | 13 (38%) | 12 (26%) | *0.25* | 0.57 (0.22, 1.48) | *0.249* | *0.37 (0.13, 1.09)* | *0.07* |
| Supporting, n (%) | 78 | 13 (38%) | 12 (26%) | *0.25* | 0.57 (0.22, 1.48) | *0.249* | *0.54 (0.2, 1.48)* | *0.232* |
| Exploring, n (%) | 78 | 13 (38%) | 12 (26%) | *0.25* | 0.57 (0.22, 1.48) | *0.249* | *0.54 (0.2, 1.48)* | *0.232* |
| Hasty solcae, n (%) | 78 | 1 (3%) | 2 (4%) | *0.74* | 1.5 (0.13, 17.25) | *0.745* | *1.54 (0.13, 19.05)* | *0.735* |
| Distracting/Ignoring, n (%) | 78 | 1 (3%) | 2 (4%) | *0.74* | 1.5 (0.13, 17.25) | *0.745* | *1.54 (0.13, 19.05)* | *0.735* |
| Dismissing (“that’s not so bad”), n (%) | 78 | 2 (6%) | 2 (4%) | *0.76* | 0.73 (0.1, 5.44) | *0.756* | *0.61 (0.08, 4.69)* | *0.636* |
| Replies with providing information instead of addressing emotion, n (%) | 78 | 16 (47%) | 20 (43%) | *0.75* | 0.87 (0.36, 2.11) | *0.75* | *0.88 (0.35, 2.23)* | *0.793* |
| Physician talks about emotion, n (%) | 78 | 6 (18%) | 3 (7%) | *0.12* | 0.33 (0.08, 1.41) | *0.133* | *0.35 (0.07, 1.67)* | *0.188* |
| Overall rating: Responding to emotions, n (%) | 77 | 13 (38%) | 14 (31%) | *0.51* | 0.73 (0.29, 1.86) | *0.509* | *0.98 (0.36, 2.67)* | *0.969* |
| **Other communication factors** | 466 | | | | | | | |
| Medical staff talks to patient about diagnosis, n (%) | | 117 (55.2%) | 149 (58.7%) | *0.45* | 1.15 (0.8, 1.67) | *0.451* | *1.19 (0.82, 1.73)* | *0.368* |
| Medical staff talks to patient about symptoms, n (%) | | 198 (93.4%) | 237 (93.3%) | *0.97* | 0.99 (0.47, 2.05) | *0.969* | *1.02 (0.49, 2.12)* | *0.963* |
| Medical staff talks to patient about treatment, n (%) | | 167 (78.8%) | 209 (82.3%) | *0.34* | 1.25 (0.79, 1.98) | *0.34* | *1.23 (0.78, 1.96)* | *0.375* |
| Medical staff talks to patient about next steps, n (%) | | 190 (89.6%) | 234 (92.1%) | *0.35* | 1.35 (0.72, 2.56) | *0.349* | *1.33 (0.71, 2.52)* | *0.376* |

For peer review only

| | | | | | | | | |
|---|---|---|---|---|---|---|---|---|
| Medical staff talks to patient about social issues, n (%) | | 151 (71.2%) | 164 (64.6%) | 0.13 | 0.74 (0.5, 1.09) | 0.127 | 0.75 (0.5, 1.12) | 0.163 |
| Medical staff talks about instead of with the patient after case discussion, n (%) | | 157 (74.1%) | 189 (74.4%) | 0.93 | 1.02 (0.67, 1.55) | 0.931 | 1.07 (0.7, 1.65) | 0.748 |
| Physician and patient talk talk at cross purposes, n (%) | | 17 (8.0%) | 16 (6.3%) | 0.47 | 0.77 (0.38, 1.57) | 0.472 | 0.78 (0.38, 1.58) | 0.486 |
| Patient does not seem to understand something, but does not ask, n (%) | | 9 (4.2%) | 8 (3.1%) | 0.53 | 0.73 (0.28, 1.94) | 0.531 | 0.78 (0.29, 2.11) | 0.624 |
| Physician disagrees with/corrects patient, n (%) | | 14 (6.6%) | 41 (16.1%) | **0.001** | 2.72 (1.44, 5.15) | **0.002** | 2.78 (1.47, 5.27) | **0.002** |
| Patient disagrees with/corrects physician, n (%) | | 14 (6.6%) | 37 (14.6%) | **0.006** | 2.41 (1.27, 4.59) | **0.007** | 2.42 (1.27, 4.61) | **0.007** |
| Occurrence of cue, n (%) | | 28 (13.2%) | 49 (19.3%) | 0.078 | 1.57 (0.95, 2.6) | 0.08 | 1.55 (0.93, 2.57) | 0.093 |
| Addressing cue concern, n (%) | | 17 (8.0%) | 22 (8.7%) | 0.80 | 1.09 (0.56, 2.11) | 0.803 | 1.05 (0.54, 2.04) | 0.888 |
| Replies with providing information instead of exploration, n (%) | | 10 (4.7%) | 22 (8.7%) | 0.094 | 1.92 (0.89, 4.14) | 0.098 | 1.91 (0.88, 4.14) | 0.1 |
| **Patient knowledge about medical care** | 466 | | | | | | | |
| Average subjective knowledge about their medical care, mean (SD) | | 87.0 (16.2) | 70.7 (22.9) | **<0.001** | 0.96 (0.94, 0.97) | **<0.001** | 0.95 (0.94, 0.97) | **<0.001** |
| Subjective understanding of disease, mean (SD) | | 88.48 (16.27) | 74.14 (23.03) | **<0.001** | 0.96 (0.95, 0.97) | **<0.001** | 0.96 (0.95, 0.97) | **<0.001** |
| Subjective understanding of therapeutic approach, mean (SD) | | 85.18 (23.36) | 69.55 (27.99) | **<0.001** | 0.98 (0.97, 0.98) | **<0.001** | 0.97 (0.96, 0.98) | **<0.001** |
| Subjective understanding of further plans for care, mean (SD) | | 87.14 (22.39) | 68.21 (27.81) | **<0.001** | 0.97 (0.96, 0.98) | **<0.001** | 0.97 (0.96, 0.98) | **<0.001** |
| Average objective knowledge about their medical care, mean (SD) | | 71.7 (25.3) | 66.2 (26.0) | **0.023** | 0.99 (0.98, 1) | **0.024** | 0.99 (0.98, 1) | **0.027** |
| Objective understanding of disease, mean (SD) | | 68.42 (33.64) | 64.5 (33.26) | 0.21 | 1 (0.99, 1) | 0.207 | 1 (0.99, 1) | 0.229 |
| Objective understanding of therapeutic approach, mean (SD) | | 72.52 (33.61) | 65.35 (35.44) | **0.026** | 0.99 (0.99, 1) | **0.027** | 0.99 (0.99, 1) | **0.026** |
| Objective understanding of further plans for care, mean (SD) | | 74.01 (33.45) | 68.8 (34.46) | 0.10 | 1 (0.99, 1) | 0.101 | 1 (0.99, 1) | 0.112 |
| **Measured timeliness of ward round** | 466 | | | | | | | |
| Duration of outside the room discussions (min), mean (SD) | | 4.6 (4.8) | 5.2 (5.0) | 0.22 | 1.02 (0.99, 1.06) | 0.215 | 1.05 (0.99, 1.1) | 0.095 |
| Duration of bedside discussions (min), mean (SD) | | 8.7 (5.0) | 9.4 (5.0) | 0.18 | 1.03 (0.99, 1.06) | 0.179 | 1.04 (1, 1.09) | 0.078 |
| Duration of debriefing outside the room (min), mean (SD) | | .53 (1.1) | .57 (1.2) | 0.81 | 1.02 (0.87, 1.2) | 0.807 | 1.03 (0.87, 1.21) | 0.739 |
| Total duration of ward round per patient (min), mean (SD) | | 13.9 (5.6) | 15.1 (5.6) | **0.019** | 1.04 (1.01, 1.08) | **0.02** | 1.05 (1.01, 1.08) | **0.01** |
| **Patient perception regarding time spent on ward round** | 466 | | | | | | | |
| Overall duration of ward round was sufficient (VAS 0-100), mean (SD) | | 95.78 (15.38) | 83.81 (24.55) | **<0.001** | 0.97 (0.95, 0.98) | **<0.001** | 0.96 (0.95, 0.98) | **<0.001** |
| Time spent with physicians was sufficient (VAS 0-100), mean (SD) | | 96.71 (10.02) | 80.66 (22.53) | **<0.001** | 0.92 (0.9, 0.94) | **<0.001** | 0.92 (0.9, 0.94) | **<0.001** |
| Patient estimation of time spent with patient on ward round (min), mean (SD) | | 12.18 (7.18) | 11.26 (6.58) | 0.15 | 0.98 (0.96, 1.01) | 0.154 | 0.98 (0.96, 1.01) | 0.176 |
| Patient estimation of time spent per day with patient case overall (min), mean (SD) | | 82.09 (86.72) | 66.73 (66.44) | **0.031** | 1 (0.99, 1) | **0.036** | 1 (0.99, 1) | **0.035** |
| The ward round was helpful for better | | | | | | | | |
| - understanding the main illness (VAS 0-100), mean (SD) | | 75.03 (32.91) | 54.64 (33.95) | **<0.001** | 0.98 (0.98, 0.99) | **<0.001** | 0.98 (0.98, 0.99) | **<0.001** |
| - further therapeutic measures (VAS 0-100), mean (SD) | | 74.14 (32.45) | 53.03 (33.52) | **<0.001** | 0.98 (0.97, 0.99) | **<0.001** | 0.98 (0.97, 0.99) | **<0.001** |
| - further plans of care (VAS 0-100), mean (SD) | | 83.33 (69.99) | 62.12 (52.73) | **<0.001** | 0.99 (0.98, 0.99) | **<0.001** | 0.99 (0.98, 0.99) | **<0.001** |
| All my questions were answered (VAS 0-100), mean (SD) | | 97.05 (8.55) | 82.10 (24.16) | **<0.001** | 0.92 (0.9, 0.94) | **<0.001** | 0.92 (0.9, 0.94) | **<0.001** |

For peer review only

| | | | | | | | | |
|---|---|---|---|---|---|---|---|---|
| I was able to understand all answers to my questions (VAS 0-100), mean (SD) | | 96.84 (10.74) | 85.85 (20.14) | <0.001 | 0.93 (0.91, 0.95) | <0.001 | 0.93 (0.91, 0.95) | <0.001 |
| Information during visit has been adequate (yes, %) | | 197 (92.9%) | 176 (69.3%) | <0.001 | 0.17 (0.1, 0.31) | <0.001 | 0.17 (0.1, 0.31) | <0.001 |
| Patient did not understand something during the round (yes, %) | | 13 (6.1%) | 36 (14.2%) | 0.005 | 2.53 (1.3, 4.9) | 0.006 | 2.58 (1.32, 5.02) | 0.005 |
| Estimation of my participation in the discussion (VAS 0-100), mean (SD) | | 67.80 (30.61) | 51.78 (29.76) | <0.001 | 0.98 (0.98, 0.99) | <0.001 | 0.98 (0.98, 0.99) | <0.001 |
| Disease was explained in an understandable way (VAS 0-100), mean (SD) | | 91.95 (19.46) | 76.61 (27.1) | <0.001 | 0.97 (0.96, 0.98) | <0.001 | 0.97 (0.96, 0.98) | <0.001 |
| Treatment was explained in an understandable way (VAS 0-100), mean (SD) | | 91.32 (20.51) | 69.49 (31.19) | <0.001 | 0.96 (0.95, 0.97) | <0.001 | 0.96 (0.95, 0.97) | <0.001 |
| Upcoming examinations were explained in an understandable way (VAS 0-100), mean (SD) | | 91.64 (19.12) | 74.34 (28.26) | <0.001 | 0.96 (0.95, 0.98) | <0.001 | 0.96 (0.95, 0.97) | <0.001 |
| The information given during the visit was clear and understandable (VAS 0-100), mean (SD) | | 97.86 (9.04) | 82.92 (22.92) | <0.001 | 0.9 (0.88, 0.93) | <0.001 | 0.9 (0.88, 0.93) | <0.001 |
| **Patient perception regarding discomfort during the ward round** | 466 | | | | | | | |
| Medical terms used during ward round were confusing (VAS 0-100), mean (SD) | | 14.1 (27.87) | 23.13 (30.42) | 0.003 | 1.01 (1, 1.02) | 0.003 | 1.01 (1, 1.02) | 0.003 |
| Ward round discussions made me worry (VAS 0-100), mean (SD) | | 7.93 (22.02) | 15.86 (25.73) | <0.001 | 1.01 (1.01, 1.02) | 0.001 | 1.02 (1.01, 1.02) | 0.001 |
| I felt uncomfortable during ward round (VAS 0-100), mean (SD) | | 3.8 (14.88) | 11.98 (24.44) | <0.001 | 1.02 (1.01, 1.04) | <0.001 | 1.02 (1.01, 1.04) | <0.001 |
| Ward round discussions unsettled me (VAS 0-100), mean (SD) | | 2.23 (9.31) | 12.01 (22.3) | <0.001 | 1.06 (1.03, 1.08) | <0.001 | 1.06 (1.03, 1.08) | <0.001 |
| **Patient perception regarding physician`s behaviour during the ward round** | | | | | | | | |
| Physicians treated me with respect (VAS 0-100), mean (SD) | 466 | 99.31 (3.02) | 91.87 (16.37) | <0.001 | 0.85 (0.8, 0.9) | <0.001 | 0.85 (0.8, 0.9) | <0.001 |
| I was taken seriously (VAS 0-100), mean (SD) | 466 | 98.82 (4.97) | 88.87 (19.54) | <0.001 | 0.89 (0.85, 0.92) | <0.001 | 0.89 (0.85, 0.92) | <0.001 |
| Physicians respected my privacy (VAS 0-100), mean (SD) | 466 | 95.83 (14.64) | 86.64 (21.55) | <0.001 | 0.96 (0.94, 0.98) | <0.001 | 0.96 (0.94, 0.98) | <0.001 |
| Physicians showed compassion (VAS 0-100), mean (SD) | 466 | 83.98 (27.57) | 66.79 (30.85) | <0.001 | 0.98 (0.97, 0.99) | <0.001 | 0.98 (0.97, 0.99) | <0.001 |
| My issues were dealt with discreetly (VAS 0-100), mean (SD) | 466 | 95.01 (14.75) | 83.95 (21.16) | <0.001 | 0.95 (0.94, 0.97) | <0.001 | 0.95 (0.94, 0.97) | <0.001 |
| Some topics during ward round communication caused inconvenience (VAS 0-100), mean (SD) | 466 | 3.66 (15.99) | 10.88 (23.80) | <0.001 | 1.02 (1.01, 1.03) | 0.001 | 1.02 (1.01, 1.03) | 0.001 |
| I was encouraged to address personal topics (VAS 0-100), mean (SD) | 466 | 90.57 (21.35) | 76.54 (27.65) | <0.001 | 0.97 (0.96, 0.98) | <0.001 | 0.97 (0.96, 0.98) | <0.001 |
| My privacy was violated (VAS 0-100), mean (SD) | 466 | 0.88 (5.30) | 5.19 (15.55) | <0.001 | 1.07 (1.02, 1.12) | 0.003 | 1.07 (1.02, 1.12) | 0.003 |
| Teaching took place during ward round (yes, %) | 466 | 61 (28.8%) | 65 (25.6%) | 0.44 | 0.85 (0.57, 1.28) | 0.441 | 0.87 (0.57, 1.33) | 0.51 |
| - if yes , teaching was perceived as disruptive, (VAS 0-100) mean (SD) | 125 | 0.8 (6.4) | 10.3 (29.3) | 0.015 | 1.04 (0.99, 1.08) | 0.09 | 1.04 (1, 1.08) | 0.07 |
| **Patients' perception regarding quality of care (VAS 0-100)** | 466 | | | | | | | |
| I felt "in good hands" in this hospital, mean (SD) | | 96.19 (9.28) | 83.57 (17.04) | <0.001 | 0.91 (0.88, 0.93) | <0.001 | 0.9 (0.88, 0.93) | <0.001 |
| I felt there were contradicting statements from physicians and nursing team, mean (SD) | | 7.58 (20.90) | 18.87 (28.23) | <0.001 | 1.02 (1.01, 1.03) | <0.001 | 1.02 (1.01, 1.03) | <0.001 |
| I feel Confident with the physician team, mean (SD) | | 95.88 (11.54) | 83.79 (18.07) | <0.001 | 0.92 (0.9, 0.94) | <0.001 | 0.92 (0.9, 0.94) | <0.001 |
| I feel Confident with the nursing team, mean (SD) | | 97.45 (6.46) | 86.91 (15.65) | <0.001 | 0.89 (0.87, 0.92) | <0.001 | 0.89 (0.87, 0.92) | <0.001 |
| There is good collaboration of physicians and nurses, mean (SD) | | 95.68 (9.14) | 84.25 (15.8) | <0.001 | 0.91 (0.89, 0.93) | <0.001 | 0.91 (0.89, 0.93) | <0.001 |
| I feel physicians have high competence to treat the current illness, mean (SD) | | 96.35 (9.71) | 82.84 (18.49) | <0.001 | 0.9 (0.88, 0.93) | <0.001 | 0.9 (0.88, 0.93) | <0.001 |
| I feel nurses have high competence to treat the current illness, mean (SD) | | 96.51 (9.16) | 86.39 (31.79) | <0.001 | 0.92 (0.9, 0.94) | <0.001 | 0.93 (0.91, 0.95) | <0.001 |

For peer review only

**adjusted for study centre, intervention. All differences calculated with linear regression models for continuous data. Odds ratios were calculated with logistic regression models. Abbreviations: OR, odds ratio; SD, standard deviation; CI, confidence interval; VAS, visual analogue scale; n, number*

For peer review only

# Supplement 5. Examples of discussions addressing delicate topics

| Number of Example | Sensitive Topic | Predictor | Transcript | Current Location | Intervention group |
|---|---|---|---|---|---|
| 1 | Medical ambiguity | Physician disagrees with/corrects patient | Chief physician (CP): We know that you normally take Diazepam, which has a long half-life…<br>Patient (P): Yes, 24 hours.<br>Chief physician: Even longer, especially if the liver is not working properly. I've seen two people die from Diazepam. We prefer to prescribe Lorazepam<br>Patient: I do not tolerate Lorazepam.<br>Chief physician: What do you mean by “don’t tolerate”?<br>Patient: Once when I withdrew from alcohol and doctors gave me Lorazepam I hallucinated. I saw a mouse in my room. Then I had a delirium and woke up in another hospital.<br>Chief physician: We cannot assume that this was because of the Lorazepam. It was more likely due to the alcohol withdrawal.<br>Patient: Yes, but I've never experienced anything like that with Diazepam.<br>Chief physician: It is important to me that we informed you about this… | bedside | outside |
| 2 | Medical ambiguity | Occurrence of cue | CP: So far, we have tried many different medications, but we cannot solve the issue with drugs alone. In my opinion an intervention is necessary, maybe even two. The goal is to improve the function of your heart and thereby improve your breathing.<br>P: And this intervention is nothing I need to worry about?<br>CP: Well, an intervention is always an intervention. It’s never no risk, right? But it is a low risk I have to say. | bedside | outside |
| 3 | Psychiatric comorbidities | Physician disagrees with/corrects patient | CP: The functioning of your liver is very reduced. After all, it was poisoned by the alcohol, and now you have a cirrhosis.<br>P: A cirrhosis already?<br>CP: Certainly, an early stage of a cirrhosis, yes. If someone does drink such a high amount over such a long time…<br>P: I did drink for 4 weeks only.<br>CP: Yes, but you have a history of 26 years of recurrent relapses.<br>P: Yes, but you did an ultrasound last time and I was told everything was fine?<br>CP: Well, everything fine would be an exaggeration. A photo of someone only gives limited information about her or his character. It's the same with the liver.<br>P: Okay… | bedside | outside |
| 4 | Tumour diagnosis | Distracting/Ignoring emotion | CP: Did anyone close to you have had stomach problems?<br>P: Yes, my siblings have. They had cancer as well. And my husband, of course.<br>CP: Mhm, I am sorry about this.<br>P: Yeah, well no one can do anything about it. I’ll have to deal with it by myself, right? Because it still hurts. It was not long ago.<br>Nurse (N): How long?<br>P: 4 years.<br>CA: What about diarrhoea? Anyone close to you suffered from diarrhoea?<br>P: I don’t know. | bedside | bedside |
| 5 | Social issues (e.g. need for nursing home) | NURSE-Technique, Understanding | Patient: I planned to move in with my partner after my hospital stay. She’s a former nurse, but now a relative of hers has died.<br>Chief physician: Oh dear.<br>Patient (tearful): When she heard what could happen to me, she said no. I mean, over the last years I’ve burdened her with my illness. But now I am really down.<br>Chief physician: Yes, that is… I can understand that.<br>Patient: Now I had to agree with her and promised to look for assisted accommodation.<br>Chief physician: Yes, we can discuss this with our case management team. Or have you already found something?<br>Patient: No, because I expected to move in with her. But now her relative has passed away and it is all so complicated…<br>Chief physician: Yeah, I understand! But unfortunately, we don’t have much time during the ward round so | bedside | outside |

| | | | | | |
|---|---|---|---|---|---|
| | | | we'll have to discuss your further care on another occasion.<br>Patient: Okay, yes, thank you. | | |
| 6 | Non-adherence | Medical staff talks to patient about treatment | CP: Your gastrointestinal tract is attacked by your immune system due to the transplant (GvHD). You will receive some medication against it. However, treatment is kind of tricky as you must take that medication very carefully. To treat this, you need to be more compliant with the medication.<br>P: I did so.<br>CP: Sure…<br>P: We already talked about that last time. I took my medication but at times that were more comfortable for me.<br>CP: This is okay, as long as the time interval between the intake of the Cyclosporine remains the same.<br>P: Yeah, I tried…<br>CP: So, you are aware, that this is important?<br>P: Yes, I am. | bedside | bedside |

For peer review only

# CONSORT 2010 checklist of information to include when reporting a randomised trial

| Section/Topic | Item No | Checklist item | Reported on page No |
|---|---|---|---|
| **Title and abstract** | | | |
| | 1a | Identification as a randomised trial in the title | 1 |
| | 1b | Structured summary of trial design, methods, results, and conclusions (for specific guidance see CONSORT for abstracts) | 3 |
| **Introduction** | | | |
| Background and objectives | 2a | Scientific background and explanation of rationale | 5-6 |
| | 2b | Specific objectives or hypotheses | 6 |
| **Methods** | | | |
| Trial design | 3a | Description of trial design (such as parallel, factorial) including allocation ratio | 6 |
| | 3b | Important changes to methods after trial commencement (such as eligibility criteria), with reasons | n.a. |
| Participants | 4a | Eligibility criteria for participants | See original trial (Becker et al, 2021) |
| | 4b | Settings and locations where the data were collected | 6-8 |
| Interventions | 5 | The interventions for each group with sufficient details to allow replication, including how and when they were actually administered | 7 |
| Outcomes | 6a | Completely defined pre-specified primary and secondary outcome measures, including how and when they were assessed | 8-10 |
| | 6b | Any changes to trial outcomes after the trial commenced, with reasons | n.a. |
| Sample size | 7a | How sample size was determined | See original trial (Becker et al, 2021) |
| | 7b | When applicable, explanation of any interim analyses and stopping guidelines | n.a. |
| Randomisation: | | | |
| Sequence generation | 8a | Method used to generate the random allocation sequence | See original trial (Becker et al, 2021) |
| | 8b | Type of randomisation; details of any restriction (such as blocking and block size) | See original |

For peer review only


For peer review only - http://bmjopen.bmj.com/site/about/guidelines.xhtml

| | | | |
|---|---|---|---|
| | | | trial (Becker et al, 2021) |
| Allocation concealment mechanism | 9 | Mechanism used to implement the random allocation sequence (such as sequentially numbered containers), describing any steps taken to conceal the sequence until interventions were assigned | See original trial (Becker et al, 2021) |
| Implementation | 10 | Who generated the random allocation sequence, who enrolled participants, and who assigned participants to interventions | See original trial (Becker et al, 2021) |
| Blinding | 11a | If done, who was blinded after assignment to interventions (for example, participants, care providers, those assessing outcomes) and how | 7, see also at original trial (Becker et al, 2021) |
| | 11b | If relevant, description of the similarity of interventions | See original trial (Becker et al, 2021) |
| Statistical methods | 12a | Statistical methods used to compare groups for primary and secondary outcomes | 10 |
| | 12b | Methods for additional analyses, such as subgroup analyses and adjusted analyses | 10 |
| **Results** | | | |
| Participant flow (a diagram is strongly recommended) | 13a | For each group, the numbers of participants who were randomly assigned, received intended treatment, and were analysed for the primary outcome | See original trial (Becker et al, 2021)/ 12(Table1) |
| | 13b | For each group, losses and exclusions after randomisation, together with reasons | See original trial (Becker et al, 2021) |
| Recruitment | 14a | Dates defining the periods of recruitment and follow-up | 6 |
| | 14b | Why the trial ended or was stopped | n.a. |
| Baseline data | 15 | A table showing baseline demographic and clinical characteristics for each group | 12 (Table1) |
| Numbers analysed | 16 | For each group, number of participants (denominator) included in each analysis and whether the analysis was by original assigned groups | See original trial (Becker et al, 2021) |
| Outcomes and estimation | 17a | For each primary and secondary outcome, results for each group, and the estimated effect size and its precision (such as 95% confidence interval) | 16-18 |


For peer review only - http://bmjopen.bmj.com/site/about/guidelines.xhtml
For peer review only

| | | | |
|---|---|---|---|
| | 17b | For binary outcomes, presentation of both absolute and relative effect sizes is recommended | |
| Ancillary analyses | 18 | Results of any other analyses performed, including subgroup analyses and adjusted analyses, distinguishing pre-specified from exploratory | 14-18 |
| Harms | 19 | All important harms or unintended effects in each group (for specific guidance see CONSORT for harms) | n.a. |
| **Discussion** | | | |
| Limitations | 20 | Trial limitations, addressing sources of potential bias, imprecision, and, if relevant, multiplicity of analyses | 26-27 |
| Generalisability | 21 | Generalisability (external validity, applicability) of the trial findings | 26-27 |
| Interpretation | 22 | Interpretation consistent with results, balancing benefits and harms, and considering other relevant evidence | 27 |
| **Other information** | | | |
| Registration | 23 | Registration number and name of trial registry | 4 |
| Protocol | 24 | Where the full trial protocol can be accessed, if available | 7 |
| Funding | 25 | Sources of funding and other support (such as supply of drugs), role of funders | 29-30 |

For peer review only
